# Optoelectrical Conditioning in Graphene Quantum Dot‍–‍Vanadium Oxide Artificial Synapse

**DOI:** 10.1002/smsc.70434

**Published:** 2026-10-27

**Authors:** Prabana Jetty, Somnath S. Kundale, Yong Tae Kim, Windy Ayu Lestari, Sang Won Lee, Jaeyeong Heo, Jun Hong Park

**Affiliations:** ^1^ Department of Materials Engineering and Convergence Technology Gyeongsang National University Jinju Gyeongsangnam‐do Republic of Korea; ^2^ Research Institute for Green Energy Convergence Technology Gyeongsang National University Jinju Republic of Korea; ^3^ Department of Materials Science and Engineering, and Optoelectronics Convergence Research Center Chonnam National University Gwangju Republic of Korea

**Keywords:** graphene quantum dots, memristors, neuromorphic computing, optoelectrical conditioning, vanadium oxide

## Abstract

Neuromorphic computing requires artificial synaptic devices capable of adaptive learning and memory retention. Here, we present a monolithically integrated optoelectronic synaptic device based on a 0D/3D Ag/graphene quantum dot–vanadium oxide (GQD–VO_2_)/Ni heterojunction exhibiting stimulation‐history‐dependent conductance modulation. The device supports synaptic modulation under electrical, optical, and combined optoelectrical stimulation, with enhanced optoelectronic response compared with the bare VO_2_ device. Repeated optoelectrical stimulation modifies the retained device state and subsequent potentiation–depression characteristics, a process described here as optoelectrical conditioning. This evolution is evaluated using retained current (*I*), fitted potentiation–depression time constants (*τ*), and nonlinearity coefficients. Wavelength‐dependent optical associative learning with positive and negative reinforcement is further demonstrated under different optical stimuli. Performance evaluation using Fashion‐MNIST and Digit‐MNIST datasets show improved classification accuracy with the progression of optoelectrical conditioning learning cycles, increasing from 81.53% to 84.13% and from 91.71% to 95.58%, respectively. Monte Carlo simulations further indicate improved classification accuracy across multiple runs following repeated learning cycles. Furthermore, this behavior is illustrated through reconstructed Fashion‐MNIST image outputs with the repeated learning cycles. These findings highlight the potential of Ag/GQD–VO_2_/Ni device for neuromorphic applications, advancing AI‐integrated bio‐inspired computing.

## Graphical abstract

A 0D/3D heterojunction device based on Ag/GQD–VO_2_/Ni architecture mimics key aspects of human visual processing through optoelectrical conditioning and optical associative learning. Repeated optoelectrical stimulation induces history‐dependent conductance modulation, leading to improved synaptic characteristics and pattern recognition performance. These findings demonstrate the potential of the device for low‐power, bio‐inspired neuromorphic computing systems.

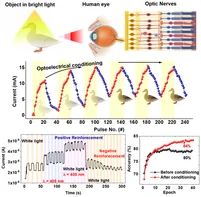

## 1 Introduction

Rapid advancements in artificial intelligence (AI) and neuromorphic computing have driven the development of bioinspired electronic systems that mimic biological sensory and cognitive functions [1, 2, 3]. Among various neuromorphic functions, optoelectronic synapses are particularly promising for integrating optical and electrical stimuli, enabling memory formation and associative learning in artificial vision systems [4, 5]. These functions are relevant to next‐generation robotics, human–machine interfaces, and sensory systems for intelligent computing [5, 6]. In biological systems, the human visual system processes and retains sensory information through short‐term, mid‐term, and long‐term memories. Photopic adaptation enables the visual system to adjust to changes in the light intensities, while wavelength‐dependent perception is associated with cone‐cell responses [7]. Similarly, associative learning plays a fundamental role in memory formation, in which exposure to multiple stimuli strengthens synaptic connections over time [8]. Replicating these processes in artificial visual systems is challenging, particularly in achieving scalable, low‐power, and multimodal operations [9].

In the human visual system, photosensitive retinal cells convert optical stimuli from the external environment into electrical impulses [10]. These signals are then transmitted through the optic nerves, a network of neurons and synapses, to the brain for processing. To replicate this process, artificial visual systems require the integration of photodetectors and synaptic devices capable of detecting light, along with processing and storing sensory information in a manner similar to biological vision [10, 11].

Recent research efforts have focused on developing memristors and neuromorphic transistors based on metal oxides and quantum dots (QDs), which enable color‐selective imaging and neuromorphic processing. However, hybrid heterojunction architectures integrating 0D QDs have received increasing attention due to their ability to enhance interfacial charge modulation and defect engineering [12, 13]. Such mixed‐dimensional systems enable low‐power operation [14, 15], multilevel conductance modulation, and efficient optoelectronic interaction, offering promising potential for neuromorphic computing platforms [16, 17, 18, 19, 20, 21]. Among these, VO_2_ and QD heterostructures have demonstrated diverse optoelectronic synaptic functions, including optical synaptic plasticity, short‐term to long‐term plasticity transition, adaptation, associative learning, and visual perception related responses [22, 23, 24, 25]. However, the influence of history‐dependent conductance modulation under repeated optoelectrical stimulation and their relationship with learning dynamics has received comparatively limited quantitative attention. Although optoelectrical conditioning behaviors have been reported in artificial synaptic devices, studies that systematically correlate repeated optoelectrical stimulation with history‐dependent changes in conductance modulation, synaptic learning dynamics, and neuromorphic task performance remain limited [11, 26, 27]. Addressing this challenge may support the development of bio‐inspired neuromorphic systems.

In this study, we investigate optoelectrical conditioning in a monolithically fabricated GQD–VO_2_ synaptic device linking the evolution of its conductance modulation characteristics to improved performance in neural‐network simulations. Unlike conventional CMOS‐based sensor memory architectures that require external circuit integration [28, 29], the proposed two‐terminal Ag/GQD‍–‍VO_2_/Ni device enables optical sensing, memory storage, and adaptive modulation within a single structure. The engineered 0D–3D heterointerface combines the complementary functions of GQDs as photonic sensitizers and photocarrier‐trapping centers with VO_2_ as the active filamentary switching medium, resulting in an optically tunable memory window (~10^4^), enhanced responsivity (0.29 A/W), improved analog linearity (*R* ^2^: 0.81–0.97), and multilevel conductance states. Compared with the bare VO_2_ devices, which exhibit weak photoresponse and a narrow dynamic range (2.06), the GQD–VO_2_ heterojunction broadens the dynamic range (44.29), reduced the optoelectronic energy consumption (from 220 to 31.5 nJ per spike), and improves optoelectronic response and conductance linearity.

The device provides a platform for examining how repeated optoelectrical stimulation changes the retained device state and subsequent conductance modulation. We quantify these changes using cycle‐resolved electrical responses, fitted potentiation–depression time constants, and nonlinearity coefficients. This behavior is quantitatively evaluated through quantitative analysis of conductance states, learning and forgetting time constants (*τ*), and synaptic linearity. Furthermore, wavelength‐dependent optical associative learning is demonstrated through dynamic modulation of synaptic weights under different light stimuli. The positive and negative reinforcement behavior were demonstrated under optical stimulation. To further validate the neuromorphic performance of the proposed device, Fashion‐MNIST and Digit‐MNIST tasks were evaluated, showing accuracy improvement after optoelectrical conditioning from 81.53% to 84.13% and 91.71% to 95.58%, respectively. This improvement is supported by the Monte Carlo (MC) simulations, confirming consistent performance enhancement. In addition, optoelectrical conditioning was visually confirmed through reconstructed Fashion‐MNIST images with six learning cycles, demonstrating enhanced pattern recognition and image reconstruction capability. These results indicate that the Ag/GQD–VO_2_/Ni device is promising for neuromorphic implementation, offering a route towards bio‐inspired computational frameworks.

## 2 Results and Discussion

### 2.1 Synaptic Properties of the Ag/GQD–VO_2_/Ni Memristors

The human visual system performs the highly complex task of recognizing images under varying light intensities and wavelengths, adapting to different illumination conditions, such as dark and bright environments. This adaptation involves retinal photoreceptor cells, where cone cells dominate under bright‐light (photopic) conditions [30]. During photopic adaptation, repeated or sustained exposure to bright illumination results in an adjustment of visual sensitivity, enabling effective perception under the prevailing light environment [7, 31]. Inspired by this history‐dependent adjustment, we introduce optoelectrical conditioning in the artificial synaptic device, where repeated optical and electrical stimulation progressively modifies the device response and eventually leads to an adaptation‐like stabilized state [30, 32]. Figure 1a illustrates the conceptual framework of visual adaptation, highlighting the response of the human visual system to bright light and specific wavelength exposure through neural learning. To characterize the synthesized VO_2_ thin films, we performed X‐ray diffraction analysis (Figure 1b). The peaks at 14.3°, 25.2°, 29°, 44.2°, and 49.4° correspond to the (001), (110), (002), (003), and (020) planes, and well match with monoclinic VO_2_ phase (JCPDS card No. 01‐81‐2392) [33]. In addition, Raman spectroscopy was performed for the VO_2_ and GQD–VO_2_ films (Figure 1c). The characteristic Raman peak at 812 cm^−1^, corresponding to the V═O stretching mode, was observed in both spectra, confirming the successful deposition of VO_2_ [34]. The Raman peak at 575 cm^−1^ corresponds to V–O stretching (A_g_) consistent with previously reported vibrational mode classification [35, 36]. In the Raman spectrum of the GQD–VO_2_ film (red line in Figure 1c), the shoulder peak at ~747 cm^−1^ corresponds to the V–O stretching mode (B_g_) due to the defect modified in VO_2_ film after introducing GQD, resulting in mode splitting [37]. Two distinct peaks are observed at 1340 and 1560 cm^−1^, corresponding to the D and G bands of graphene, respectively [38, 39]. These values are consistent with those reported in the literature, further validating the presence of GQDs in the heterojunctions. The UV‍–‍visible absorption spectra of the VO_2_ and GQD–VO_2_ samples are recorded to investigate their optical absorption characteristics (Figure 1d). The GQD sample exhibited distinct absorption peaks at 255 and 368 nm (black spectrum) [40, 41], whereas pristine VO_2_ exhibited a peak at approximately 370 nm (red spectrum) [42, 43]. Upon the deposition of GQDs onto VO_2_, the GQD–VO_2_ hybrid exhibited two prominent absorption peaks at 258 and 402 nm, corresponding to GQDs and VO_2_, respectively. In addition, the hybrid exhibited a broad absorption range from 300 to 450 nm. Based on the absorption profile of the GQD‍–‍VO_2_ sample, a 405 nm laser was selected for optical stimulation. Cross‐sectional transmission electron microscopy (TEM) imaging combined with elemental mapping is performed to analyze the layer structure and thicknesses of the fabricated Ag/GQD–VO_2_/Ni device (Figure S1), as shown in Figure 1e. The TEM cross‐section reveals a multilayer structure, comprising a 104 nm top Ag electrode, a 550 nm GQD layer, a 31 nm VO_2_ layer, and a 181 nm Ni bottom electrode. The ~550 nm GQD thickness corresponds to the coating obtained under the selected drop‐casting condition rather than the effective carrier‐transport distance. The GQD layer contributes to optical absorption and photocarrier generation/trapping, while electronic modulation of VO_2_ is expected to occur near the GQD/VO_2_ interface. The elemental mapping of Ag, C, V, O, and Ni confirms the uniform deposition of each layer and highlights the compositional consistency across the device structure (Figure 1f). Therefore, the GQD/VO_2_ interface forms a 0D/3D multidimensional heterojunction, structurally validated by TEM and elemental mapping. X‐ray photoelectron spectroscopy (XPS) further confirmed the elemental composition and phase of VO_2_. Figure S2 shows the survey spectrum and deconvolution of the VO_2_ film. A detailed discussion of the XPS analysis, including the peak deconvolution and oxidation state estimations, is provided in the Supporting Information [44, 45, 46].

**FIGURE 1:**
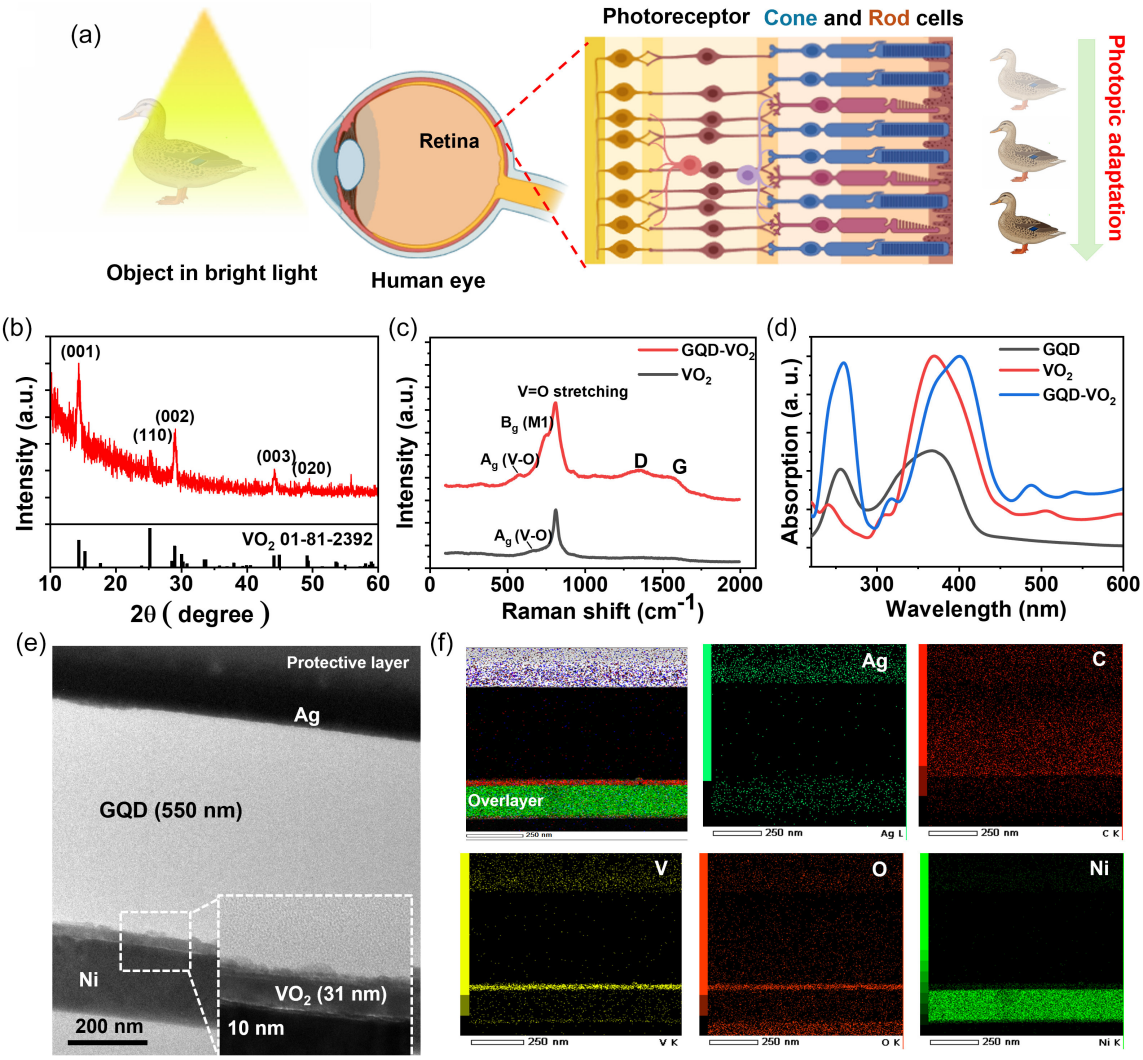
(a) Conceptual of visual adaptation in the human visual system. (b) X‐ray diffraction (XRD) of VO_2_. (c) Raman spectra of VO_2_ and graphene quantum dot (GQD)–VO_2_. (d) Absorption spectra of GQD, VO_2_, and GQD–VO_2_. (e) Cross‐sectional TEM images and (f) corresponded to elemental mapping of Ag/GQD–VO_2_/Ni device fabricated via monolithic integration.

Figure 2a illustrates the voltage‐dependent current–voltage (*I*‍–‍*V*) characteristics of the Ag/VO_2_/Ni device. The device exhibits notable analog resistive switching within the voltage range of ±0.5 to ±1 V, highlighting its potential for low‐power neuromorphic applications [47]. Owing to the strong absorption of VO_2_ in the UV–‍visible spectral range, the Ag/VO_2_/Ni device exhibits laser power‐dependent *I*–*V* characteristics, as detailed in Figure S3. Voltage‐dependent endurance and retention behaviors of the device are shown in Figure S3, which highlights its stability and reliability under varying operational conditions. To enhance the electrical properties and optical response, we fabricated an Ag/GQD–VO_2_/Ni device by drop‐casting GQDs onto VO_2_. Figure 2b shows the voltage‐dependent *I*–*V* characteristics of the Ag/GQD‍–‍VO_2_/Ni device. In contrast to the bare VO_2_ device, this configuration demonstrates an increased area under the curve as the voltage range expands from ±0.5 to ±1 V. The memory window at 1 V for the *I*–*V* characteristics of the Ag/GQD–VO_2_/Ni device was significantly larger than that of the Ag/VO_2_/Ni device, providing a wide range for multilevel conductance and a linear increment in current (compared with the VO_2_ device) with SET bias. The device‐to‐device *I*–*V* characteristics and cycle‐to‐cycle variability of Ag/GQD–VO_2_/Ni devices were analyzed on 20 independent devices, revealing good reproducibility, as illustrated in Figures S4 and S5. At higher cycle counts, no abrupt device failure was observed, but the distinction between low‐resistance state (LRS) and high‐resistance state (HRS) states is narrowed. It can be due to partial retention of the conductive filament, or it can be due to defect accumulation in the switching layer [48]. We also studied the influence of different electrode area on the Ag/GQD–VO_2_/Ni device (Figure S6). The photoactive *I‍*–‍*V* characteristics of the Ag/GQD–VO_2_/Ni device are then measured under laser irradiation (405 nm) (Figure 2c). Notably, the device exhibited digital SET and analog RESET behaviors with a large memory window (approximately 10^4^). As the laser power increased, the SET voltage decreased, but the HRS shifted to a higher current, effectively distinguishing the conductance states across varying power levels. Notably, the Ag/GQD–VO_2_/Ni device exhibited analog behavior in the electrical mode and digital behavior in the combined optical–electrical mode. In addition, the number of conductance states increases owing to the measured wide memory window. Figure 2d shows the voltage‐dependent multilevel endurance (0.5–1 V), which shows stable conductance states up to 500 cycles. Figure 2e shows the multilevel retention of the Ag/GQD–VO_2_/Ni device (0.5–1 V), revealing significant stability of different numbers of states over time. Linearity in the voltage‐dependent conductance states is a critical parameter for reliable synaptic behavior in neuromorphic computing systems. To evaluate this, we extracted the SET bias‐dependent current values for the Ag/VO_2_/Ni and Ag/GQD–VO_2_/Ni devices, as shown in Figure 2a,b, and summarized in Figure 2f. Linear fitting of these SET bias‐dependent currents yielded an adjusted *R* ^2^ value of 0.81 for the Ag/VO_2_/Ni device and an improved value of 0.97 for the Ag/GQD–VO_2_/Ni device. This linearity enhancement highlights the crucial role of GQDs in modulating the heterojunction interface, facilitating more linear and controllable conductance switching. These results underscore the importance of GQDs in optimizing the voltage‐dependent synaptic performance for neuromorphic applications.

**FIGURE 2:**
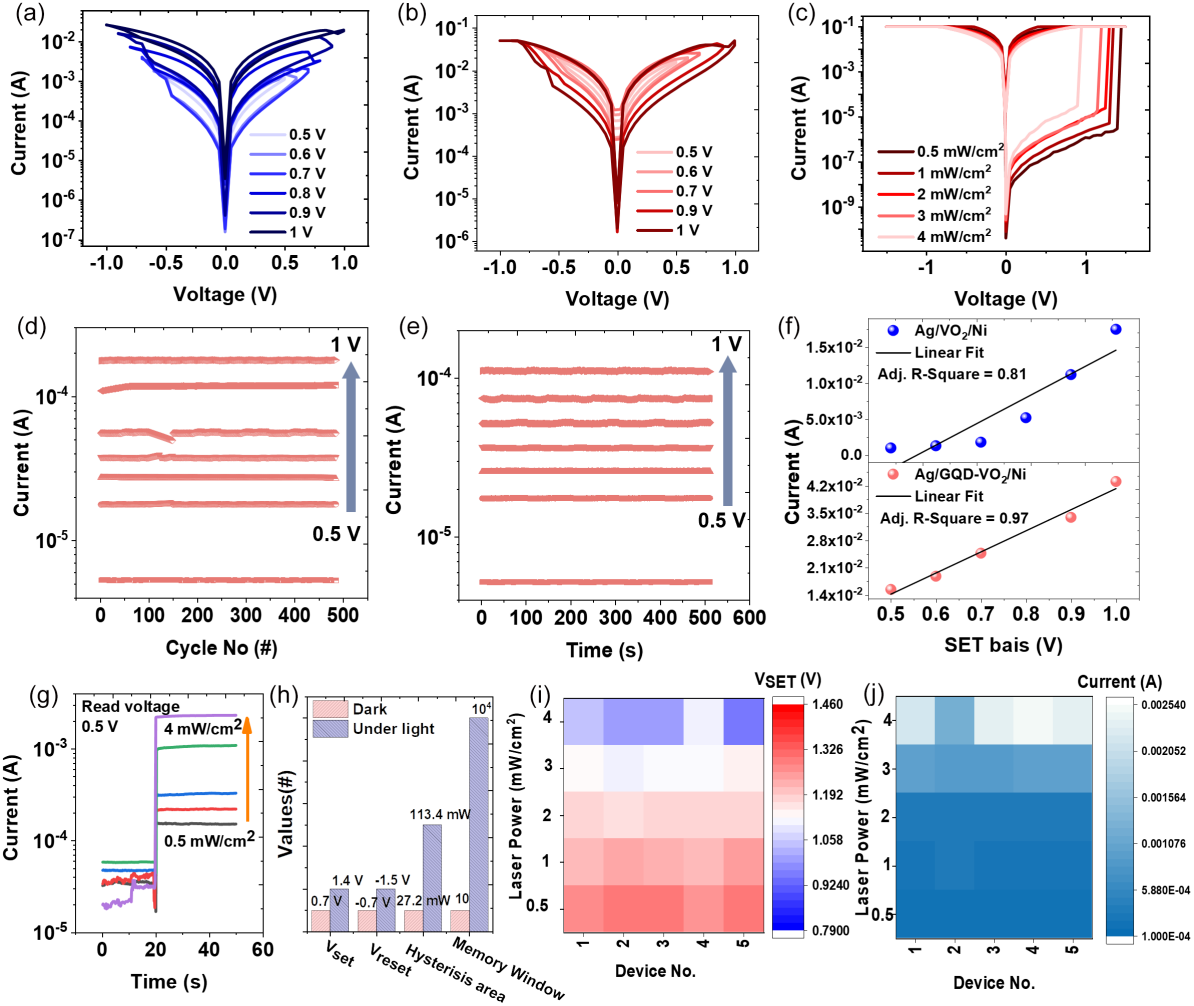
Voltage‐dependent current–voltage (*I*–*V*) characteristics of (a) Ag/VO_2_/Ni device and (b) Ag/GQD–VO_2_/Ni device. (c) Photoactive *I*–*V* characteristics of the Ag/GQD–VO_2_/Ni device measured under laser irradiation (405 nm). (d) Voltage‐dependent endurance of the Ag/GQD‍–‍VO_2_/Ni device showing multilevel conductance. (e) Voltage‐dependent retention of the Ag/GQD–VO_2_/Ni device. (f) SET bias‐dependent increment in current in Ag/VO_2_/Ni and Ag/GQD–VO_2_/Ni devices. (g) Optical response of the Ag/GQD–VO_2_/Ni device under varying laser power. (h) Summarizes the *V*_SET_, *V*_RESET_, hysteresis area, and memory window for the Ag/VO_2_/Ni and Ag/GQD–VO_2_/Ni devices. (i) SET voltages of five devices under laser powers ranging from 0 to 4 mW/cm^2^. (j) The conductance states were measured under different laser powers in five devices.

The optical response of the Ag/VO_2_/Ni and Ag/GQD–VO_2_/Ni device under varying laser power levels is shown in Figures S7a and 2g, respectively, demonstrating the changes in the resistance states with increasing laser power (0.5–4 mW/cm^2^). Based on the optical response, we calculated the average responsivity of the devices. The Ag/GQD–VO_2_/Ni device shows 0.29 A/W, higher than that of the Ag/VO_2_/Ni device (0.07 A/W), as shown in Figure S7b. These results indicate the contribution of the GQD–VO_2_ heterostructure to optoelectronic applications. A comparison of the performance parameters provides further insights into the device characteristics. Figure 2h summarizes the *V*_SET_, *V*_RESET_, hysteresis area, and memory window of Ag/GQD–VO_2_/Ni devices under dark and 405 nm illumination, respectively, extracted from Figure 2b,c. The *V*_SET_ and *V*_RESET_ values were higher for the Ag/GQD–VO_2_/Ni device under illumination than in the dark and were influenced by optical stimuli. *V*_SET_ increased from 0.7 to 1.4 V, and *V*_RESET_ increased from −0.7 to −1.5 V under illumination. However, the hysteresis area (27.2–113.4 mW) and memory window (10–10^4^) were also significantly increased under illumination, highlighting the optoelectronic effect of GQD deposition on VO_2_. Furthermore, device‐to‐device variation, which is a critical factor affecting repeatability in memristor performance, is evaluated by measuring the SET voltages of five devices under laser powers ranging from 0.5 to 4 mW/cm^2^ (Figure 2i). The SET voltage variation decreased with increasing laser power and remained consistent across all five devices. To analyze the conductance‐state variability further, we measured the conductance states at different laser powers for the five devices (Figure 2j). Overall, the Ag/GQD–VO_2_/Ni device exhibited reproducible optoelectronic switching characteristics, suggesting its potential for optoelectronic applications [49]. Thickness‐dependent measurements comparing GQD layers of ~125 nm (2 μL) and ~550 nm (20 μL) showed that increasing the GQD thickness did not proportionally enhance the switching response, suggesting that the device behavior is not solely governed by the bulk GQD layer and indicating a possible contribution from the GQD/VO_2_ interface (Figure S8).

Based on the electrical and optical *I*–*V* characteristics of the Ag/GQD–VO_2_/Ni device, a possible resistive switching mechanism was proposed. In the optoelectrical mode, at low voltages, the Ag top electrode undergoes oxidation, generating Ag^+^ ions that migrate toward the negatively biased Ni bottom electrode. Simultaneously, oxygen vacancies (*V*_0_) in the VO_*x*_ layer redistribute under the applied electric field, contributing to the formation of a conductive filament [50]. Under low‐power optical illumination, photoelectrons are generated within the GQD layer; however, these carriers may initially become trapped at defect sites of GQD (Figure 3a) rather than directly participating in filament formation. Consequently, a higher electric field is required to fill these trap states before sufficient free carriers become available to initiate filament growth [51]. This trap‐filling process contributes to the observed increase in *V*_set_. The presence of defects in the GQDs is confirmed by the Raman spectra, as shown in Figure S9. The 2D peak at 2677 cm^−1^ provides information about structural characteristics of GQDs, suggesting possible charge‐trapping sites [17]. However, the number of photoexcited electrons increases with increasing laser power. Notably, the GQDs exhibit fluorescence, emitting light in the 450–550 nm range when excited by a 400–410 nm laser, which coincides with the absorption wavelength of VO_2_ [52]. Under optical illumination, photocarriers are generated and trapped within the GQD layer. Carriers generated in the vicinity of the GQD/VO_2_ interface would directly contribute to interfacial charge modulation, whereas GQDs farther from the interface primarily contribute to optical absorption, carrier trapping, and photonic sensitization. In addition, the fluorescence emission of GQDs overlaps with the absorption range of VO_2_, suggesting an additional optical excitation pathway for the underlying VO_2_ layer. The resulting photoinduced carrier modulation, together with Ag‐ion and oxygen‐vacancy migration, influence conductive filament formation and the optoelectrical switching characteristics. A fluorescence image of the GQDs, confirming this effect, is provided in Figure S10. This photoinduced charge‐carrier generation significantly increases the device current with increasing optical power, which facilitates filament formation and reduces the external electric field [53]. As a result, *V*_set_ decreases with increasing illumination intensity. Therefore, at a set voltage under optical illumination, conductive filament formation occurs through the combined participation of Ag ions, oxygen vacancies, and photoelectrons. This process, along with electron detrapping, results in charge neutralization at the interface, switching the device from a HRS to a LRS [54]. Upon applying a reverse voltage, the conductive filament ruptures owing to the trapping of electrons, back migration of Ag ions, and oxygen vacancies, restoring the device to the HRS. To gain a deeper understanding of the charge transport behavior, the high‐voltage region of the *I*–*V* characteristics of the Ag/GQD–VO_2_/Ni device was analyzed under dark and illuminated conditions, as shown in Figure S11a,b. In the dark, the high‐voltage region is well fitted by the Child’s square‐law mechanism, indicating space‐charge‐limited conduction. In contrast, under illumination, the high‐voltage region is better described by the Poole–Frenkel (P–F) emission model, suggesting field‐enhanced carrier emission from trap states [17]. Figure S11c schematically illustrates the proposed trap‐assisted transport under illumination. Overall, the GQD‍–‍VO_2_ heterostructure exhibits optoelectronic resistive switching characteristics, where photogenerated charge carriers contribute to the modulation of switching behavior, suggesting its potential for optoelectrical neuromorphic computing applications.

**FIGURE 3:**
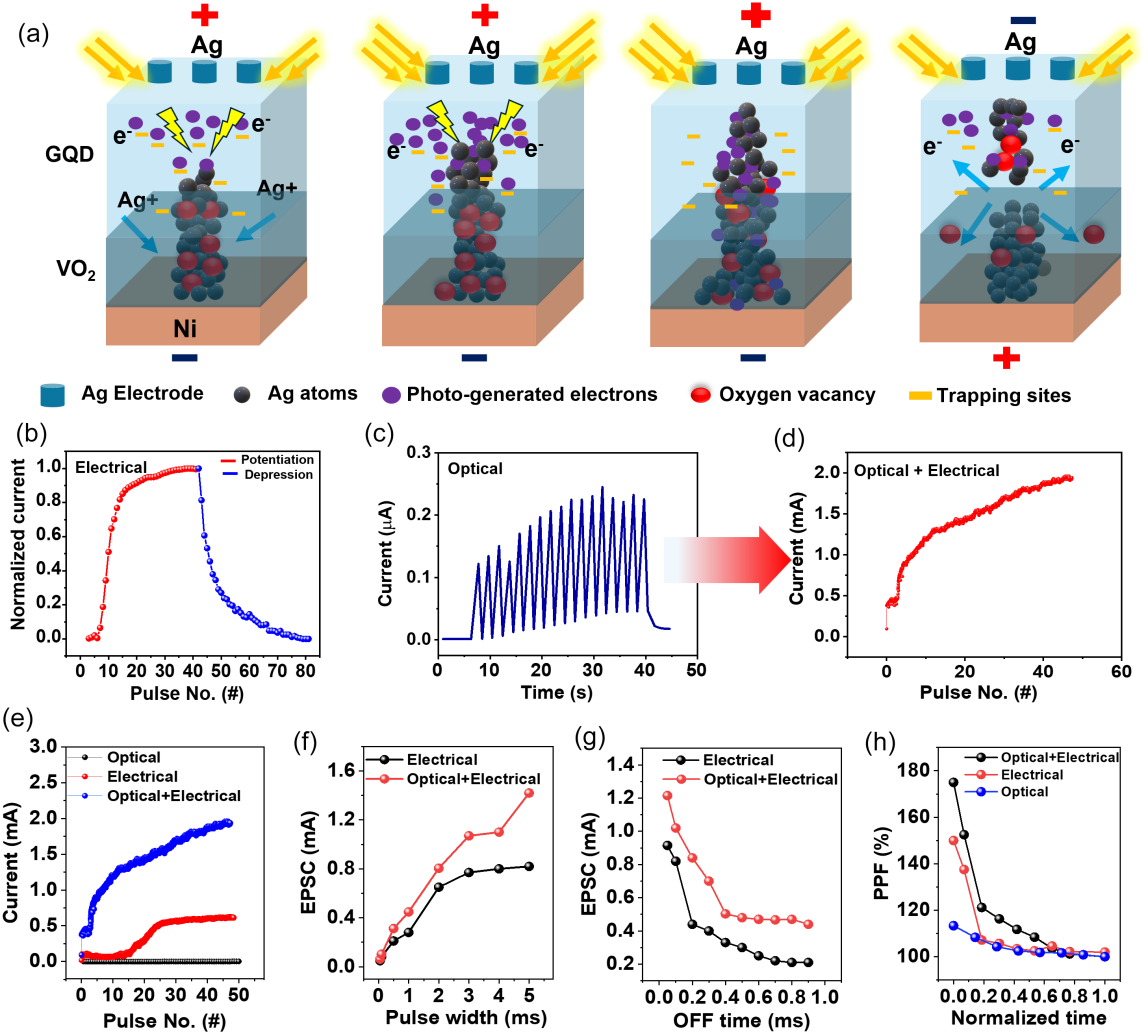
(a) Proposed Resistive switching mechanism of the Ag/GQD–VO_2_/Ni device. (b) Potentiation and depression results for Ag/GQD–VO_2_/Ni device. (c) Postsynaptic current (PSC) of the Ag/GQD–VO_2_/Ni device under 405 nm wavelength laser irradiation at 4 mW/cm^2^. (d) Combined effect under electrical and optical pulses on potentiation. (e) Comparison of potentiation behavior under optical, electrical, and combined optical–electrical stimulation. (f) PSC responses after increasing pulse width up to 5 ms. (g) Excitatory postsynaptic current (EPSC) after increasing the off time of the applied electrical pulse in dark and optical modes. (h) PPF index of Ag/GQD–VO_2_/Ni device in three modes.

The synaptic characteristics were initially demonstrated in an Ag/VO_2_/Ni device. The optical synaptic behavior was evaluated by measuring the PSC response of VO_2_, which exhibits a relatively weak photoresponse (Figure S12). Subsequently, the potentiation (+1 V, 50 μs) and depression (−1 V, 50 μs) were evaluated under both electrical and optoelectrical stimulation (Figure S13). The optoelectrical measurements exhibit enhanced current compared to electrical operation. Building on this, synaptic characteristics were determined by application of electrical pulses +1 V (40 μs) and −1 V (150 μs) on the Ag/GQD–VO_2_/Ni device, as shown in Figure 3b. During potentiation at (+1 V), the trapped charges accumulate and increase the synaptic weight. During depression (−1 V), the charges slowly detrap, giving gradual decay and longer retention. The corresponding excitatory postsynaptic current (EPSC) and inhibitory postsynaptic current (IPSC) responses are shown in Figure S14a,b, demonstrating the Ag/GQD–VO_2_/Ni device’s learning and forgetting capabilities. Linearity is another crucial parameter of synaptic properties, with higher linearity indicating superior performance. The nonlinearity of the Ag/GQD–VO_2_/Ni device during electrical stimulation is ~0.1, highlighting its suitability for synaptic applications (Figure S14c). [55]

The optical tuning of conductance states benefits optoelectronic devices, particularly for retinomorphic applications. To explore this, we measured the EPSC of the Ag/GQD–VO_2_/Ni device under 405 nm laser irradiation at an intensity of 4 mW/cm^2^ (pulse width 2 s) (Figure 3c) [56]. The results indicated that the EPSC increased gradually with the application of successive optical pulses, with a non‐monotonic response due to trapping and detrapping behavior. Subsequently, we investigated the combined effect of electrical pulses with continuous optical illumination on Ag/GQD–VO_2_/FTO device, with the input parameters detailed in supporting Information (Figure S14d). An enhanced EPSC response was observed, shown in Figure 3d [57]. This enhancement is associated with the nanoscale dimensions and quantum confinement effects of GQDs, which can introduce surface and edge states that act as potential trapping sites for photoexcited and electrically injected carriers. Additionally, the energy consumption per synaptic event or spike was also calculated in the Supporting Information for the Ag/GQD–VO_2_/Ni device under optoelectrical operation and was found to be 31.5 nJ/spike. A comparative analysis with the Ag/VO_2_/Ni device is provided in Table S1. Notably, the Ag/GQD–VO_2_/Ni device exhibits low energy consumption compared to the pristine VO_2_ device. Further device‐to‐device variations in synaptic nonlinearity and energy consumption are provided in Figure S15. To compare the effect of the three stimulation methods, we analyzed potentiation behavior under optical, electrical, and optoelectrical stimulations (Figure 3e). The dynamic range of potentiation was relatively small under optical stimulation, increased under electrical stimulation, and was further enhanced by optoelectrical stimulation [58]. The comparative analysis of the dynamic range of Ag/VO_2_/Ni and Ag/GQD–VO_2_/Ni devices is provided in Table S1. However, a tradeoff in potentiation linearity was observed, which decreased gradually across the stimulation modes. This finding suggests that the device exhibits a higher current operating range under combined optoelectrical stimulation, indicating its potential for neuromorphic applications. Furthermore, we modulated the pulse duration‐dependent EPSC response induced by electrical and optoelectrical stimuli. This response was determined by the width of the electrical pulse. Notably, the EPSC saturated during electrical stimuli with increased pulse width after 2 ms. However, EPSC gradually increases with increasing pulse width up to 5 ms in the optoelectrical mode (Figure 3f) [59], underlining the optical stimulation effect on pulse duration‐dependent EPSC. Similarly, we measured the EPSC by increasing the off‐time of the applied electrical pulse in dark and optical modes. Both modes became saturated after 0.4 ms; however, the combined optoelectrical mode EPSC current was higher than the electrical current (Figure 3g). The PPF index represents spike‐time‐dependent plasticity [60]. The PPF percentage in the combined optoelectrical mode was comparatively higher than that observed in the electrical and optical modes, which gradually decreased with increasing time (Figure 3h) [61]. The optoelectrical mode provides a broad synaptic modulation range and can modulate synaptic weights across multiple states [62].

### 2.2 Optoelectrical Conditioning in the Ag/GQD‍–‍VO_2_/Ni Device

Cyclic stability during long‐term potentiation (LTP) and long‐term depression (LTD) is crucial for artificial synaptic devices [63]. To evaluate this aspect, we measured multiple consecutive potentiation and depression cycles in electrical and optoelectrical modes using differential pulses (0.2–1.2 V). Figure 4a shows six consecutive potentiation and depression cycles for the Ag/GQD‍–‍VO_2_/Ni device under electrical stimulation. Conductance variations exhibited consistent potentiation and depression behaviors within the same range, demonstrating stable synaptic performance. The average standard deviation (SD) over 6 cycles (Figure 4a) is ~1.34 mS, indicating uniformity and stable synaptic behavior across multiple cycles and devices, as illustrated in Figure S16. To further assess device‐to‐device reproducibility, potentiation and depression were extended to 20 cycles on a different device, as shown in detail in Figure S17. The electrical pulse parameters were 1 V pulse amplitude for potentiation and −1 V for depression. To further investigate the response of the device to optoelectrical stimulation, we applied a 405 nm laser (4 mW/cm^2^) as an optical stimulus and recorded the potentiation and depression cycles. Figure 4b illustrates the six consecutive cycles under the optoelectrical mode. After each potentiation–depression cycle, the device retained a current above its initial level, resulting in a progressive increase in the baseline during the early cycles (Figure 4b) [31]. During the later cycles, the baseline showed smaller changes, while the potentiation–depression characteristics also evolved with stimulation history. We describe this history‐dependent evolution of the device state and subsequent conductance modulation as optoelectrical conditioning. The following analysis quantifies this evolution using cycle‐resolved responses, fitted time constants, and nonlinearity coefficients. Biological visual adaptation illustrates how prior illumination influences subsequent sensory responses, providing the conceptual inspiration as shown in Figure 4c. In the Ag/GQD–VO_2_/Ni device, repeated optoelectrical stimulation similarly modifies the retained device state and subsequent synaptic responses. We describe this device‐level behavior as optoelectrical conditioning, as shown in Figure 4c. During this process, photogenerated carriers progressively accumulate and become trapped at defect sites within the GQD layer, while applied electrical pulses can simultaneously promote Ag^+^ ion migration and oxygen vacancy redistribution‐assisted conductive filament reinforcement in the VO_2_ layer. Consequently, the device does not completely return to its initial current state during depression, resulting in a retained current after each learning cycle. With successive optoelectrical stimulation cycles, the retained current progressively increases relative to the initial state, leading to an increase in the retained current change (Δ*I*). Here, Δ*I* is defined with respect to the initial current state, and therefore progressively increases over the early conditioning cycles before approaching saturation. The corresponding current values are provided in Table S6. This behavior indicates that the current state (*I*) established by the preceding stimulation is partially retained and influences the starting state of the subsequent learning cycle. As a result, repeated optoelectrical stimulation progressively modifies the device state, demonstrating the stimulation history‐dependent behavior described here as optoelectrical conditioning. During the later cycles, the smaller change in the retained current indicates that the device approaches a relatively stable conditioned state. Figure 4d further demonstrates the reproducibility of this phenomenon, showing similar optoelectrical conditioning characteristics across the same current range. To comprehensively compare the electrical and optoelectrical learning and forgetting processes, we analyzed the retained current after each consecutive potentiation and depression cycle. The comparative histogram (Figure 4e) reveals that under electrical stimulation, the maximum current remains relatively constant across cycles, whereas in the optoelectrical mode, it increases over the first three cycles before reaching saturation. This nonlinear increment in current under optical stimulation, unlike the linear response typically seen in photodiodes, is a critical factor for neuromorphic computing. Figure 4f compares the retained current between the two different devices after successive potentiation and depression cycles, further confirming the consistency of this behavior. Retained current increases for the first three learning cycles and approached a stable state. Here, Δ*I*_*n*_ = *I*_retain_,_*n*_ − *I*_initial_, where *I*_retain_,_*n*_ is the retained current after the “*n*”th potentiation–depression cycle and *I*_initial_ is the initial current before conditioning. To evaluate the learning and forgetting dynamics of the Ag/GQD–VO_2_/Ni device, we extracted the time constant (*τ*) values by fitting the potentiation and depression responses. The analysis was performed for the first three cycles of potentiation and depression, representing the initial optoelectrical conditioning region, and for the fourth cycle, representing the after optoelectrical conditioning region, as shown in Figure S18. The extracted *τ* values are summarized in Table S5. For LTP, the initial *τ* value (1.5 ms) was relatively low, indicating rapid conductance modulation during the early learning stage. As the LTP cycles progressed, *τ* gradually increased, reaching 4.5 ms after optoelectrical conditioning. This increase in *τ* can be associated with the influence of previous stimulation, resulting in progressively smaller conductance updates as the synaptic state approached saturation. This increase in *τ* reflects photocarrier trapping and gradual conductive filament stabilization during repeated optoelectrical stimulation. Similarly, LTD exhibited an initial *τ* of 1.0 ms, which increased progressively with subsequent cycles, reaching 3.8 ms after optoelectrical conditioning, indicating that forgetting dynamics also became more gradual during optoelectrical conditioning due to the partial relaxation of the conductive pathways during depression. These results demonstrate that repeated optoelectrical stimulation modifies the potentiation–depression dynamics. The increase in fitted time constants, together with the evolution of the retained conductance, characterizes the progression of optoelectrical conditioning. The nonlinearity analysis further shows improved potentiation–depression linearity in the fourth cycle compared with that in the first cycle (Figure S19 and Table S5). Notably, the device exhibits significantly improved linearity after conditioning.

**FIGURE 4:**
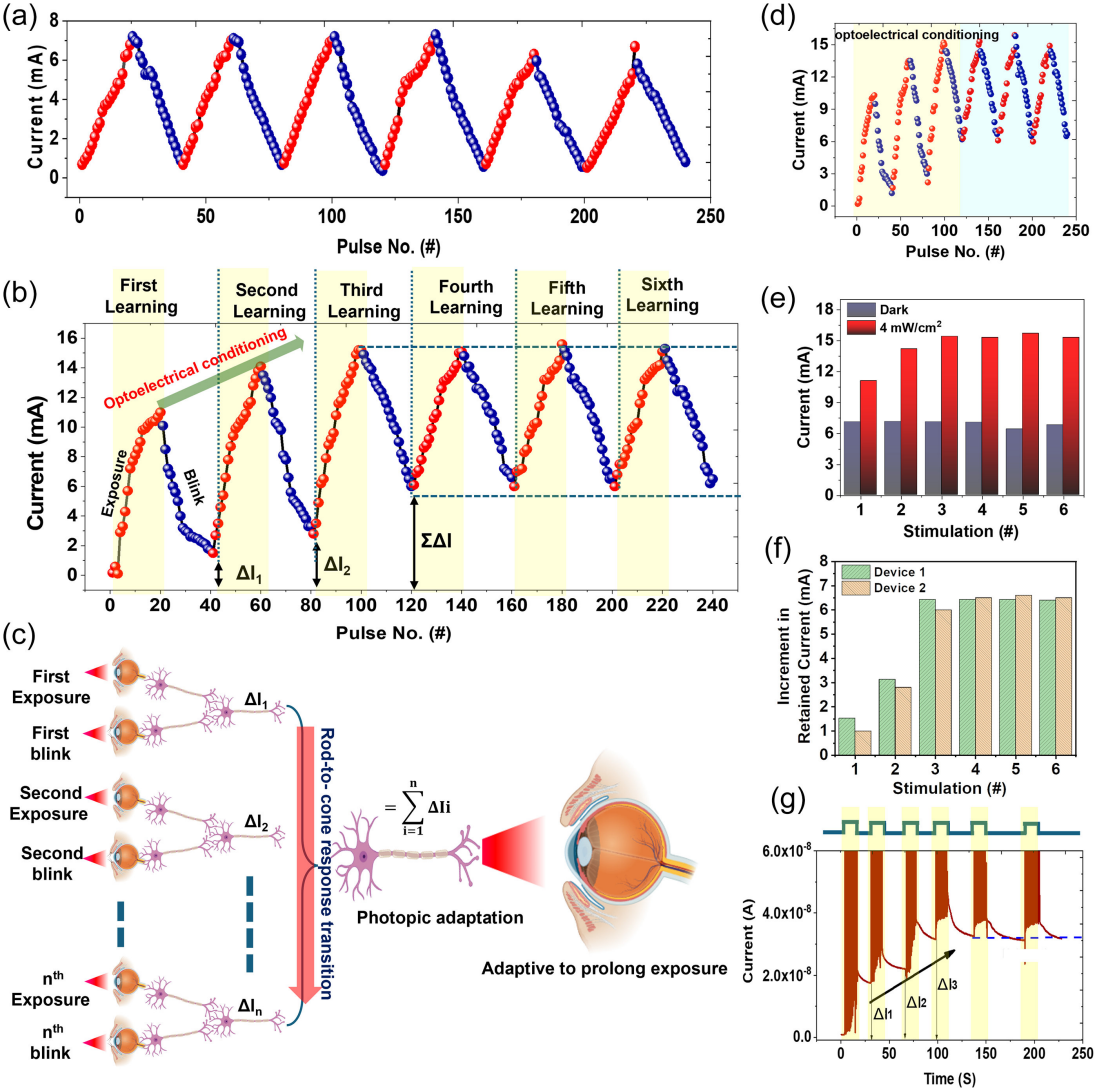
(a) Consecutive six cycles of electrical potentiation and depression in Ag/GQD–VO_2_/Ni device under dark conditions. (b) Consecutive six cycles of optoelectrical potentiation and depression in the Ag/GQD–VO_2_/Ni device under 4 mW/cm^2^ illumination. ΣΔ*I*: cumulative current increase after the third cycle. (c) Schematic of photopic adaptation. (d) Repetitive potentiation and depression cycles of Ag/GQD–VO_2_/Ni device. (e) Comparative current level analysis of six cycles under dark conditions and 4 mW/cm^2^ illumination. (f) Retained‐current changes relative to the initial state across successive learning cycles in two randomly selected devices under 4 mW/cm^2^ illumination. (g) Postsynaptic current is measured by applying a series of 30 consecutive optical pulses at 405 nm (4 mW/cm^2^) with a 30 s interval between each cycle.

To implement this optoelectrical conditioning phenomenon, retention of the conductance states before and after conditioning is crucial. Accordingly, we measured the retention characteristics of the Ag/GQD–VO_2_/Ni device both prior to and following optoelectrical conditioning adaptation for 500 s under a read voltage of 0.02 V and a set voltage of 0.8 V, as shown in Figure S20. Furthermore, to confirm the optoelectrical conditioning behavior of the device in the purely optical mode, we applied a series of 30 consecutive optical pulses at 405 nm (4 mW/cm^2^) at 30 s intervals. The results are shown in Figure 4g. Similar to the optoelectronic mode, we observed that after the first cycle of 30 consecutive optical pulses, the device retained a measurable current, suggesting memory‐like behavior. The retained current increased consistently for up to three cycles, indicating a progressive change in the conductance state. However, after the third cycle, the additional conductance response to successive optical stimulation decreased, suggesting that the device approached a relatively stable updated state.

### 2.3 Learning Capability in the Ag/GQD–VO_2_/Ni Device Under Optical Stimulation

The human visual system forms specific memory associations with light wavelengths after adapting to different intensities. For associative learning, exposure to different wavelengths significantly influences learning ability and memory formation. Therefore, we examined the retained memory of the device with varying numbers of optical pulses. Figure 5a illustrates the postsynaptic response of the device under a 405 nm laser pulse at a frequency of 5 Hz for 3, 20, and 50 pulses. When subjected to only three pulses, the device rapidly returned to its original conductance state, emulating short‐term memory (STM). After 20 pulses, a moderate level of conductance retention was observed, indicating mid‐term memory (MTM). However, upon applying 50 pulses, the device exhibited significantly higher retained memory that remained stable for up to 40 s after stimulus termination, demonstrating long‐term memory (LTM) characteristics. We observed the non‐monotonic behavior attributed to the stochastic trapping and detrapping of photogenerated carriers associated with defect states and interfacial traps within the GQD–VO_2_ heterostructure. Furthermore, cycle‐to‐cycle variability measurements performed using 20 pulses (pulse width 0.5 s) indicate that the MTM reveals similar non‐monotonic responses, suggesting that this behavior is intrinsic (Figure S21). Overall, these results demonstrate STM, MTM, and LTM behaviors, depending on the number of optical pulses applied, demonstrating their suitability for artificial synaptic applications.

**FIGURE 5:**
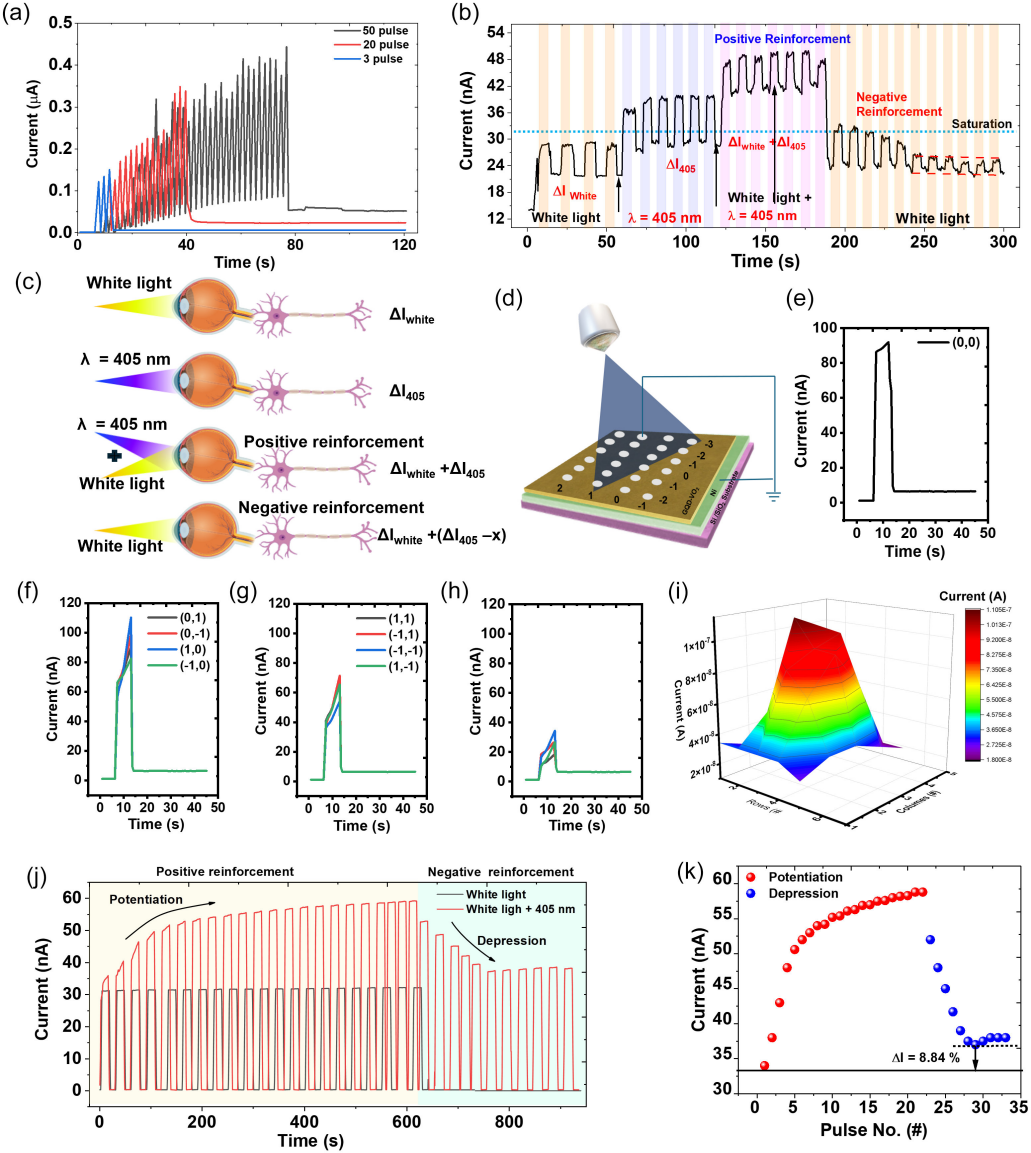
(a) Ag/GQD–VO_2_/Ni device demonstrates optical short‐term, mid‐term, and long‐term memory under 405 nm laser illumination, corresponding to 3, 20, and 50 pulses, respectively. (b) Associative learning behavior of the Ag/GQD–VO_2_/Ni device under 405 nm and white light illumination at 0.5 Hz frequency, demonstrating positive and negative reinforcements. The synaptic response is enhanced when 405 nm and white light are applied together, indicating associative strengthening. (c) Schematic of associative learning in human eyes with white light and 405 nm laser. (d) Schematic of fabricated Ag/GQD–VO_2_/Ni devices under 405 nm laser illumination. Optical pulse response of Ag/GQD–VO_2_/Ni device at (e) (0, 0) position, (f) (0, 1), (0, −1), and (1, 0) position, (g) (1, 1), (−1, 1), (1, −1), and (1, −1) position, and (h) remaining outside positions. (i) 3D representation of the current response at different positions. (j) Potentiation and depression were evaluated under two conditions: exposure to white light alone and a combination of white light with 405 nm laser pulses (4 mW/cm^2^). (k) Potentiation and depression in the form of current values from positive reinforcement and negative reinforcement.

Figure 5b shows associative learning results with sequential light stimuli. The device was first exposed to white light, followed by 405 nm laser pulses (4 mW/cm^2^), a combination of white and blue lights, and finally, only white light with a frequency of approximately 0.5 Hz. The blue dotted line represents the threshold current (2.9 × 10^−8^ A) above which associative behavior is defined. In biological vision systems, exposure to white light induces a memory effect, denoted as Δ*I*_white_, whereas exposure to blue light (405 nm) creates a distinct memory, denoted as Δ*I*_405_ [64, 65]. When the device was subjected to white and blue lights in combination, an enhanced synaptic response was observed due to the reinforcement of previously formed wavelength‐specific memories, an effect known as associative learning [66]. Upon termination of blue light during continuous white light exposure, the device demonstrated further memory consolidation, indicating its ability to reinforce learned responses over time. A similar phenomenon was reported by Kim et al. [67], in which individuals exposed to blue light for a certain period (approximately 20 min) exhibited enhanced long‐term learning abilities compared to those exposed to other light environments. This memory retention effect after blue light exposure arises from the combined effect of Δ*I*_white_ and the residual memory of blue light (Δ*I*_405−*x*_) (Figure 5c) [66, 68].

Our device exhibited a comparable response, where white light exposure induced Δ*I*_white_, 405 nm laser pulses increased the current response, and their combination increased synaptic behavior. This positive reinforcement resulted in a higher response than that observed for the other stimuli. Upon cessation of blue light, the current exhibited a slight decrease, indicating negative reinforcement. However, even after five pulses, the retained conductance was slightly higher compared to exposure to only white light, suggesting the cumulative effect of Δ*I*_white_ and the residual component of Δ*I*_405−*x*_. This type of associative learning demonstrates the potential of neuromorphic devices to emulate complex biological learning mechanisms [66, 69]. The same phenomenon is further examined by ceasing white‐light exposure to simulate negative reinforcement (Figure S22). To rule out nonassociative learning, we performed control experiments using white light and a 1550 nm laser, and the results are presented in Figure S23. The device exhibited a clear response to white light but showed no response to the 1550 nm laser. Furthermore, the combined stimulation of white light and 1550 nm laser produced a response identical to that of white light alone, indicating the absence of both positive and negative reinforcement with 1550 nm input. This observation confirms that the device behavior is nonassociative.

To examine the spatial optical response, we exposed the fabricated devices to a constant laser source at a fixed position, with labeled device locations, as depicted in Figure 5d. The laser‐power‐dependent response of a single optical pulse is shown in Figure S24. Figure 5e shows the optical response of the central (0, 0) device, whereas Figure 5f shows the responses of the devices positioned at (0, 1), (0, −1), (1, 0), and (−1, 0). Figure 5g shows the responses at positions (1, 1), (−1, 1), (−1, −1), and (1, −1), whereas Figure 5h shows the responses of the remaining outer devices. Figure 5i shows a 3D representation of collective current response across various device positions, illustrating the spatial distribution of synaptic behavior under optical stimulation.

This positive and negative reinforcement property of the device was further utilized to investigate its impact on synaptic potentiation and depression. Potentiation was evaluated under two conditions: exposure to white light alone and a combination of white light with 405 nm laser pulses (4 mW/cm^2^), as shown in Figure 5j. The device exhibited no significant increase in conductance under white‐light stimulation alone. In contrast, the presence of positive reinforcement from the 405 nm laser led to a pronounced increase in current with successive pulses. Upon cessation of the 405 nm stimulus, the device demonstrated a decrease in conductance, indicating synaptic depression. Typically, in optoelectronic devices, optical stimulation induces potentiation, while electrical input is required for depression [70]. Uniquely, in our case, both potentiation and depression were achieved solely in the optical domain through positive and negative reinforcement. The corresponding current values for potentiation and depression are presented in Figure 5k, suggesting memory modulation through the reinforcement process. A maximum increase of up to 8.84% in synaptic weight was observed, confirming memory consolidation associated with the 405 nm laser stimulus.

Optical stimulation‐based engineered devices with potential applications as artificial electronic synapses were characterized and evaluated using image classification tasks across each training epoch. Moreover, the 405 nm laser‐controlled Ag/GQD–VO_2_/Ni device varied the slope and dynamic range of the potentiation and depression curves, key parameters for determining the fidelity of artificial neural network (ANN) computations in neuromorphic computing systems [10, 71]. The datasets, Fashion‐MNIST and Digit‐MNIST, were used to investigate the functioning of the synaptic properties of the Ag/GQD–VO_2_/Ni device at two different stages, before and after optoelectrical conditioning [72, 73]. ANN approaches for image pattern recognition are presented in Figure 6a. The ANN structure consists of an input layer of 784 neurons (28 × 28 pixels), a 200‐neuron hidden layer, and an output layer of 10 neurons for classification. The MNIST and Fashion‐MNIST datasets were used for training and evaluation, consisting of 60 000 training samples and 10 000 test samples. The training process was conducted over 40 epochs using a batch size of 1024. The synaptic weights are mapped to experimentally measured conductance values. Figure 6b shows the normalized conductance of potentiation and depression in the optoelectrical mode during the first cycle, referred to as before optoelectrical conditioning. Figure 6c shows the normalized potentiation and depression conductance for the fourth cycle of the optoelectrical mode, after optoelectrical conditioning. The fitted potentiation and depression curves show nonlinearity coefficients for LTP (*ν* ^+^) and LTD (*ν* ^−^) of 0.1 and 0.18 before optoelectrical conditioning and 0.03 and 0.02 (Table S5) after optoelectrical conditioning, respectively. An ideal coefficient is zero, representing perfectly linear potentiation and depression curves. The nonlinear coefficients are calculated using Equations (1) and (2), based on the normalized potentiation and depression curves.

**FIGURE 6:**
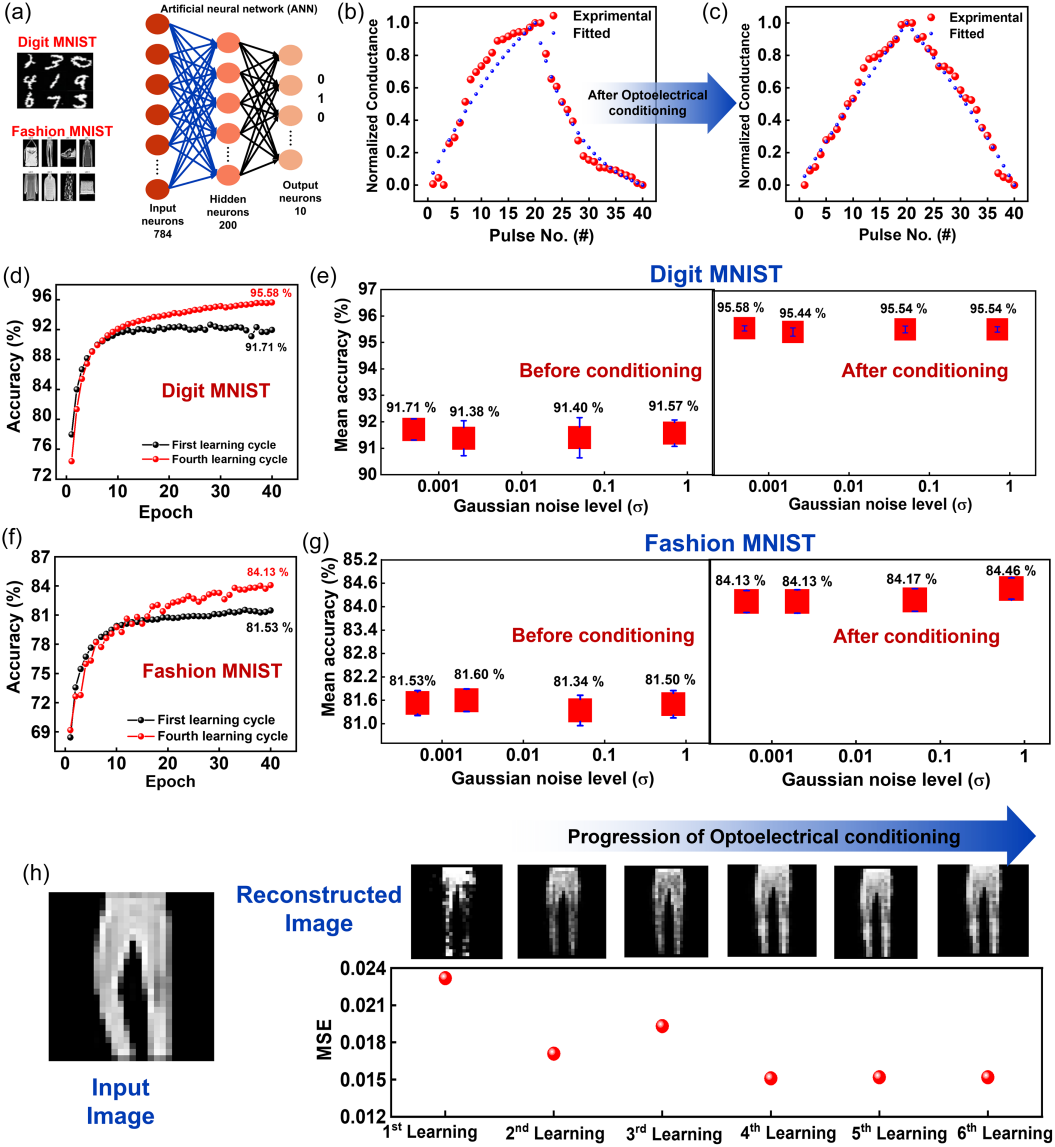
(a) Image pattern recognition by Fashion‐MNIST and Digit‐MNIST via the artificial neural network approach. (b) Normalized conductance during potentiation and depression in the optoelectrical mode for the first cycle, referred to as the state before optoelectrical conditioning. (c) Normalized conductance during potentiation and depression in the optoelectrical mode for the fourth cycle, referred to as the state after optoelectrical conditioning. (d) Classification accuracy for the Digit‐MNIST dataset before and after optoelectrical conditioning. (e) Monte Carlo (MC) simulation results depicting the mean classification accuracy with the corresponding standard deviation (SD) for the Digit‐MNIST dataset. (f) Classification accuracy for the Fashion‐MNIST dataset before and after optoelectrical conditioning. (g) MC simulation results depicting the mean classification accuracy with the corresponding standard deviation (SD) for the Fashion‐MNIST dataset. (h) Image pattern recognition and reconstruction of Fashion‐MNIST for the six learning processes obtained from Figure 4b.

(1) $$G^{+}=G_{\min}^{+}+\frac{G_{\max}^{+}-G_{\min}^{+}}{1-e^{-v^{+}\cdot P_{\max}}}\cdot \left(1-e^{-v^{+}\cdot P}\right)$$

(2) $$G^{-}{=G}_{\min}^{-}+\frac{G_{\max}^{-}-G_{\min}^{-}}{1-e^{-v^{-}\cdot P_{\max}}}\cdot \left(1-e^{v^{-}\cdot \left(P-P_{\max}\right)}\right)$$

where *G* ^+^ and *G* ^−^ are the normalized conductances of potentiation and depression, respectively; *ν* ^+^ and *ν* ^−^ are the nonlinearity coefficients of potentiation and depression, respectively; *P*_max_ is the maximum pulse number. To address the negative weight values, we adopted a differential method using two devices. The ANN weight (*w*) was defined as *w* = *G* ^+^ + *G* ^−^ − 0.1. To update weights with the potentiation or depression conductance, we applied a single pulse to the device for power efficiency, ensuring that the conductance changes in the potentiation and depression were unidirectional. Furthermore, a reset mechanism was applied to stabilize the training process when the conductance reached the final stage of potentiation and depression. The results indicate that for the Digit‐MNIST dataset, a classification accuracy of 91.71% is achieved before conditioning, which improved to 95.58% after conditioning (Figure 6d). Similarly, for the Fashion‐MNIST dataset, accuracy increases from 81.53% before conditioning to 84.13% after conditioning (Figure 6f). These findings suggest that the evolution of the synaptic characteristics during optoelectrical conditioning contributes to improved pattern recognition performance, demonstrating the potential of the proposed device for neuromorphic computing applications. To further validate robustness against stochastic variability, MC simulations were performed by introducing Gaussian noise into the modeled conductance states at different noise levels (*σ* =  0.0005, 0.002, 0.05, and 0.7) to emulate variability [74, 75, 76]. The results are reported as mean accuracy  ±  SD across multiple MC runs. Here, *σ* denotes the Gaussian noise introduced in the simulation, while SD represents the run‐to‐run variation across independent trials (Figure S25). For Digit‐MNIST, the mean accuracy before conditioning is 91.71% ± 0.40%, 91.38% ± 0.66%, 91.40% ± 0.76%, and 91.57% ± 0.50%, across increasing *σ* values. After conditioning, the corresponding accuracies improve to 95.58% ± 0.10%, 95.44% ± 0.16%, 95.54% ± 0.13%, and 95.54% ± 0.10% for increasing *σ*, illustrated in Figure 6e. Similarly, for the Fashion‐MNIST dataset, the accuracy before conditioning is 81.53% ± 0.32%, 81.60% ± 0.29%, 81.34% ± 0.39%, and 81.50% ± 0.35%, whereas after conditioning, it increases to 84.13% ± 0.28%, 84.13% ± 0.30%, 84.17% ± 0.29%, and 84.46% ± 0.27% illustrated in Figure 6g. Importantly, across all noise levels, the accuracy after conditioning is higher than before conditioning and consistent with run‐to‐run variations (SD), indicating that improvement is not due to random fluctuations. Across the evaluated simulation settings, the conditioned‐state characteristics consistently yielded higher classification accuracy than the initial‐state characteristics. Furthermore, in order to examine the effect of conditioning behavior of the repeated learning cycles on learning efficiency, the Fashion‐MNIST dataset was employed to train ANN model using the experimentally measured LTP/LTD conductance (Figure 4b) values from each learning cycle as synaptic weight update rules. The reconstructed image outputs and mean squared error (MSE) reconstruction loss were analyzed across all six learning cycles, as shown in Figure 6h. The MSE reconstruction loss progressively decreased from 0.0231 (first learning cycle) to 0.0171 (second learning cycle), accompanied by visually improved reconstructed images, confirming progressive learning behavior (conditioning region). The slight variation observed in the third learning cycle is attributed to natural device conductance variation. In contrast, the fourth, fifth and sixth learning cycles exhibit nearly identical MSE reconstruction loss values of ~0.0151, with visually stable reconstructed images, confirming the onset of conditioning behavior. This stabilized reconstruction quality across repeated learning cycles suggests that the device reaches an adaptive steady state, analogous to biological conditioning. To further validate the reconstruction performance, peak signal‐to‐noise ratio (PSNR) and structural similarity index measure (SSIM) were evaluated alongside the MSE reconstruction loss for each learning cycle, as provided in Figure S26. The classification accuracy of each learning cycle is provided in Figure S27. The study of the present work with previously reported optoelectronic devices based on memristors and transistors is summarized in Tables S2 and S7. The comparison highlights that the fabricated device exhibits multifunctionality, including cognitive functions such as optoelectrical conditioning (optically and optoelectrical) along with associative learning, as well as positive and negative reinforcement, demonstrating its potential for future optoelectronic synaptic applications.

## 3 Conclusion

In this study, we demonstrated a 0D/3D Ag/GQD–VO_2_/Ni optoelectronic synaptic device capable of integrating optical sensing, memory storage, and adaptive synaptic behavior with a simple two‐terminal architecture. Compared with the bare VO_2_ device, the engineered GQD–VO_2_ heterostructure broadened the dynamic response from 2.06 to 44.29, enhanced the photonic responsivity by ~fourfold (from 0.07 to 0.29 A/W), lowered the optoelectronic energy consumption by 86% (from 220 to 31.5 nJ per spike), and improved the analog linearity (*R* ^2^: 0.81–0.97). We also observed optoelectrical conditioning under repeated stimulation, characterized by a progressive increase in the retained current, increasing learning and forgetting time constants (*τ*), and improved synaptic linearity. In addition, wavelength‐dependent optical associative learning together with positive and negative reinforcement behaviors was demonstrated. The influence of optoelectrical conditioning on learning performance was further evaluated through image‐recognition accuracy, which increased from 81.53% to 84.13% for Fashion‐MNIST and from 91.71% to 95.58% for Digit‐MNIST, supported by MC simulations. Furthermore, the progression of optoelectrical conditioning was reflected in the evolution and subsequent stabilization of the reconstructed Fashion‐MNIST images across repeated learning cycles. These findings demonstrate the potential of the proposed GQD–VO_2_ heterojunction for optoelectrical conditioning, highlighting the potential for neuromorphic computing applications.

## 4 Experimental Section

### 4.1 Atomic Layer Deposition of VO_2_ on Ni

Amorphous vanadium oxide thin films were grown with a target thickness of 30 nm via atomic layer deposition (ALD) in a traveling wave‐type 4‐inch chamber (Atomic‐Class, CN‐1, Korea) using vanadium (V) oxytriisopropoxide (VTIP, Sigma‐Aldrich) as the vanadium source and deionized (DI) water as the O_2_ source. VTIP and DI water canisters were kept at 45 °C and room temperature, respectively. Pure N_2_ (99.999%) was used as carrier and purge gases at a flow rate of 200 sccm. The deposition temperature was set to 150 °C. The ALD cycle comprised a 1.5 s VTIP pulse for 15 s of purging, 5 s of H_2_O pulse, and 10 s of purging. The as‐deposited films were then annealed at 500 °C for 1 h in a forming gas (95% N_2_ + 5% H_2_) atmosphere in a box furnace (C‐A14P, Hantech, Korea) to achieve the VO_2_ phase. ALD offers potential for large‐area VO_2_ deposition. However, wafer‐scale fabrication would require optimization of the annealing conditions and consideration of compatibility with other device layers.

### 4.2 Fabrication of the Ag/GQD–VO_2_/Ni Device

The fabrication process for the Ag/GQD–VO_2_/Ni device is illustrated in Figure S1a. Initially, a Ni bottom electrode was deposited on the substrate using electron beam (e‐beam) evaporation. Next, a VO_2_ thin film was deposited using ALD to serve as the active layer. Following the VO_2_ deposition, graphene QDs (GQDs, Sigma‐Aldrich; aqueous dispersion with a concentration of >0.005 wt%) were drop‐cast (20 μL) onto the VO_2_ layer and dried in a vacuum oven at 50 °C for 20 min to ensure uniform deposition. For large‐area fabrication, GQD drop casting may require further optimization to maintain uniform thickness and coverage. Future work would investigate controlled spin‐coating or printing as alternative deposition methods. Finally, a Ag top electrode was deposited via the e‐beam evaporation technique using a shadow mask of 300 μm electrode diameter to define the pattern. A 99.9% pure Ag metal target, sourced from LTS Research Laboratories (USA), was used for deposition. The fabricated Ag/GQD–VO_2_/Ni device was subjected to electrical and optical measurements to evaluate its neuromorphic and optoelectronic properties.

## Author Contributions

P.J. and S.S.K. prepared the device for most of the experimental measurements, also measured the electrical and optical characteristics, and analyzed the results. Y.T.K. and S.W.L. prepared the materials. S.S.K., P.J., and J.H.P. conceived and designed the study. W.A.L., Y.T.M., and S.W.L. assisted with physicochemical characterization and optical instrumentation. S.S.K., P.J., J.H., and J.H.P. prepared the manuscript. All authors discussed the results and commented on the manuscript. All authors revised and commented on the manuscript. J.H., and J.H.P. supervised this study.

## Funding

This study was supported by the National Research Foundation of Korea (NRF) grants funded by the Government of the Republic of Korea (RS‐2023‐00275300, 2022R1A2C2006532, and RS‐2026‐25468512), Learning & Academic Research Institution for Master’s·PhD students, and Postdocs (LAMP) Program of the National Research Foundation of Korea (NRF) grant funded by the Ministry of Education (No. RS‐2023‐00301974).

## Conflicts of Interest

The authors declare no conflicts of interest.

## Supporting information

Supplementary Material

## Data Availability

The data supporting these plots and other findings are available from the corresponding author upon request.
