# Supplementary material for: Optoelectrical Conditioning in Graphene Quantum Dot‍–‍Vanadium Oxide Artificial Synapse
Source: Small Sci. 2026 Oct 27;6(11):e70434. doi: 10.1002/smsc.70434 (PMC13713603; doi:10.1002/smsc.70434)
Supplement: Supplementary file 1 [file SMSC-6-e70434-s001.pdf]

Supporting Information

Optoelectrical Conditioning in Graphene Quantum Dot-VO_2 Artificial Synapse

Prabana Jetty[1†], *Somnath S. Kundale*[1,2 †], *Yong Tae Kim*[3†],*Windy Ayu Lestari* [1†], *Sang Won Lee*[3], *Jaeyeong Heo*[3]*, *Jun Hong Park*[1]*

P.Jetty, S. S. Kundale, , W. A. Lestari, J. H. Park

[1] Department of Materials Engineering and Convergence Technology, Gyeongsang National University, Jinju, Gyeongsangnam-do 52828, Republic of Korea

S. S. Kundale

[2] Research Institute for Green Energy Convergence Technology, Gyeongsang National University, Jinju 52828, Republic of Korea

Y. T. Kim, S. W. Lee, J. Heo

[3] Department of Materials Science and Engineering, and Optoelectronics Convergence Research Center, Chonnam National University, Gwangju 61186, Republic of Korea

*Corresponding author

Email: jheo@jnu.ac.kr

Email: yakte@gnu.ac.kr

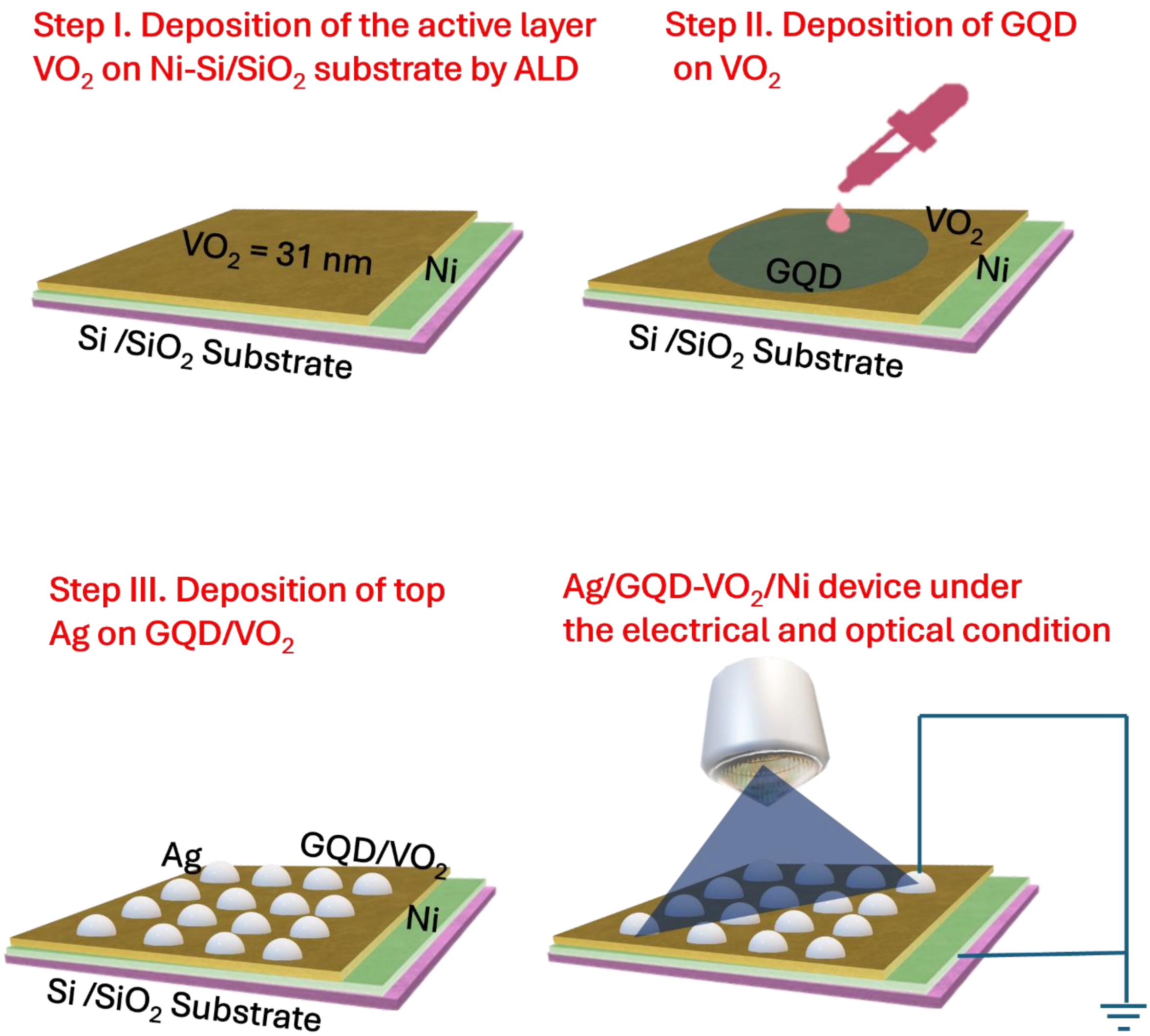

Figure S1. Schematic of the fabrication process of the Ag/GQD-VO_2/Ni device.

Figure S1. presents a schematic illustration of the stepwise fabrication process of the Ag/GQD–VO_2/Ni device, as described in detail in the Experimental Section. The figure also includes the configuration of the device under optoelectrical measurement conditions.

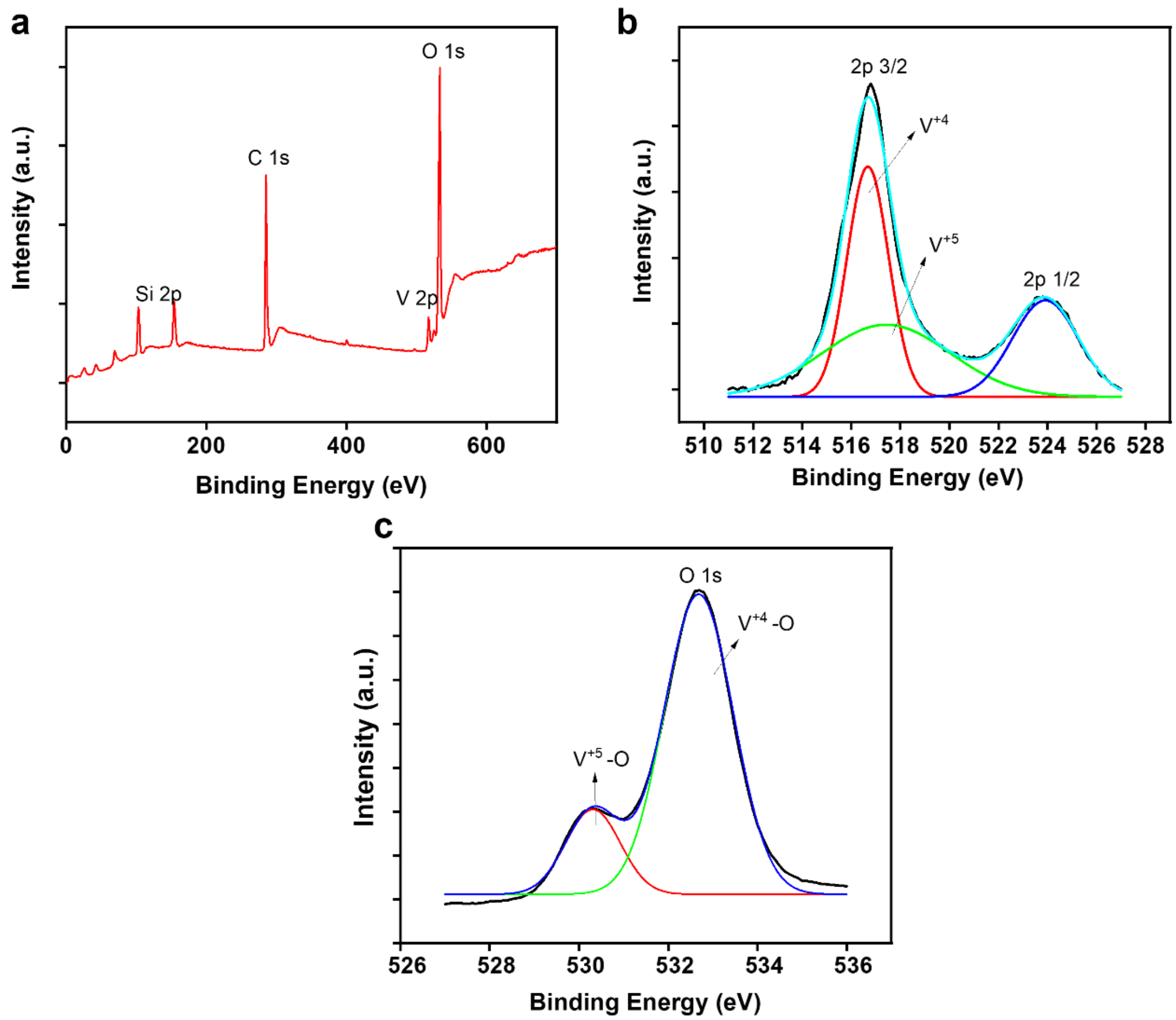

Figure S2. XPS analysis of VO_2 (a) survey spectra of VO_2, (b) deconvolution of the V 2p peak, and (c) deconvolution of the O 1s peak.

To confirm the elemental composition and phase of vanadium oxide, X-ray photoelectron spectroscopy (XPS) analysis was performed. **Figure S2a** presents the survey spectrum of the VO_2 film, which exhibits characteristic peaks corresponding to V 2p and O 1s, associated with vanadium oxide. The high-resolution XPS core-level spectrum of V 2p was deconvoluted into multiple peaks corresponding to different oxidation states of vanadium, ensuring that the binding energy separation between V $2p_{3/2}$ and V $2p_{1/2}$ remains within 7 eV. [1, 2] The deconvoluted XPS spectra of V 2p and O 1s for the VO_2 film are shown in **Figure S2b and c**, respectively. As

illustrated in **Figure S2b**, the deconvolution of the V 2p core-level spectrum reveals four distinct peaks. The peaks at 517 eV and 515.5 eV correspond to V^{5+} $2p_{3/2}$ and V^{4+} $2p_{3/2}$, respectively. [2, 3] The relative concentration of V^{5+} and V^{4+} oxidation states, estimated from the ratio of their integrated intensities, indicates the VO_2 phase is dominant than the V_2O_{5-x}. It confirms the VO_2 formation.

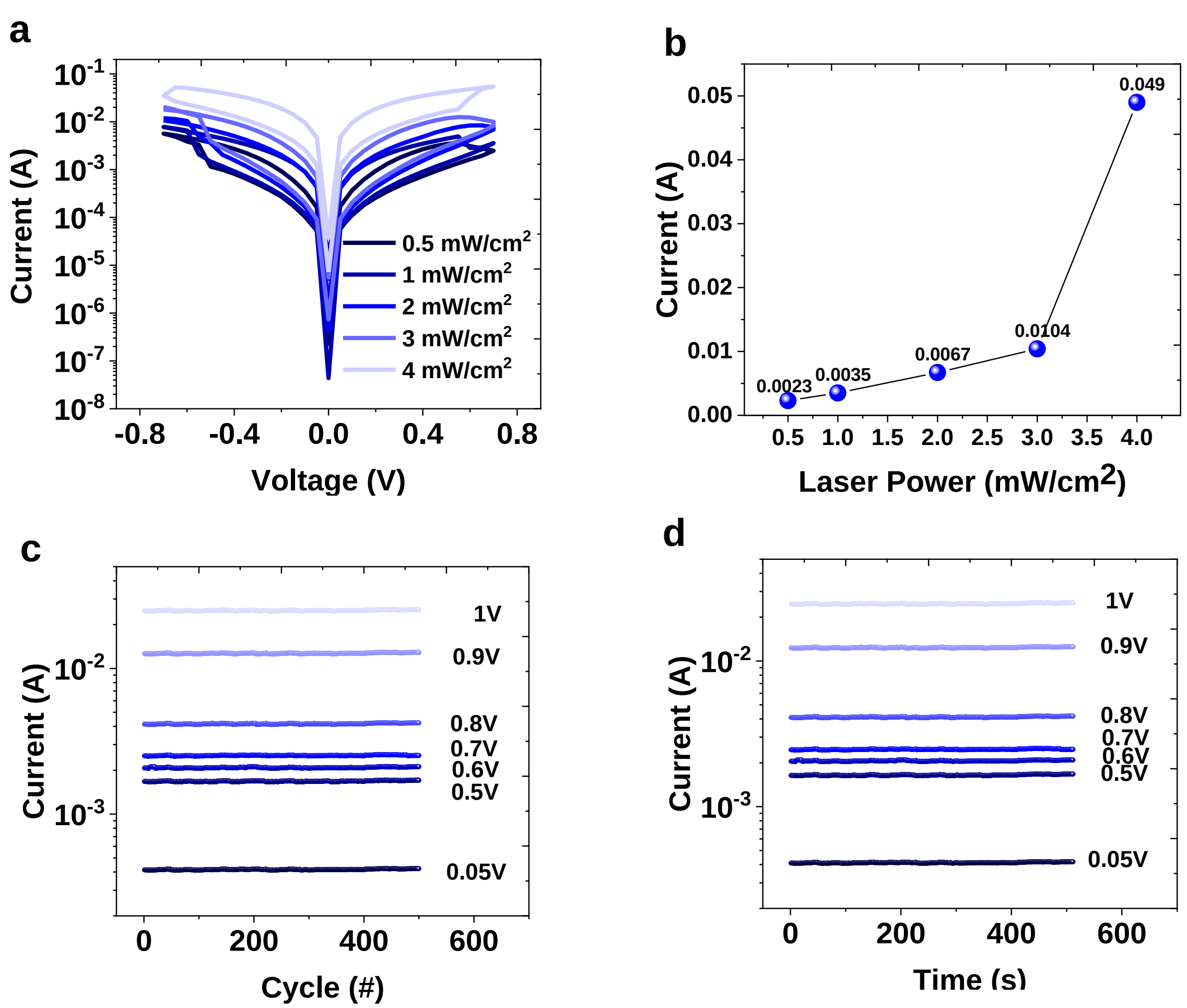

Figure S3. (a) I-V characteristics of the Ag/VO_2/Ni device under a 405 nm wavelength laser with varying power intensities. (b) Laser power-dependent increment in current in the Ag/VO_2/Ni device. (c) Endurance of the Ag/VO_2/Ni device. (d) Retention of the Ag/VO_2/Ni device.

Due to the visible range absorbance of VO_2, modifications in the electrical characteristics are observed. To explore this, we measured the I-V characteristics of the Ag/VO_2/Ni device under a 405 nm wavelength laser with varying power intensities, as shown in **Figure S3a**. The results reveal that the device is photoactive, with a significant impact on its I-V characteristics. Specifically, the current levels increase proportionally with the laser power, ranging from 0.5 to 4 mW/cm^2 as shown in **Figure S3b**. Multilevel conductance, a crucial feature for memory devices in neuromorphic applications, was further investigated. Endurance and retention measurements were performed at different voltages under 0.5 mW laser power, as presented in **Figure S3c and d**, respectively.

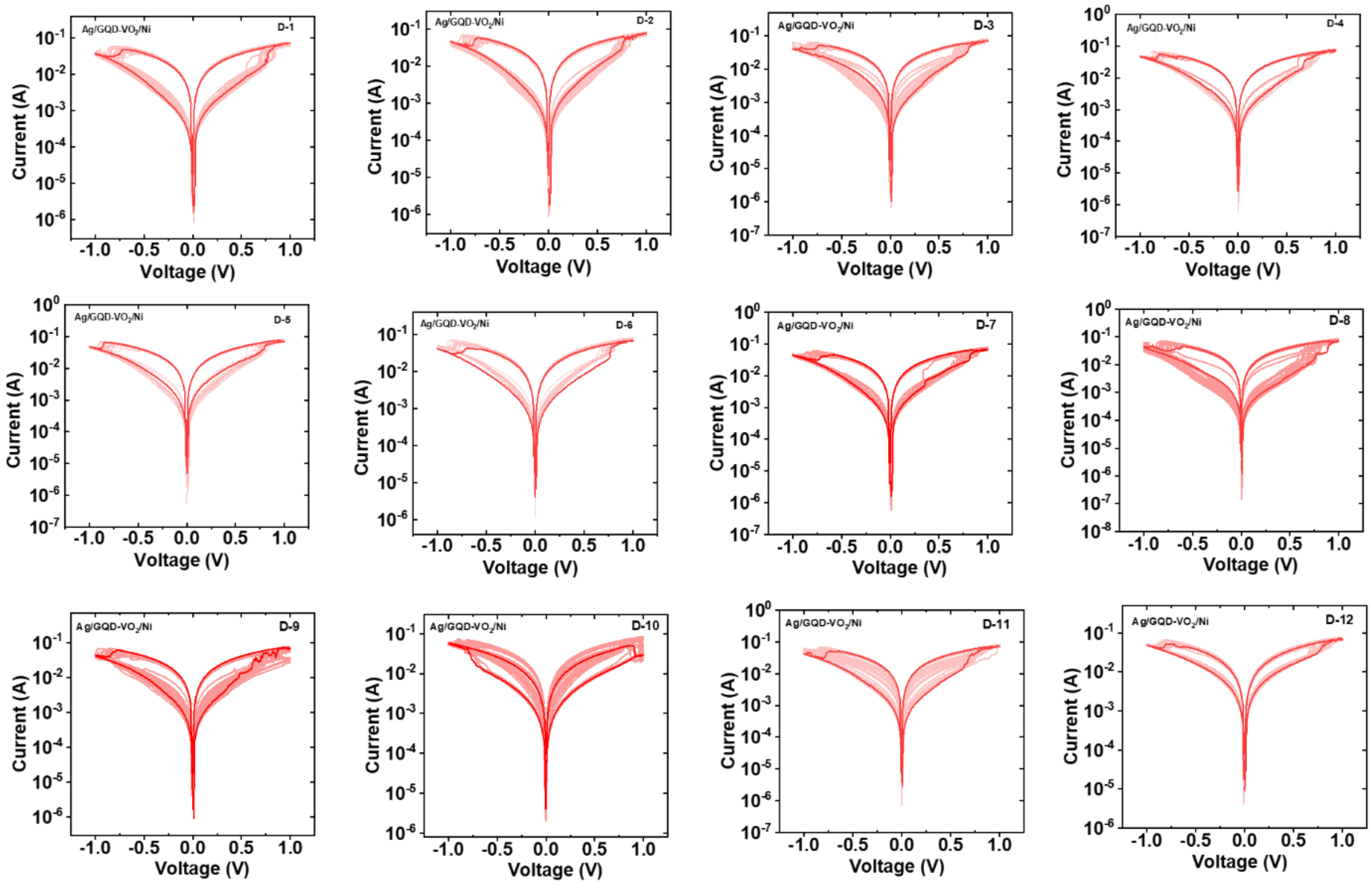

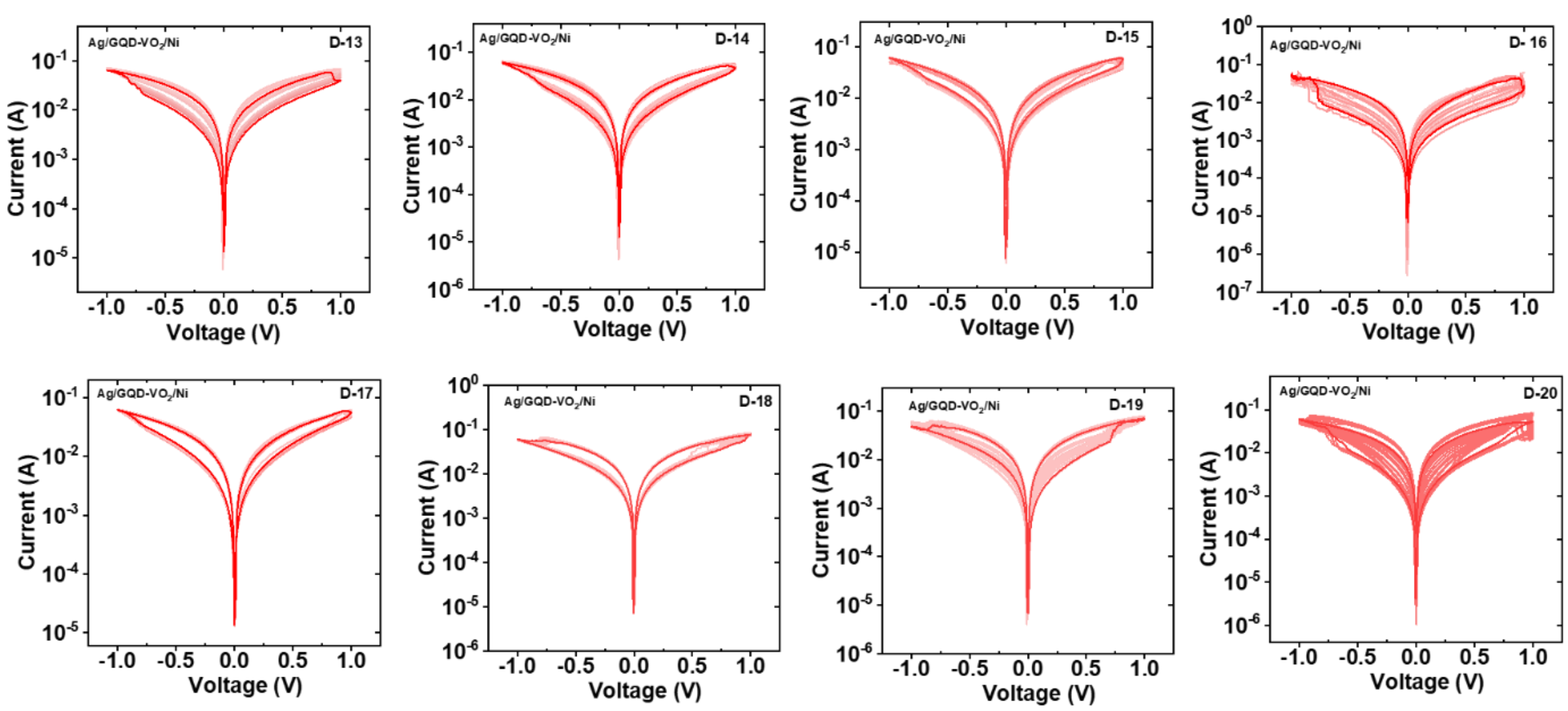

Figure S4. Device-to-Device and cycle-to-cycle I-V characteristics of Ag/GQD-VO_2/Ni devices measured across 20 devices, with 20 consecutive cycles per device.

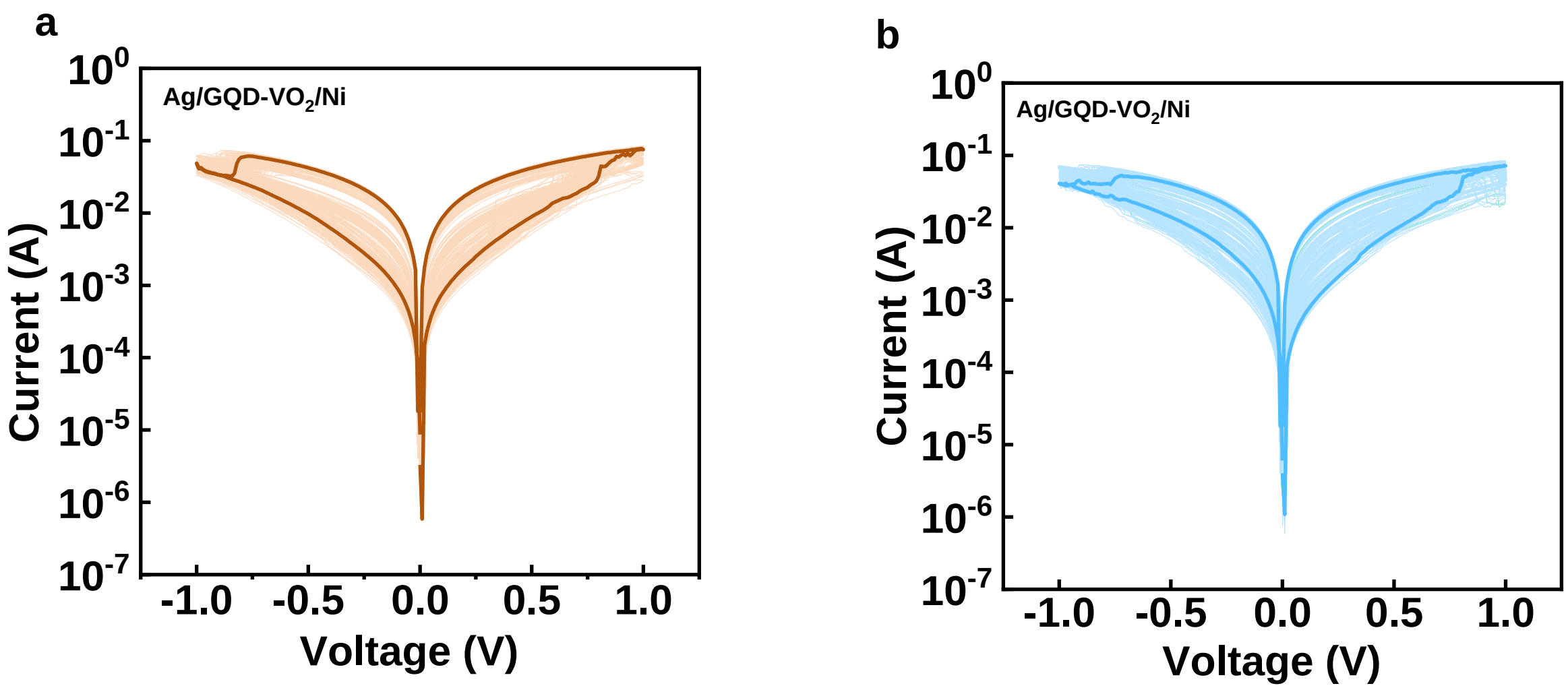

Figure S5. Cycle-to-cycle variability was evaluated by repeating the measurement over 250 consecutive cycles for each device.

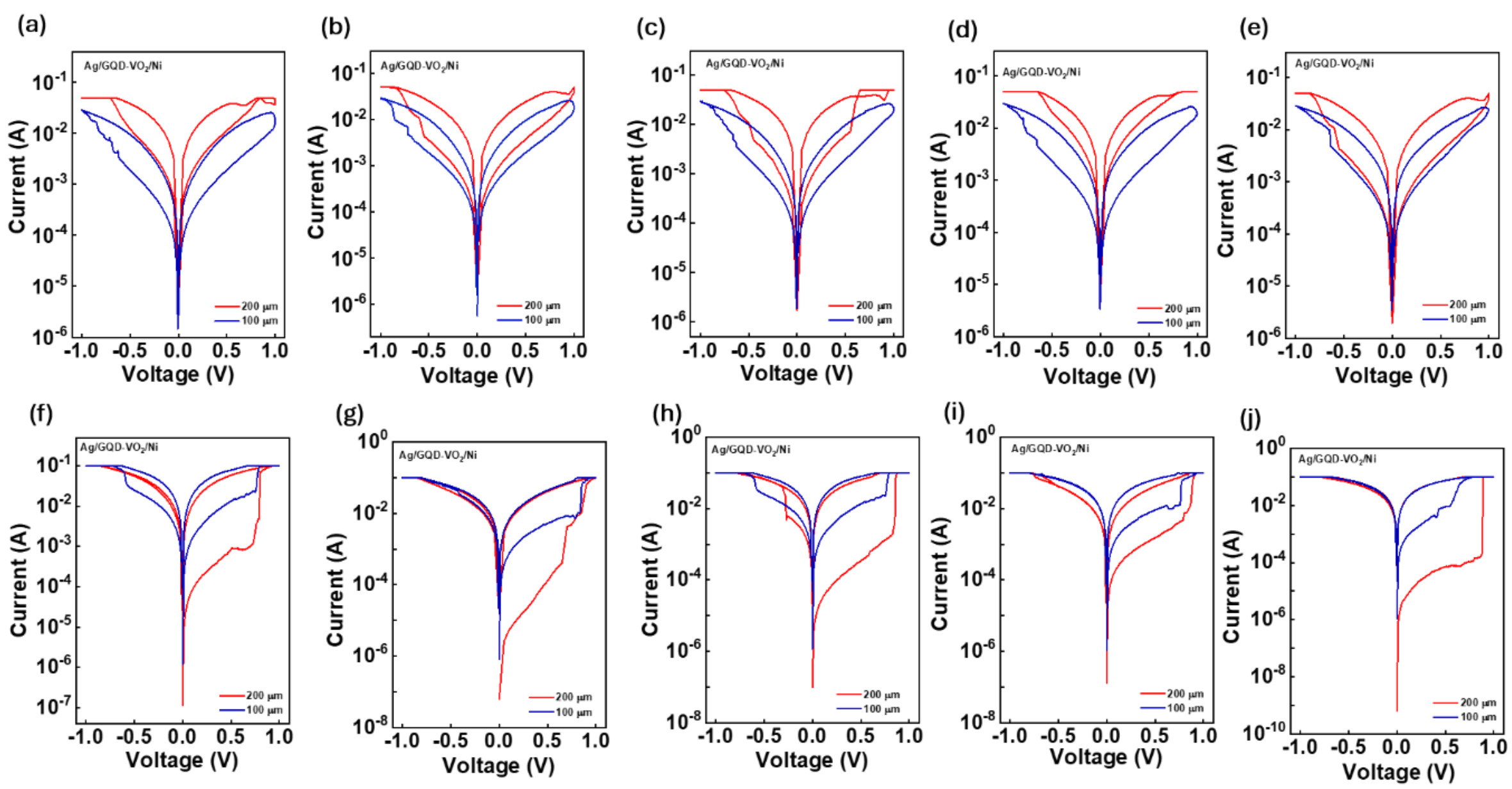

Figure S6. Device-to-Device variation in I-V characteristics for devices with different electrode diameters. (a-e) shows the electrical I-V of Ag/GQD-VO_2/Ni devices 100 μm and 200 μm electrode diameters. (f-j) shows optoelectrical I-V characteristics for both electrode sizes.

Figure S6 illustrates the device-to-device I-V characteristics for electrode diameters of 100 μm and 200 μm. The electrical I-V characteristics **(Figure S6a – e)** reveal that the 100 μm device exhibits current levels on the order of 10^{-2} A, whereas the 200 μm device shows significantly enhanced current. This behavior can be attributed to the electrode area effect. The larger electrode area increases the probability of multiple conductive filament formation, leading to higher current levels due to parallel conductive paths. [4] [5] The optoelectrical I-V characteristics **(Figure S6f-j),** measured under 405 nm laser irradiation, demonstrate digital set and analog reset behavior for both electrode sizes. Notably, the 100 μm device exhibits a narrower switching

window compared to 200 μm device, highlighting the influence of electrode area on switching dynamics.

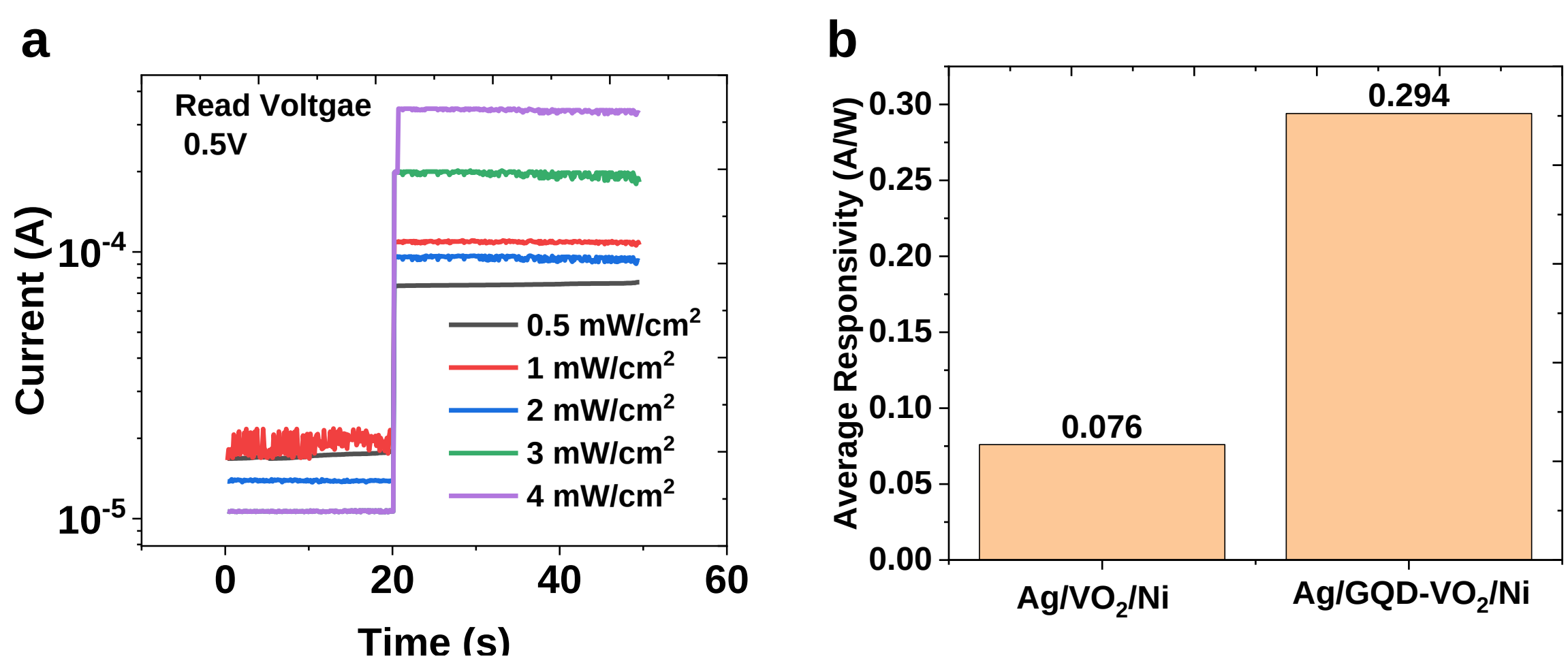

Figure S7. (a) Optical response of the Ag/VO_2/Ni device under varying laser power. (b) calculated responsivity of Ag/VO_2/Ni and Ag/GQD-VO_2/Ni devices.

Figure S7a shows the optical response of the Ag/VO_2/Ni device. The average responsivity of both devices, Ag/VO_2/Ni and Ag/GQD-VO_2/Ni was calculated from their respective optical responses using the following equation at different input powers. It was found that the responsivity of the Ag/GQD-VO_2/Ni device is significantly higher than that of the Ag/VO_2/Ni device, as depicted in **Figure S7b**.

$$R = \frac{I_{Ph} - I_{dark}}{P_{in}}$$

I_{ph} is the photocurrent, where I_{dark} is the on-current and P_{in} is the illumination power.

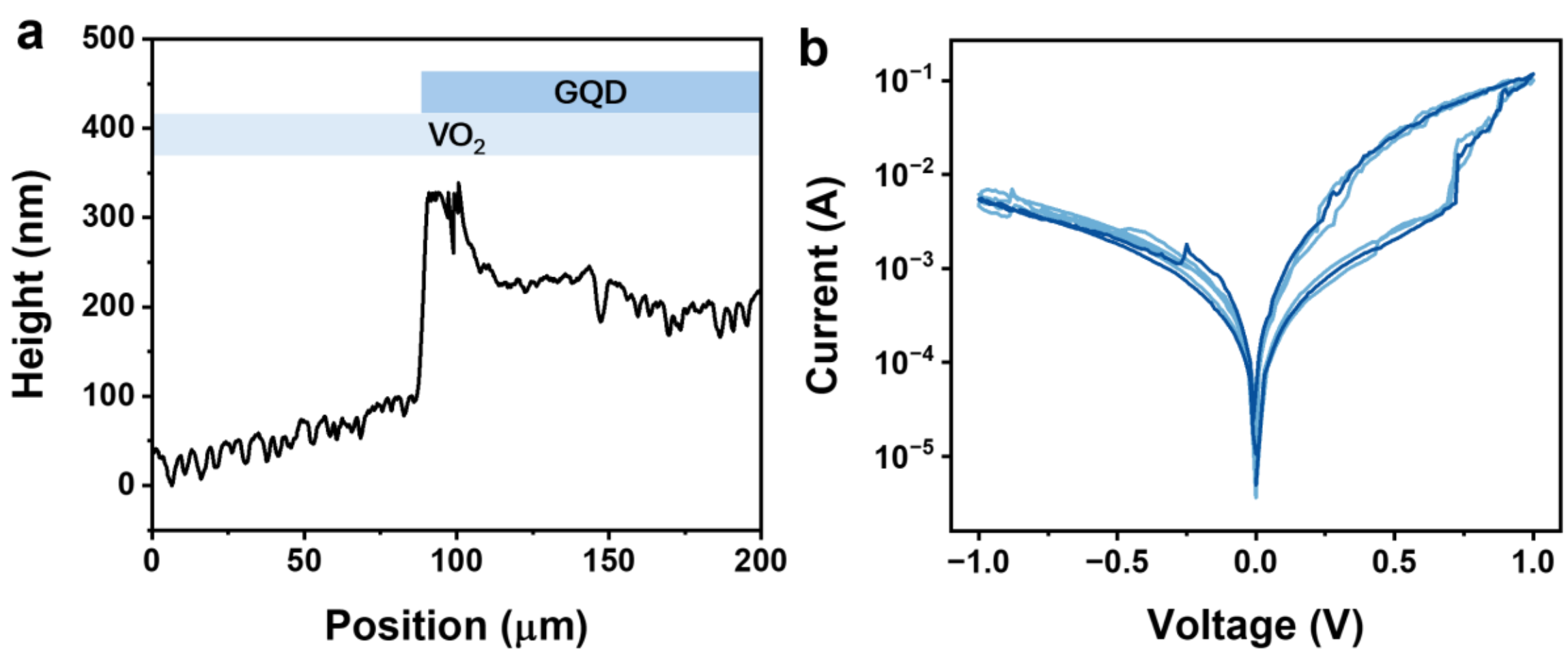

Figure S8. (a) GQD thickness dependence of the Ag/GQD-VO_2/Ni device measured using an Alpha-Step surface profilometer. (b) I–V characteristics of the GQD/VO_2 device prepared using a GQD deposition volume of 2 μL, corresponding to an estimated GQD layer thickness of ~125 nm.

To investigate the influence of GQD layer thickness on the switching characteristics, an additional device was prepared using a reduced GQD deposition volume of 2 μL, resulting in a GQD layer thickness of ~125 nm as measured by Alpha-Step profilometry (**Figure S8a**). As shown in **Figure S8b**, the switching characteristics vary with GQD thickness; however, the difference in GQD thickness, did not result in a proportional enhancement in the switching response indicating that the device response is not simply governed by the bulk amount or thickness of the GQD layer. Together with the bare VO_2 control, these results suggest an important contribution from the GQD/VO_2 interfacial region to the observed switching behavior.

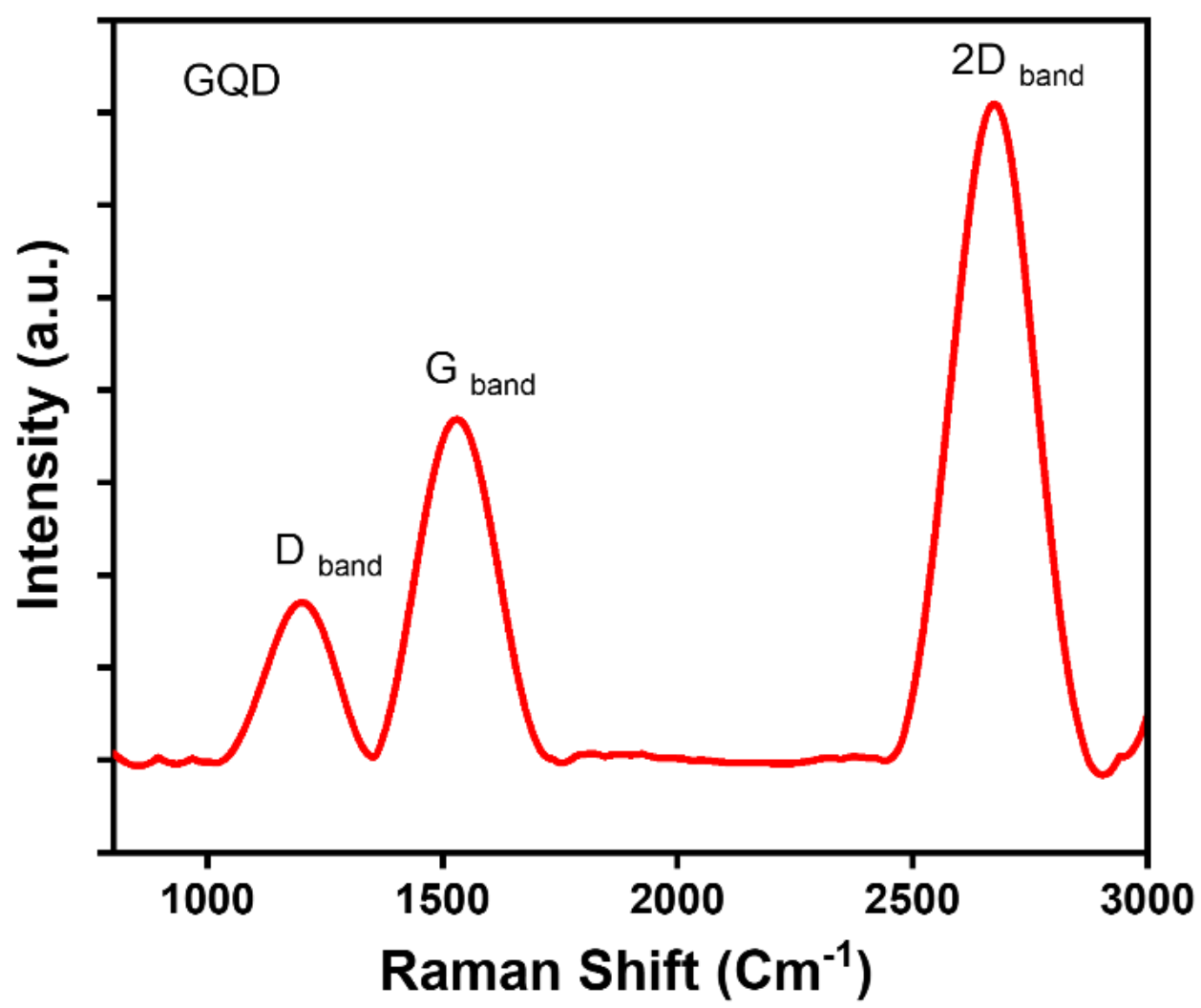

Figure S9. Raman Spectra of GQD.

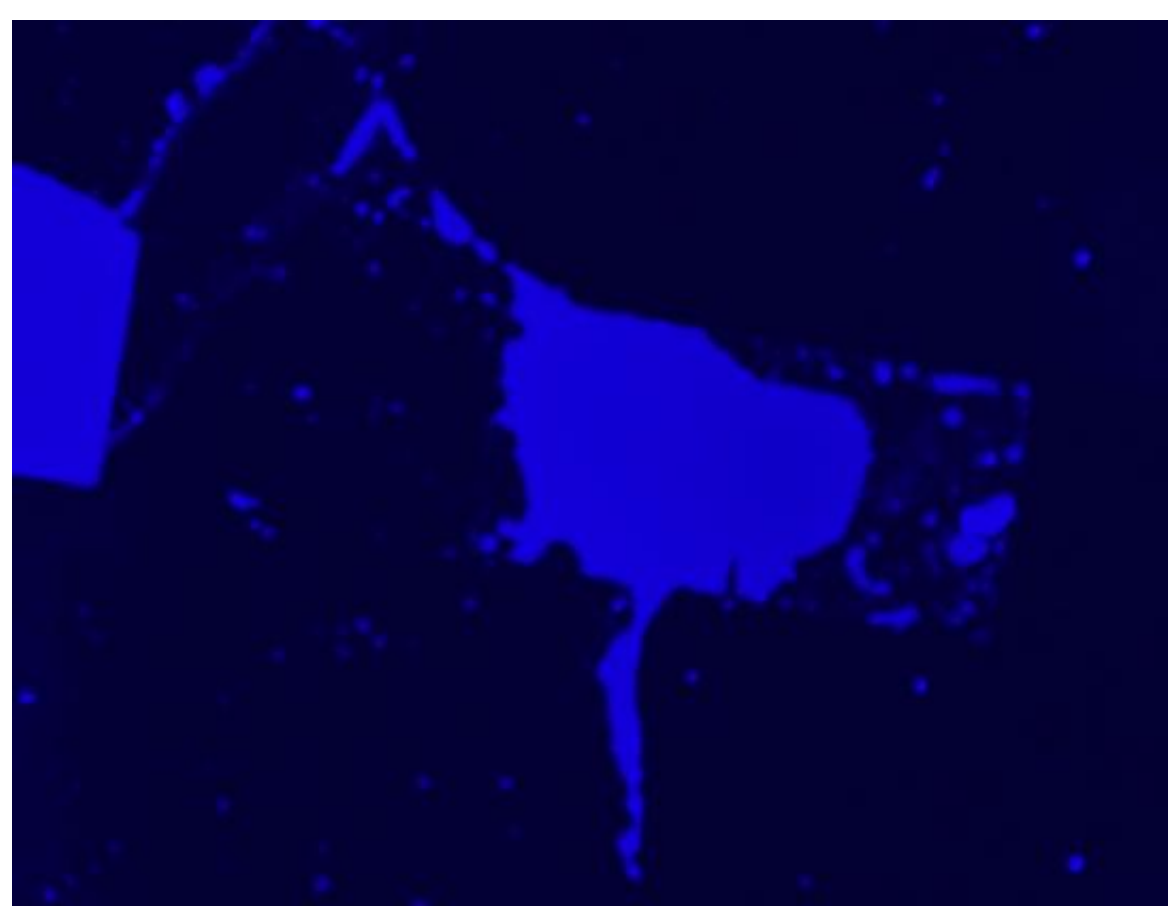

Figure S10. Fluorescence of GQD.

Figure S10 presents the fluorescence image of drop-cast graphene quantum dots (GQDs) on Si/SiO_2 substrates. The observed blue fluorescence emission from the GQDs closely overlaps with the absorption range of VO_2 (350–450 nm), indicating a spectral match between GQD emission and VO_2 absorption. This spectral overlap suggests that the intrinsic fluorescence of GQDs can contribute to the enhanced optical sensitivity and photonic interactions in the

Ag/GQD-VO_2/Ni heterostructure. Such synergistic behavior emphasizes the significance of GQD incorporation, reinforcing the functional role of the GQD–VO_2 heterojunction in tailoring the optoelectronic properties of the device for neuromorphic applications.

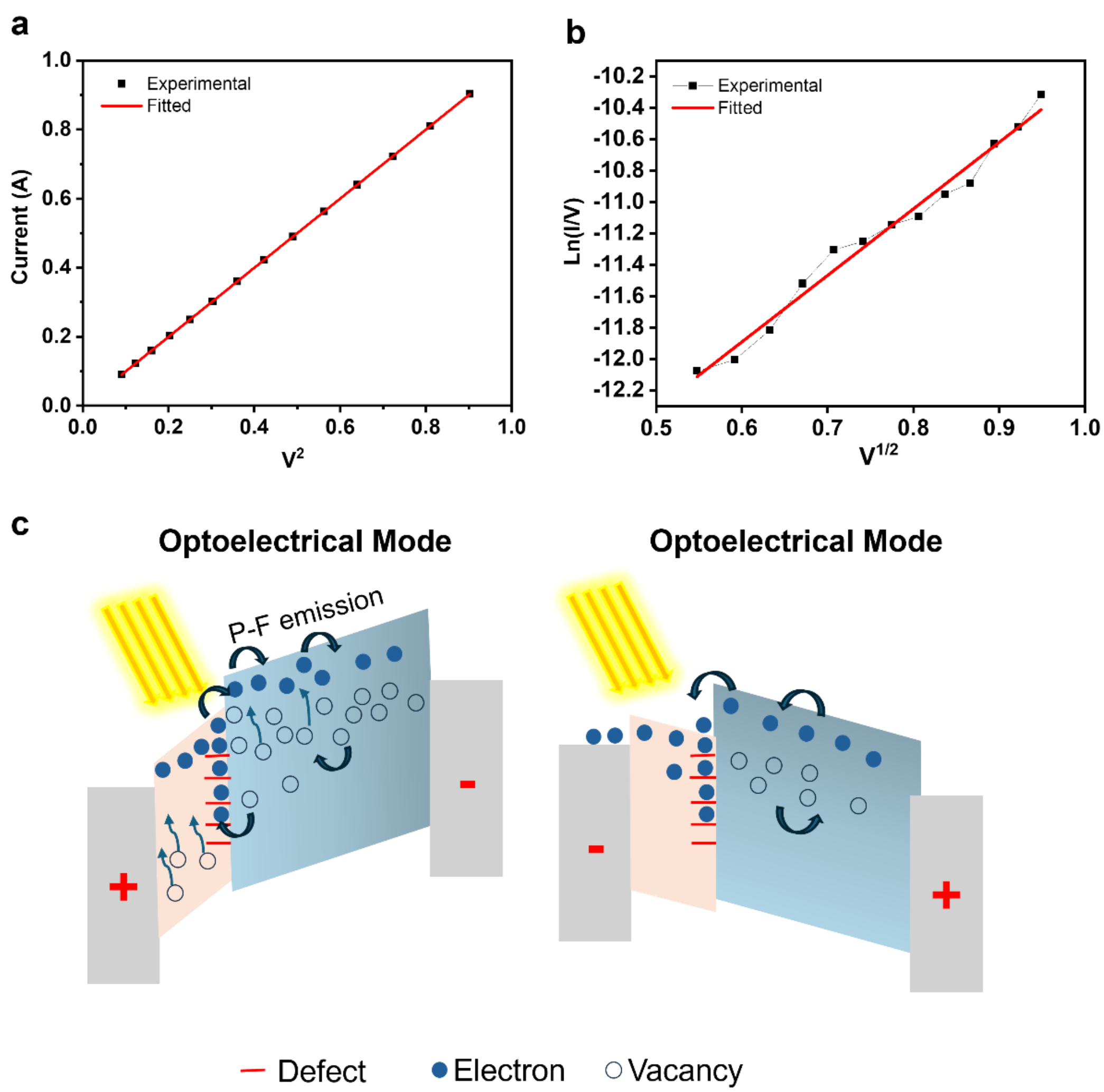

Figure S11. High-voltage conduction mechanism fitting of Ag/GQD–VO_2/Ni device under (a) dark, (b) illumination, and (c) suggested P-F mechanism with trapping sites.

To understand the charge-transport mechanism of the Ag/GQD–VO_2/Ni device, the high-voltage region of the I–V characteristics was analyzed under dark and illuminated conditions (**Figure S11a,b**). In the dark, increased carrier injection at higher electric fields leads to space-charge accumulation, resulting in Child's square-law space-charge-limited conduction. Under

illumination, photogenerated carriers interact with defect-associated trap states within the GQD–VO_2 system. At higher electric fields, the trapping barrier is effectively lowered, facilitating the field-assisted release of trapped carriers and resulting in Poole–Frenkel (P–F)-type conduction. This trap-assisted carrier emission under optoelectrical stimulation is schematically illustrated in **Figure S11c**.

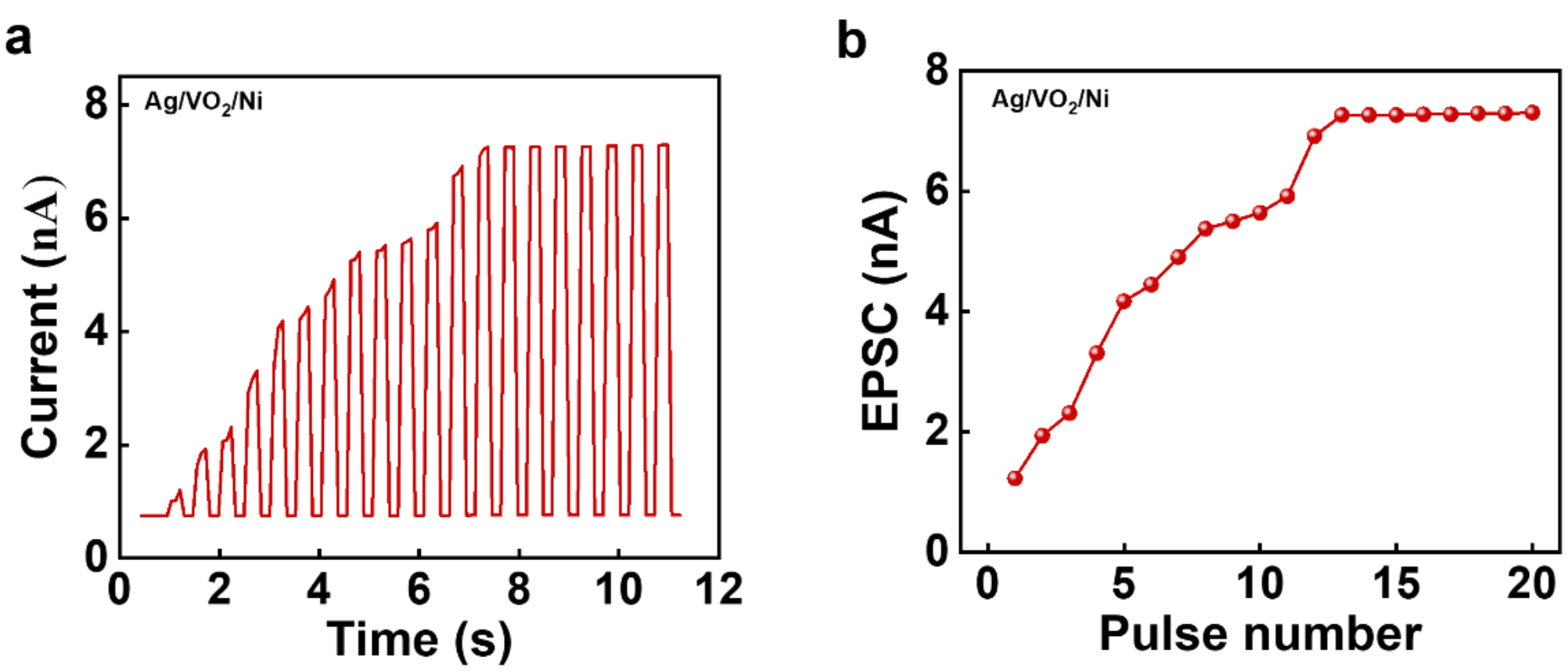

Figure S12. (a) and (b) show the PSC response of the Ag/VO_2/Ni device under 405 nm wavelength laser irradiation at 4 mW/cm^2.

Figure S12 shows the PSC response of the Ag/VO_2/Ni device under 405 nm laser irradiation at an intensity of 4 mW/cm^2 (pulse width 0.5 s). The results demonstrate a gradual increase in PSC with successive optical pulses; however, the overall photoresponse remains relatively low.

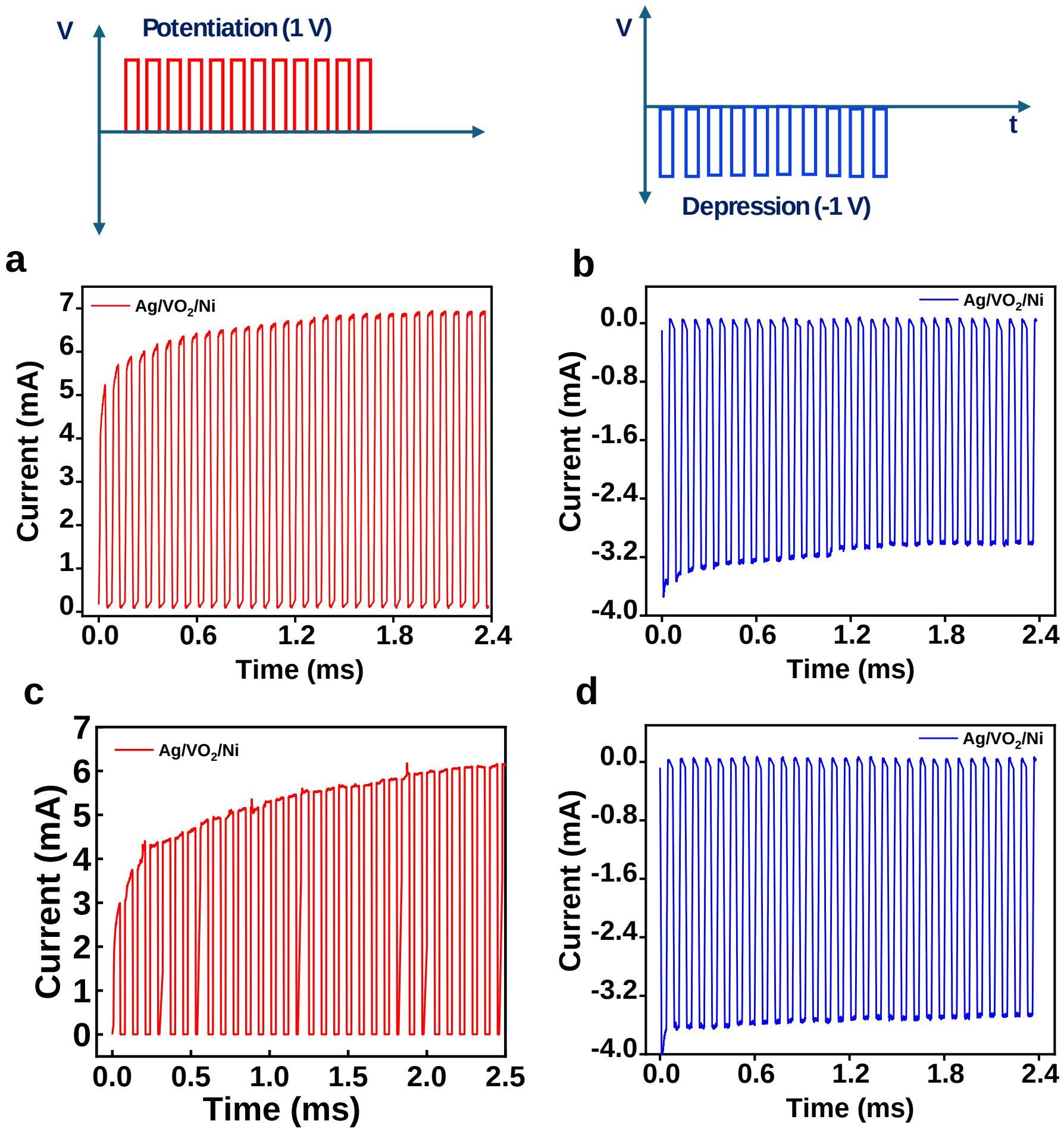

Figure S13. (a) PSC behavior of the Ag/VO_2/Ni device under a +1 V electrical pulse amplitude. (b) PSC behavior under a -1 V electrical pulse amplitude. (c) PSC response under +1 V optoelectronic stimulation, and (d) PSC response under -1 V optoelectronic stimulation.

The post-synaptic current (PSC) response of the VO_2 device was investigated by applying electrical voltage pulses of +1 V (50 µs) and -1 V (50 µs). A positive pulse induces potentiation behavior, while a negative pulse leads to depression, as depicted in **Figure S13a and b**. Subsequently, the PSC response under optoelectronic stimulation was examined for the Ag/GQD-VO_2/Ni device. Under +1 V bias, enhanced potentiation is observed due to light-

induced current modulation, whereas -1 V bias results in depression behavior, as depicted in **Figure S13c and d.**

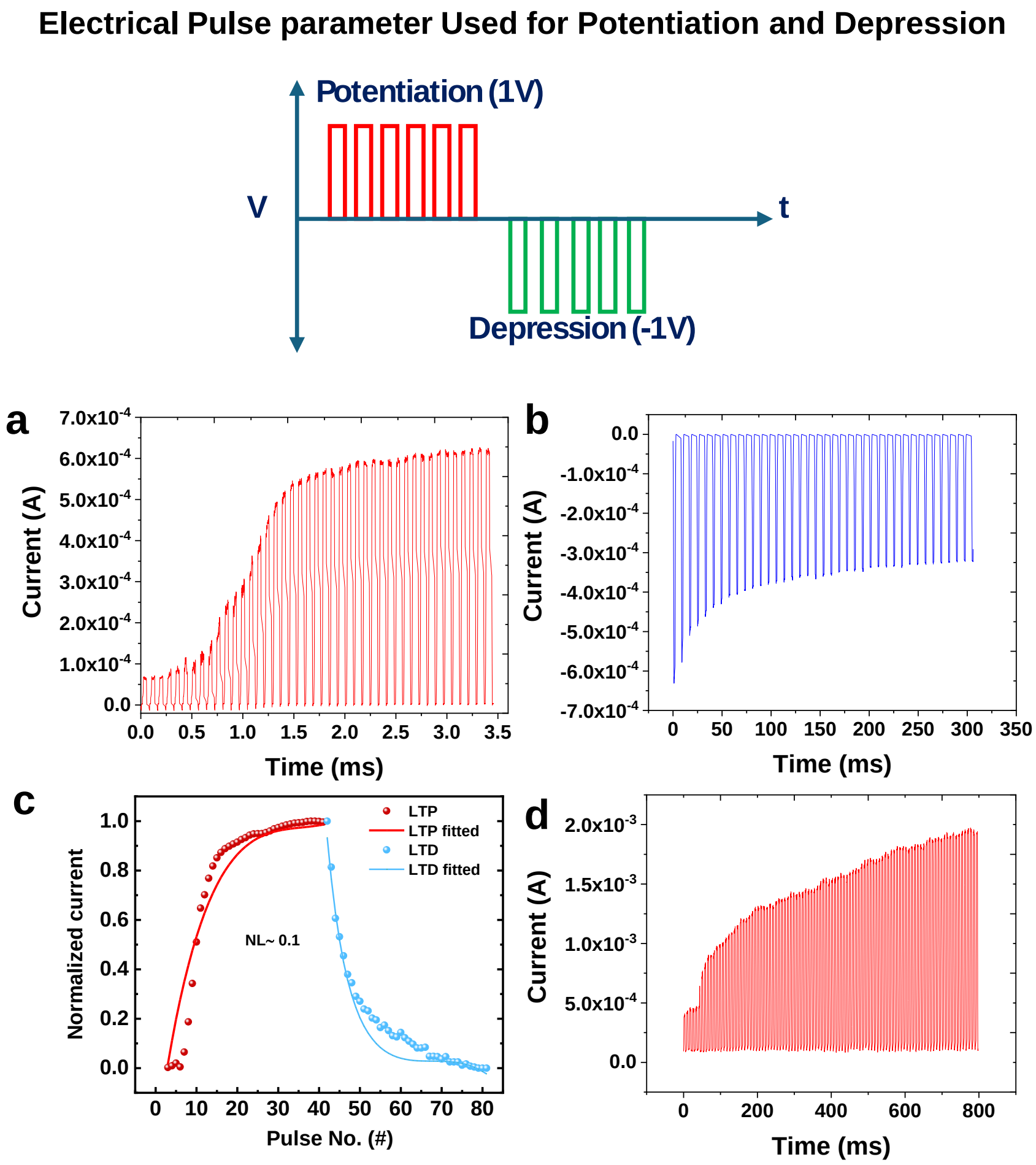

Figure S14. (a) PSC behavior of the Ag/GQD-VO_2/Ni device at 1 V amplitude. (b) PSC behavior of the Ag/GQD-VO_2/Ni device at -1 V. (c) Linearity of the Ag/GQD-VO_2/Ni device during electrical stimulation. (d) The combined effect of electrical and optical pulses on the PSC.

Potentiation and depression are fundamental concepts in neuromorphic computing, representing the processes of learning and forgetting, analogous to the functioning of the human brain. Mimicking these properties has become increasingly common in various memristive devices. In

this study, we stimulated the Ag/GQD-VO_2/Ni device using electrical, optical, and combined electrical-optical inputs, enabling a bimodal approach to tune the device's conductance states, referred to as synaptic weights in neuromorphic computing.[6] **Figure S14a** illustrates the post-synaptic current (PSC) behavior of the Ag/GQD-VO_2/Ni device, achieved by applying 80 consecutive electrical pulses of the same amplitude +1 V (40 μs). Similarly, IPSC was induced using pulse amplitude -1 V (150 μs), as shown in **Figure S14b**. **Figure S14c** shows the nonlinearity of the of potentiation and depression of ~ 0.1. The enhanced excitatory postsynaptic current (EPSC) behavior observed under combined electrical and optical stimulation, as shown in **Figure S14d**, highlights the prominent optoelectronic characteristics of the fabricated Ag/GQD-VO_2/Ni device.

Energy consumption per synaptic event or spike is calculated from **Figure S14d** by the formula

$$E = V \times I \times t$$

Where V is pulse amplitude (0.7 V), I is current (3.0×10^{-4} A), and t is the time pulse width (150 μs). The electrical energy consumption for the first potentiation pulse in **Figure S14d** is therefore estimated as 31.5 nJ under optoelectrical operation. This estimate excludes the incident optical energy and the power consumption of the illumination source.

Furthermore, the energy consumption per synaptic event or spike and dynamic range of both Ag/VO_2/Ni and Ag/GQD-VO_2/Ni devices were systematically evaluated.

Table-S1

	Energy Consumption (nJ/spike)		
	Electrical	Optical	Opto-electrical
VO_2	280	1.82	220
GQD-VO_2	21.6	54.2	31.5

	Dynamic range		
	Electrical	Optical	Opto-electrical
VO_2	1.32	6.04	2.06
GQD-VO_2	35.21	14.28	44.29

As summarized in **Table-S1** the incorporation of GQDs changes the energy response of the device. The Ag/VO_2/Ni device exhibits an electrical energy consumption of ~280 nJ/spike. Under optical stimulation, the energy consumption is significantly reduced to ~ 1.82 nJ/spike, primarily due to the relatively low photocurrent response of VO_2 **(Figure S7b).** Under optoelectrical stimulation, the estimated electrical energy consumption is ~ 220 nJ/spike. In contrast, the Ag/GQD-VO_2/Ni device demonstrates a substantial reduction in energy consumption. The electrical energy consumption is measured to be 21.6 nJ/spike, and the optical stimulation energy consumption is ~ 54.2 nJ/spike, and the electrical energy consumption decreases to 31.5 nJ/spike under optoelectrical operation.

The dynamic range (DR) of the Ag/VO_2/Ni and Ag/GQD-VO_2/Ni device was determined using DR = G_{max}/G_{min}, where G_{max} and G_{min} are conductance values obtained from LTP measurements, respectively.[7] The DR of the VO_2 device under electrical measurements is ~ 1.32, for optical measurements ~ 6.04, and under optoelectronic operation ~ 2.06, **extracted from Figure S12 and S13.** In contrast, the GQD-VO_2 device exhibits a significant enhancement in the dynamic

range, with values of ~ 35.21 under electrical operation, ~14.28 under optical operation, and ~ 44.29 under optoelectronic operation **(Figure 3e, manuscript)**, as summarized in **Table-S1**. indicating that GQD and light exposure enhance the carrier-trapping process, which improves device synaptic performance.

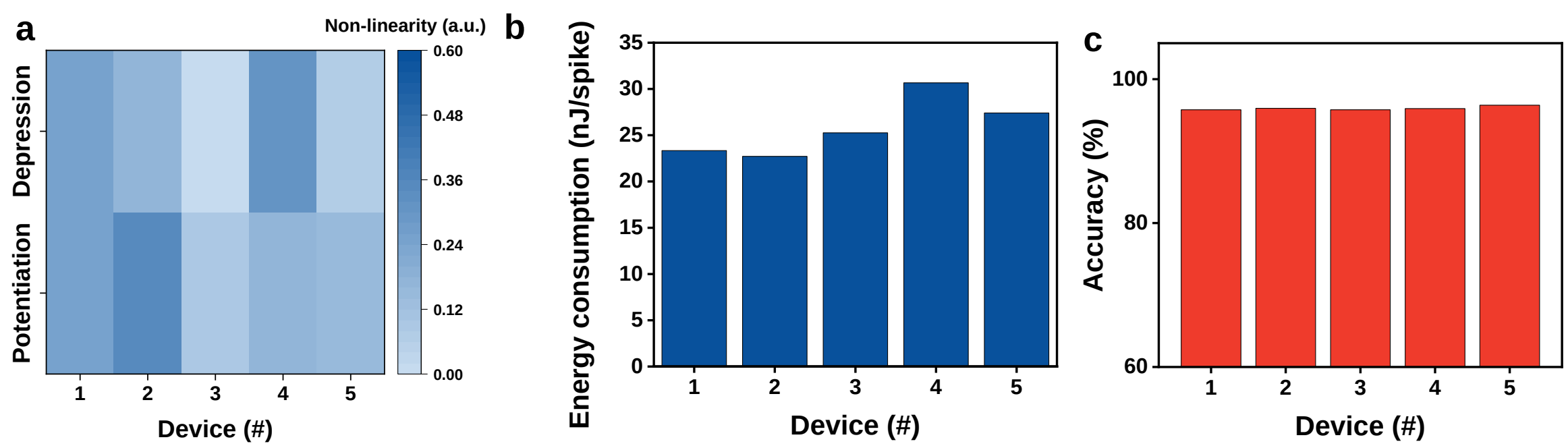

Figure S15. Device-to-device variation in synaptic characteristics and neural-network performance. (a) Synaptic non-linearity, (b) energy consumption, and (c) corresponding neural-network accuracy obtained from five devices fabricated within the same batch.

Device-to-device variation was evaluated using five devices fabricated within the same batch, as shown in **Figure S15**. Variations were observed in synaptic non-linearity and energy consumption, reflecting differences in individual device characteristics. Nevertheless, neural-network accuracy remained consistently around 96%, with less than 1 percentage-point variation among the devices. These results indicate that the measured device variability has a limited influence on network performance within the experimentally evaluated range.

Table -S2

Device structure	Stimulation	Set Reset Voltage	Wavelength used	Energy consumption per synaptic event	Emulated synaptic function	Cognitive functions	Image recognition accuracy	Ref
Pt/WO_x/Pt	Electrical	2/-2 V	N/A	N/A	Synaptic plasticity and Metaplasticity	STDP (spike time-dependent plasticity)	N/A	[8]

$Pt/HfO_x/W$	Electrical	-5/5 V	N/A	0.5 nJ	Synaptic plasticity and Metaplasticity	Adaptation (Single learning)	N/A	[9]
$Pd/HfO_x/TiN$	Electrical	2/-2	N/A	N/A	Metaplasticity	N/A	N/A	[10]
$Al/AlO_x/Graphene/SiO_2$	Electrical	2.3/-2.3	N/A	10 fJ	Metaplasticity	N/A	N/A	[11]
$Pt/GQDs/FeO_x/Pt$	Electrical	-1/1	N/A	N/A	Metaplasticity	Catastrophic Forgetting in Neuromorphic Networks	N/A	[12]
QDs/ITZO-based transistor	Optical	-	450 nm 550 nm	N/A	Optical synaptic plasticity	Photopic and scotopic adaptation, MNIST recognition	93% (Digit)	[13]
CdSe Photodetector and IGZO-based transistor	Optical	-	465 nm 525 nm 620 nm	N/A	Optical synaptic plasticity	Photopic and Scotopic adaptation	N/A	[14]
$ITO/ZnO/WO_x/W$	Optical	2/-2	350 nm	N/A	Optical synaptic plasticity	Adaptation, Pattern recognition	94 %	[15]
ITO/P3HT:PC71BM/Au as the light sensor and ITO/CBI@P(VDF-TrFE)/Cu memristor unit	Optoelectrical	1/-1	400 nm 500 nm 600 nm	Power consumption ~1 pW	N/A	Current-based Adaptation	N/A	[16]
$Au/MAPbI_3/P3HT/ITO$ transistor	Optoelectrical	-	626 nm	N/A	Optical synaptic plasticity	Current-based Adaptation	N/A	[17]
$FTO/TiO_2/ZFO/Ag$	Electrical and Optical	0.4/-2.5	445 nm	180 nJ/spike	Electrical synaptic Plasticity and optical synaptic plasticity	Pavlovian associative learning and Morse code recognition	N/A	[18]
$Au/CuI/TiO_x/FTO$	Optoelectrical	-	405 nm	N/A	Optical Synaptic Plasticity (STP to LTP)	MNIST recognition (Reservoir Computing)	97.26 %	[19]

p-Si/LuOx/$Cu_3(HHTP)_2/MoO_3$/ITO	Optoelectrical	-	255 nm	10^{-10} J/spike	Optical synaptic plasticity	Photo adaptive behaviour of Human visual system, MNIST recognition (reservoir computing)	94.10 %	[20]
ITO/P_3HT/Al	Optical	-	445 nm 520 nm 660 nm	N/A	Optical Synaptic Plasticity	Photopic Adaptation with letter alphabets and Image recognition	90 %	[21]
ITO/$HfTiO_x$/Au-Np/$(TiO_x)Al_2O_3$/TaN	Optical	-	405 nm 450 nm 532 nm	N/A	Optical Synaptic Plasticity	Visual Imaging, logic function, Associative learning, 16-digit pattern evaluation	N/A	[22]
ITO/Pbs@PMMA/ZnO/Pbs@PMMA/Ag	Optical	-	365 nm		Optical synaptic plasticity	Photopic and Scotopic adaptation	N/A	[23]
ITO/PEDOT:PSS/APbI3/PMMA/Ag	Electrical	0.32 V	-	-	Electrical synaptic plasticity	Hebbian and Anti-Hebbian learning rules, MNIST recognition	98.88 %	[24]
ZnO/Nb_2O_5 Heterostructure	Optical	-	620 nm 460 nm	1.29 nJ	Optical synaptic palsticity	MNIST	ANN: 88.43% CNN: 84.63%	[25]
InSe nanofilm	Optical	-	254 nm	0.51 fJ	Optical synaptic plasticity	Emotion learning, memory process simulated by letter recognition	-	[26]

Ta/IGZO/$Hf_{0.5}Zr_{0.5}O_2$/TiN	Optical	-	405 nm 450 nm 520 nm	-	Optical synaptic plasticity	Pavlovian associative learning, optical reservoir computing and FMNIST pattern recognition	85.53 %	[27]
**Ag/GQD-VO_2/Ni**	**Optical, electrical, optoelectrical**	**1.5/-1.5**	**405 nm, 1550 nm, White light**	**31.5 nJ/spike**	**Electrical synaptic plasticity, Optical Synaptic plasticity, and Opto-electrical synaptic plasticity**	**Optoelectrical conditioning (quantitative analysis), positive reinforcement, Negative reinforcement, Associative and Non-associative learning, MNIST and FMNIST image recognition**	**95.58% (Digit) 84.13% (Fashion)**	**This Work**

From the comparative analysis of reported studies presented in the above **Table-S2**, it is observed that although more complex heterostructures with multiple layers have been employed, most of the devices exhibit only optical synaptic characteristics. In contrast, our proposed device demonstrates multifunctional behavior under electrical, optical, and optoelectrical stimulations, with optoelectrical conditioning characterized by history-dependent conductance modulation under repeated stimulation.

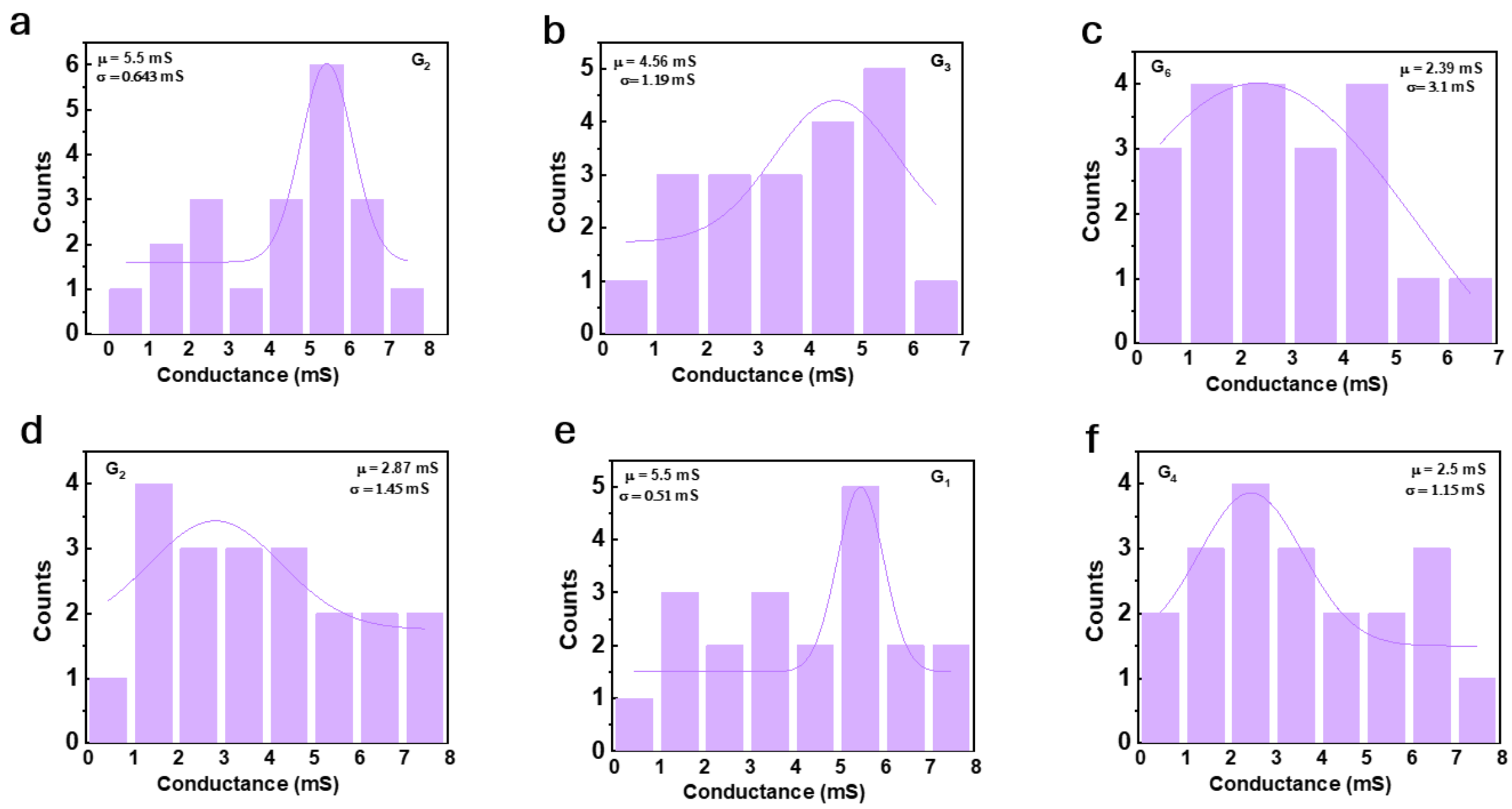

Figure S16. Histograms of the experimental conductance distribution measured for six cycles of LTP and LTD of Fig. 4a (manuscript).

Table -S3

conductance	Mean (μ)	Standard deviation (σ)
G2 (P)	5.5 mS	0.64 mS
G3 (P)	4.56 mS	1.19 mS
G6 (P)	2.39 mS	3.1 mS
G1 (D)	5.5 mS	0.51 mS
G2 (D)	2.87 mS	1.45 mS
G4 (D)	2.5 mS	1.15 mS

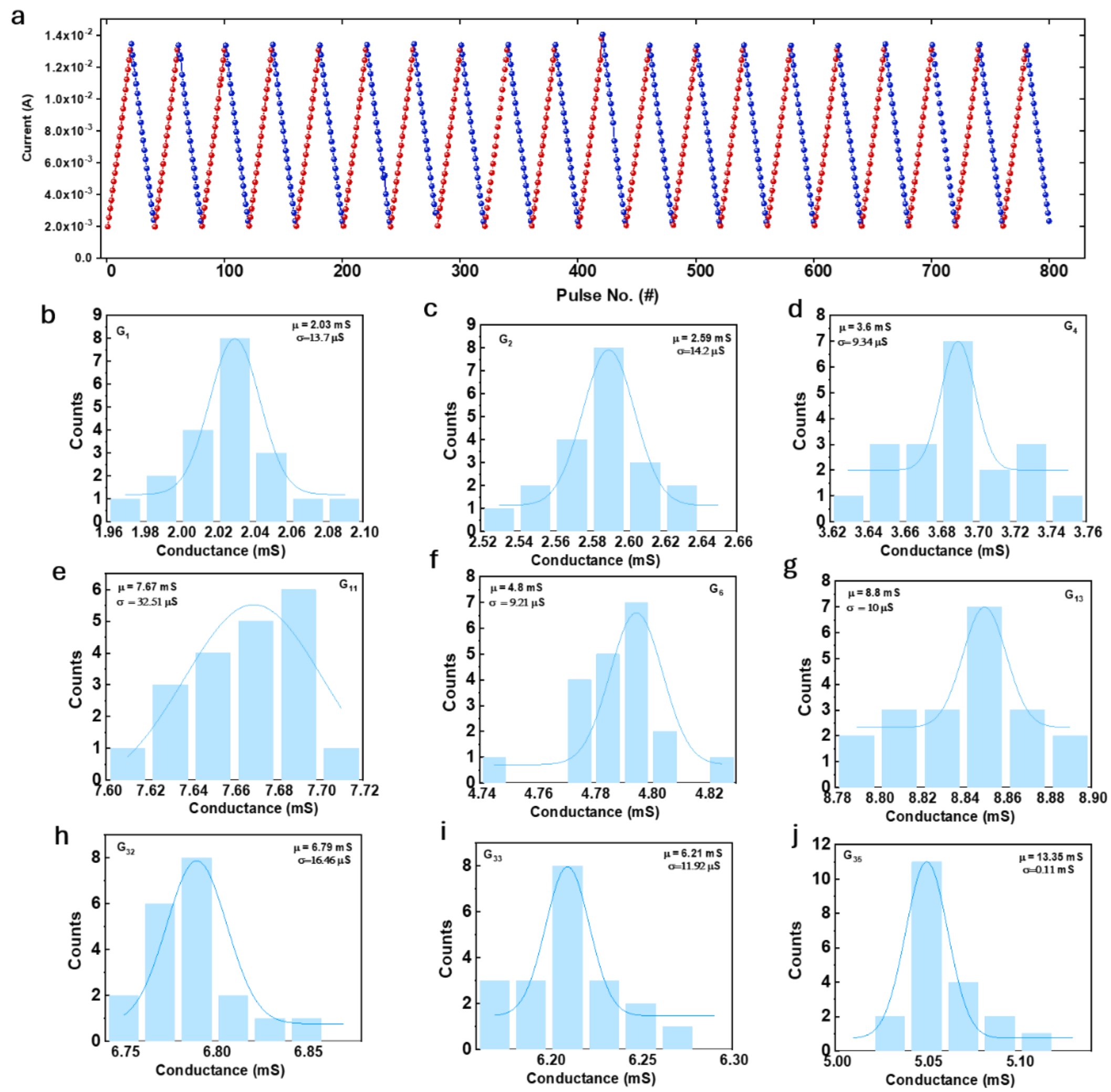

Figure S17. Consecutive 20 cycles of electrical potentiation and depression in Ag/GQD-VO_2/Ni device under dark conditions. Histograms of the experimental conductance distribution measured for 20 cycles of LTP and LTD.

Figures S16 and S17 show the conductance distribution of the Ag/GQD-VO_2/Ni devices measured under dark conditions. The cycle-to-cycle conductance variability was statistically analyzed fitting LTP (P) and LTD (D) conductance states using a Gaussian distribution function. The mean (μ) and standard deviation (σ) extracted from the fitting reveal limited cycle-to-cycle fluctuation within a device, whereas device-to-device variation can be attributed to minor fitting

uncertainity or internal device-dependent mechanisms such as defect distribution.[28] The corresponding values are tabulated below.

Table -S4

conductance	Mean (μ)	Standard deviation (σ)
G1 (p)	2.03 mS	13.7 μS
G2 (P)	2.59 mS	14.2 μS
G4 (P)	3.6 mS	9.34 μS
G11 (P)	7.67 mS	32.51 μS
G6 (P)	4.8 mS	9.21 μS
G13 (P)	8.8 mS	10 μS
G32 (D)	6.79 mS	6.21 mS
G33 (D)	6.21 mS	11.92 μS
G35 (D)	13.35 mS	0.11 mS

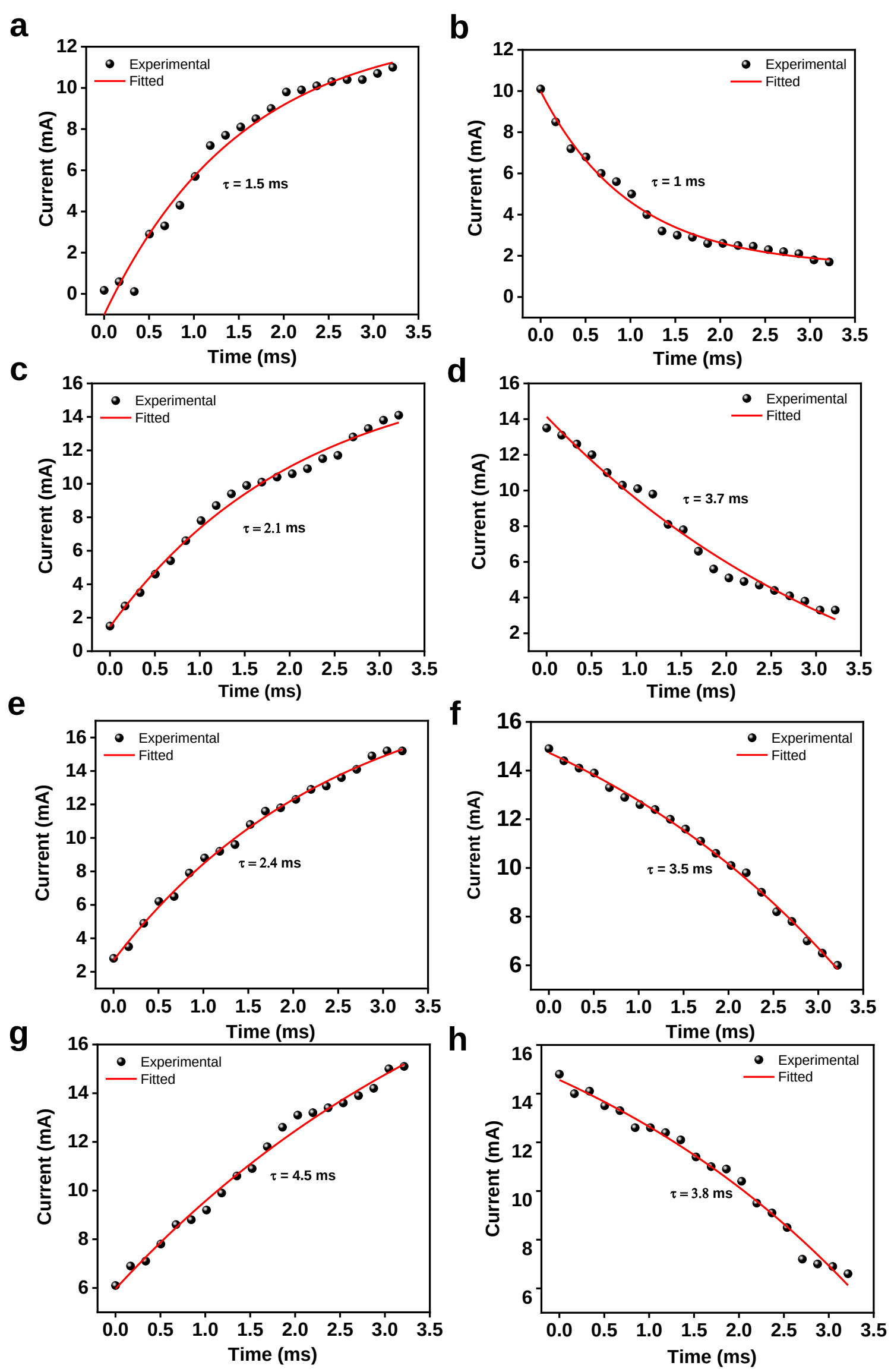

Figure S18. (a-h) Fitting results of LTP and LTD for time constant (τ) for 1-4 cycles before and after optoelectrical conditioning.

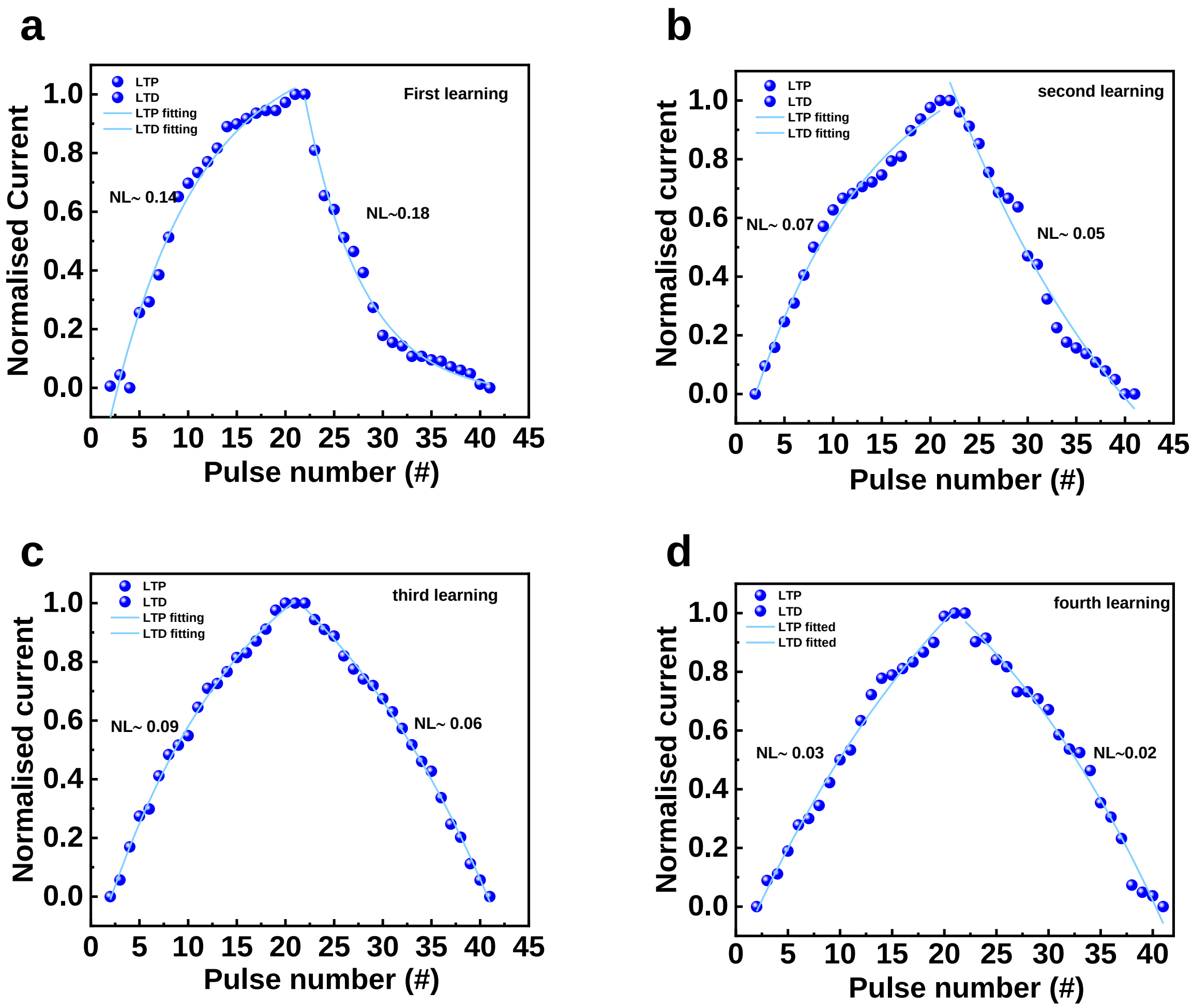

Figure S19. Non-linearity fitting of before and after optoelectrical conditioning

Table S5: time constant (τ) values as per cycle no.

	Cycle No.	LTP (τ) (ms)	LTD (τ) (ms)	Non linearity (LTP)	Non linearity (LTD)
Before optoelectrical conditioning	1	1.5	1	~ 0.1	~ 0.18
	2	2.1	3.7	~ 0.07	~ 0.05
	3	2.4	3.5	~ 0.09	~ 0.06
After optoelectrical conditioning	4	4.5	3.8	~ 0.03	~ 0.02

Table S6: Variation in current change (ΔI) across successive learning cycles during optoelectrical conditioning of the Ag/GQD–VO_2/Ni device (Figure 4b).

Parameter	ΔI (mA)
First learning	1.53
Second learning	3.13
Third learning	6.43
Fourth learning	6.43
Fifth learning	6.43
Sixth learning	6.43

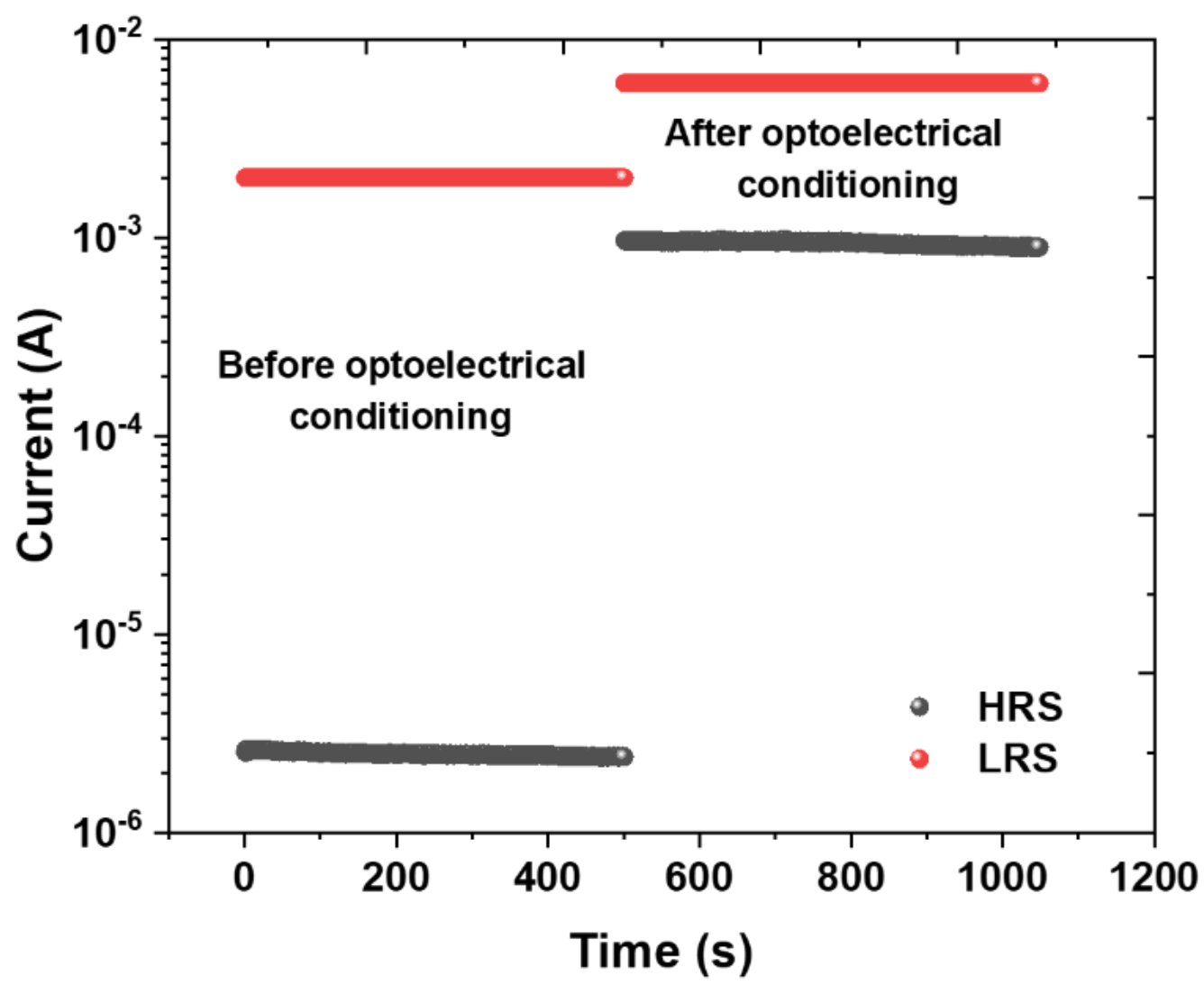

Figure S20. Retention characteristics of the Ag/GQD–VO_2/Ni device both prior to and following optoelectrical conditioning for 500 s under a read voltage of 0.02 V and a set voltage of 0.8 V.

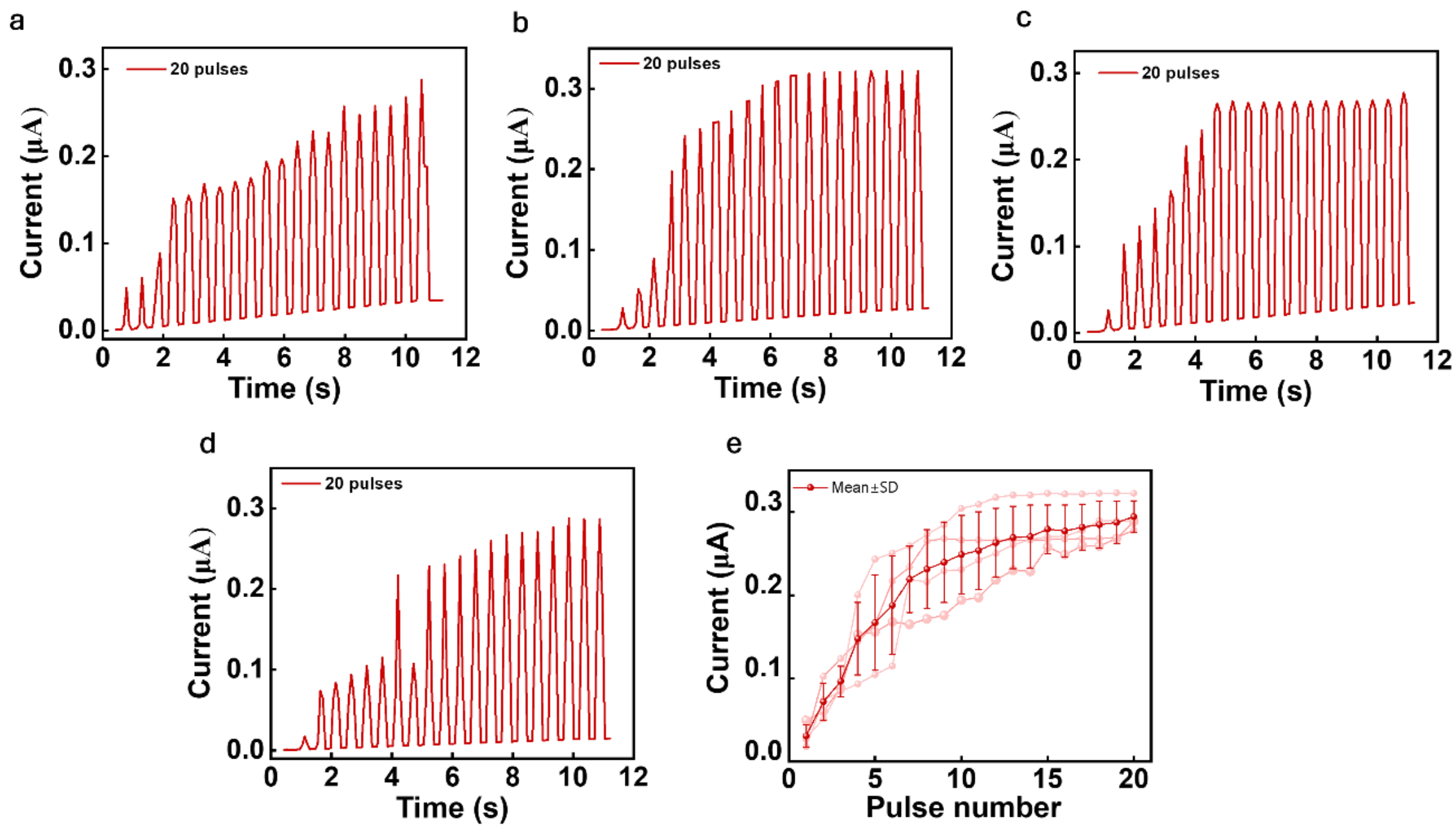

Figure S21. (a-d) illustrates the cycle-to-cycle variability of the optical synaptic response in the Ag/GQD-VO_2/Ni device. (e) shows the mean and standard deviation (SD) of the observed variability.

To further examine this behavior, cycle-to-cycle variability measurements of the optical response were conducted on the Ag/GQD-VO_2/Ni device, as illustrated in the **Figure S21a-d**. The measurements were carried out using 20 optical pulses, indicating mid-term memory (MTM) with a 405 nm laser (pulse width 0.5 s). A similar non-monotonic response was observed across repeated cycles. The corresponding standard deviation (SD) is presented in **Figure S21e.** This variability is attributed to the stochastic trapping and detrapping of photogenerated carriers associated with defect states and interfacial traps within the GQD-VO_2 heterostructure. In contrast, the Ag/GQD-VO_2/Ni device exhibits a stable response under optoelectrical stimulation **(Figure 3e and f, manuscript),** which is attributed to the presence of an external electric field, which enables controlled carrier injection and directional transport. To improve the monotonicity

of optical response, interface engineering strategies can be implemented to regulate charge trapping dynamics and minimize variability, which will be the focus of future work.[29]

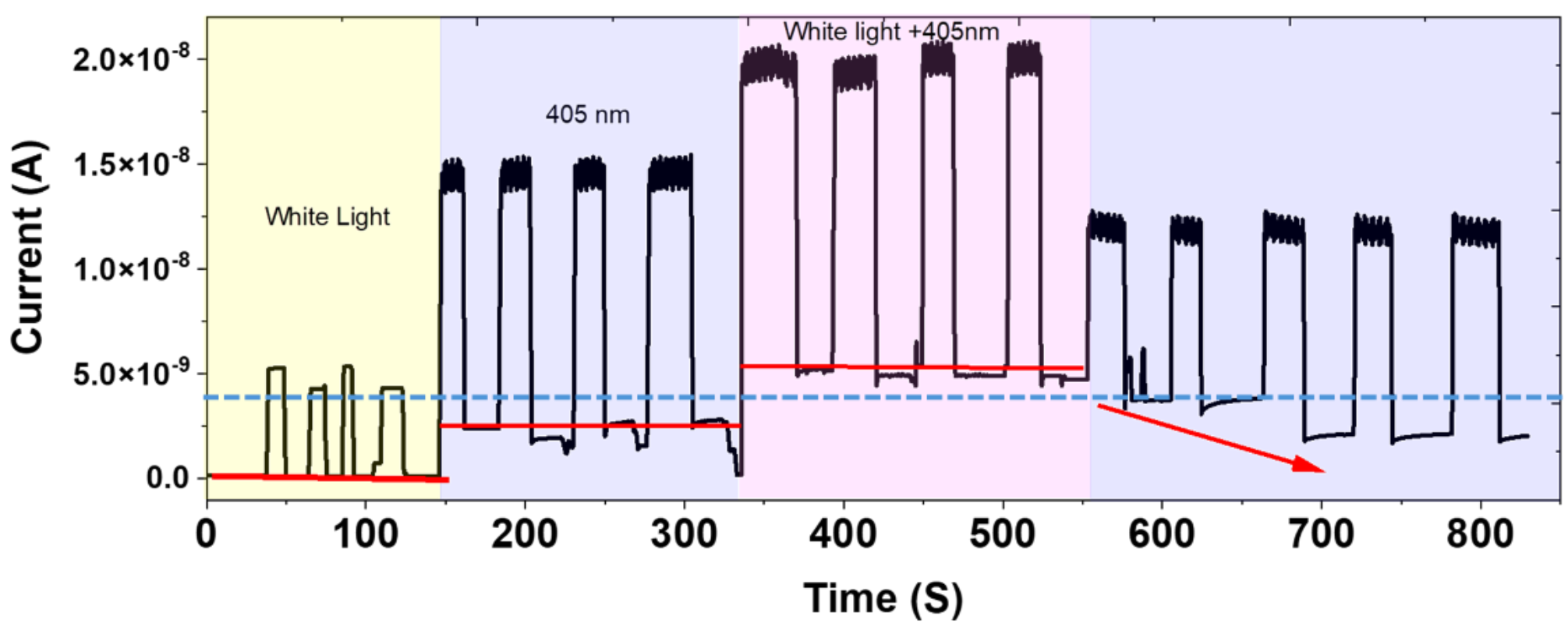

Figure S22. Associative learning behavior of the Ag/GQD-VO_2/Ni device under 405 nm and white light illumination, demonstrating positive and negative reinforcement.

Figure S22 shows the results following the combined application of white light and 405 nm laser, demonstrating a reinforcement effect. The blue dotted line represents the threshold current (4.8 x 10^{-9} A) above which associative behaviour is defined in this work. When the white light is subsequently turned off, the device response remains comparable to the previously observed 405 laser response. This behavior may be attributed to the relatively weak synaptic response induced by white light alone, which is insufficient to produce a significant negative reinforcement effect.

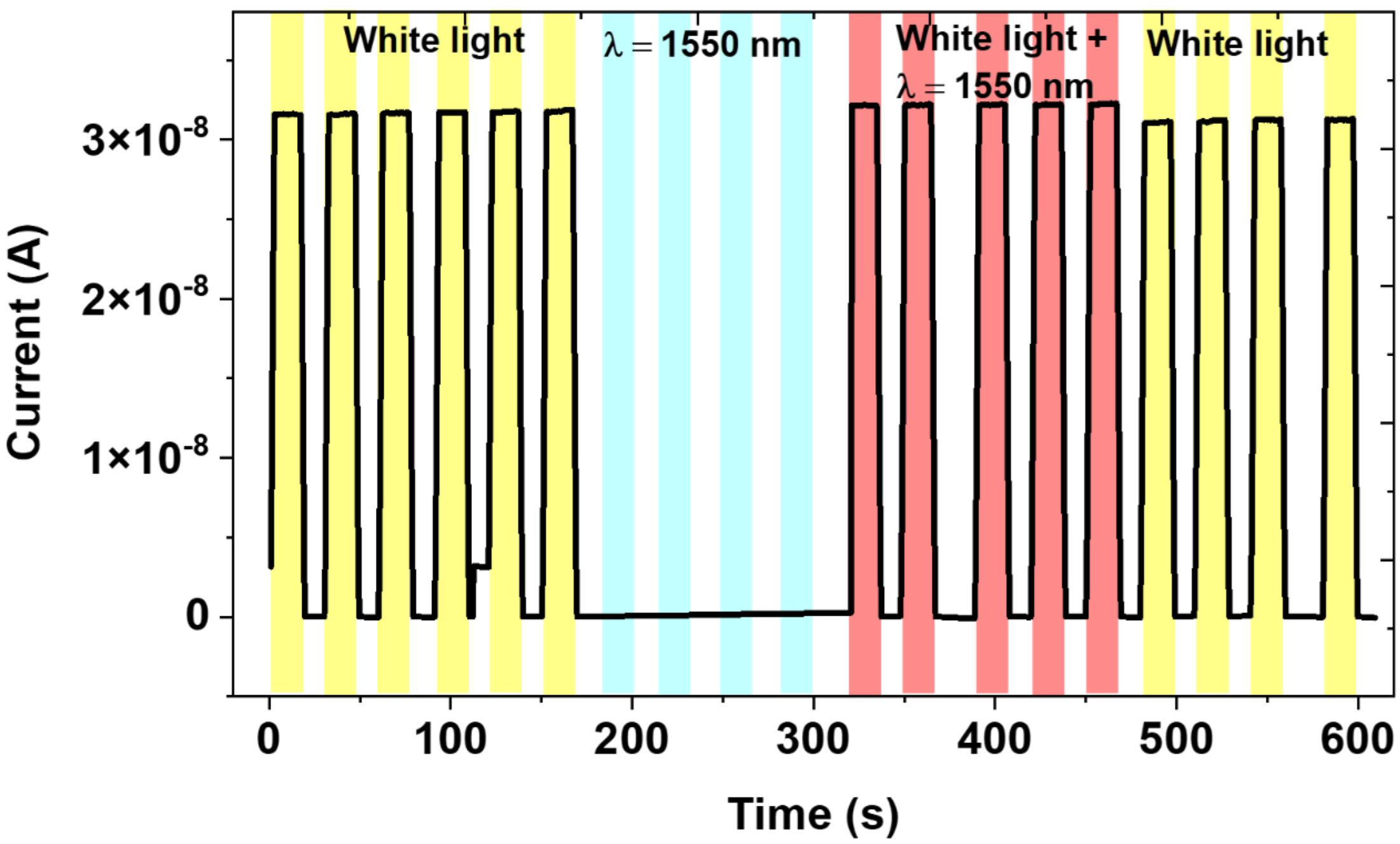

Figure S23. Non-associative learning behavior of the Ag/GQD-VO_2/Ni device under white light and 1550 nm illumination.

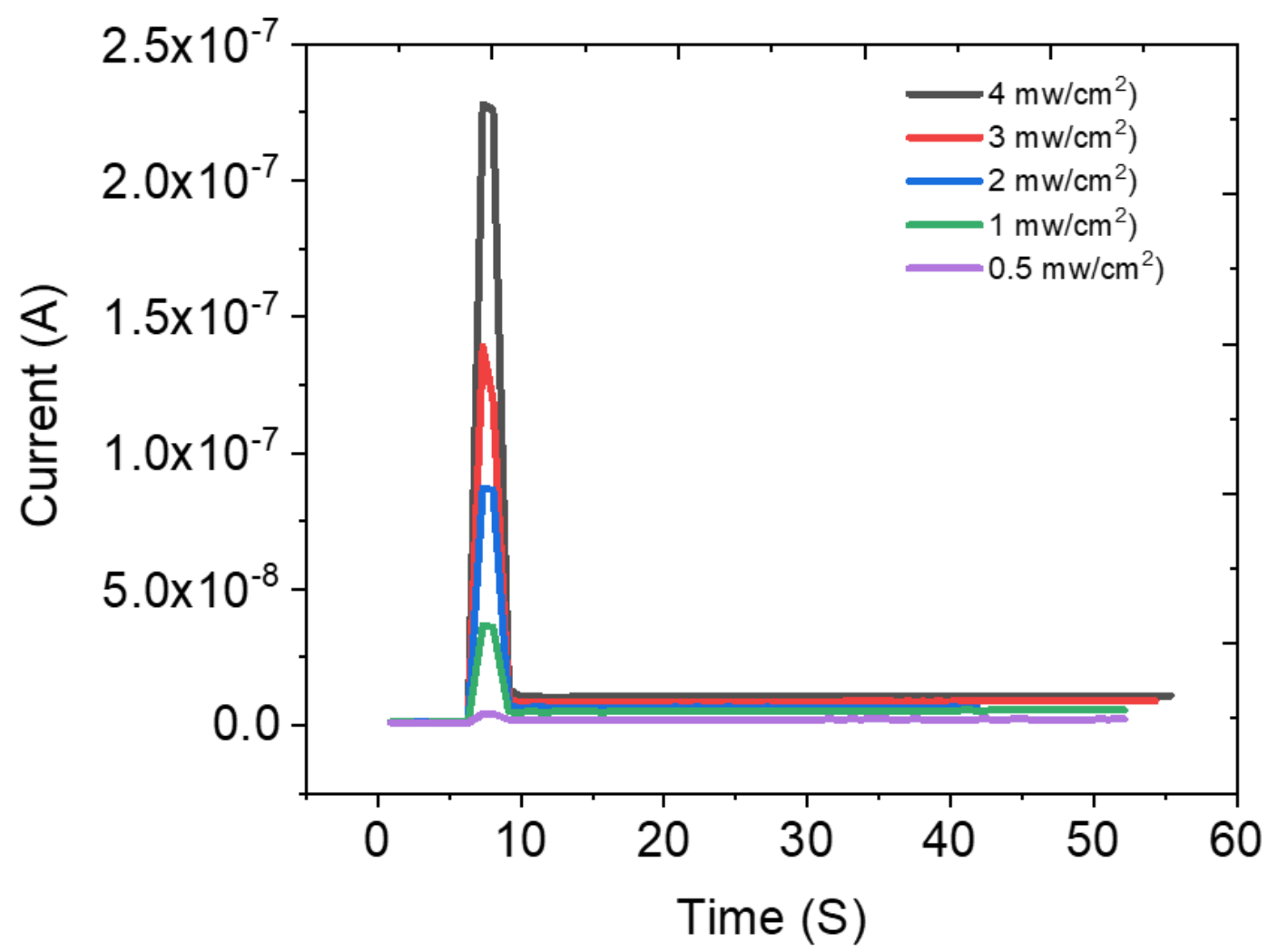

Figure S24. Laser power-dependent response of the single pulse for associative learning of the Ag/GQD-VO_2/Ni device.

Figure S24 presents a comparative analysis of the 405 nm laser pulse-dependent response at various power levels for the Ag/GQD-VO_2/Ni device. The results demonstrate that the current response increases with laser power, highlighting the device's sensitivity to optical intensity. This behavior supports the role of laser power as a modulatory parameter in associative learning and further emphasizes the tunability of the optoelectronic synaptic response.

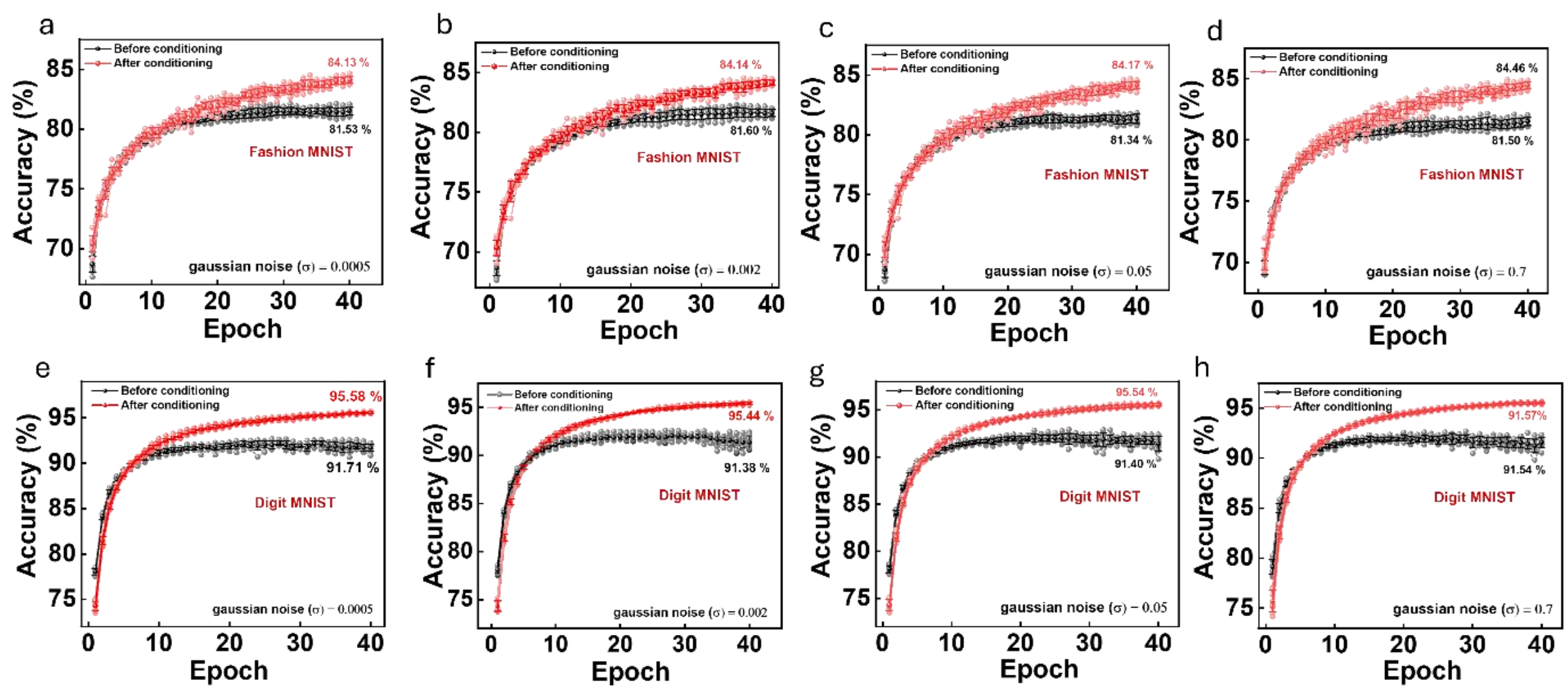

Figure S25. (a-d) depicts the evolution of classification accuracy as a function of training epochs for the Fashion MNIST dataset under varying Gaussian noise levels (σ = 0.0005, 0.002, 0.05, and 0.7), comparing performance before and after optoelectrical conditioning. Correspondingly, Figure (e -h) illustrate the accuracy versus epoch characteristics for the Digit MNIST dataset under the same noise conditions before and after optoelectrical conditioning.

To evaluate the robustness of pattern recognition accuracy before and after optoelectrical conditioning, Monte Carlo (MC) simulations were performed by introducing Gaussian noise into the device-derived synaptic weights used in the ANN simulation to simulate stochastic synaptic variability. Different Gaussian noise levels (σ = 0.0005, 0.002, 0.05, 0.7) were considered to

evaluate the robustness of the classification performance against increasing stochastic variability.[30-32]

The noise was modelled as [32-34]

$$w' = w + N(0, \sigma |w|)$$

where σ represents the Gaussian noise level, and w denotes the synaptic weight. For each noise condition (σ = 0.0005, 0.002, 0.05, 0.7), the simulations were repeated over 10 independent trials for both Fashion MNIST and Digit MNIST datasets.

The evolution of the classification accuracy of Fashion MNIST with epochs is presented in **Figure S25a–d,** with accuracy (~ 81.52 % before optoelectrical conditioning and ~84.20 % accuracy after optoelectrical conditioning, where standard deviation (SD) error bars quantify the run-to-run variability. Similarly, the Digit MNIST dataset classification accuracy with epochs and SD is presented in **Figure S25e-h** with accuracy (91.7 % before optoelectrical conditioning and 95.6 % after optoelectrical conditioning). The final classification accuracy at the last epoch was recorded for each trial, with mean accuracy and SD **(Manuscript, Figure 6).** Despite the increasing Gaussian noise levels, the classification accuracies for both Fashion-MNIST and Digit-MNIST remained relatively stable, with low SD values across all noise levels. The conditioned state shows consistent improvement in accuracy across all trials, combined with low variability, indicating that the observed enhancement is reliable and not due to random fluctuations or numerical noise.

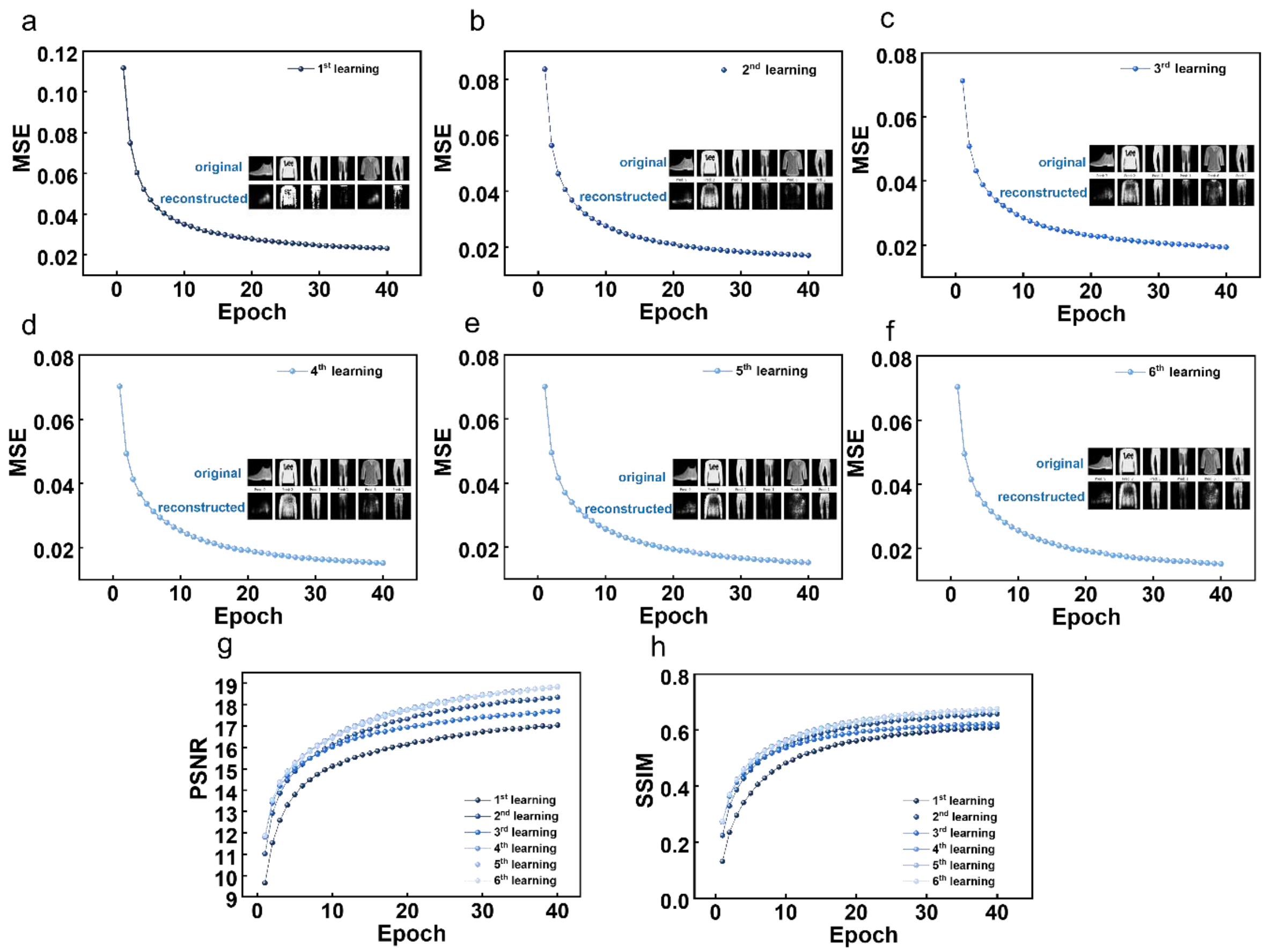

Figure S26. (a-f) Mean square error (MSE) reconstruction loss versus training epochs for each learning cycle, with original and reconstructed Fashion MNIST images shown as insets, and (g) Peak signal-to-noise ratio (PSNR) and (h) Structural similarity index measure (SSIM) curves across six learning cycles.

Figure S26 (a-f) presents the reconstruction performance metrics across all six learning cycles, shows a progressive decrease from 1st learning to 3rd learning **(Figure S26a-c),** indicating improved image reconstruction quality with each learning cycle, as visually confirmed by the inset reconstructed images. During optoelectrical conditioning **(Figure S26d-f),** the MSE curves have similar values, showing stabilized reconstruction behaviour. **Figure S26g and h** show peak

signal-to-noise ratio (PSNR) and structural similarity index measure (SSIM) to validate reconstruction performance. The PSNR increased from 17.05 dB to 18.86 dB and SSIM improved from 0.61 to 0.67 across learning cycles, indicating improved reconstruction quality of the output images.

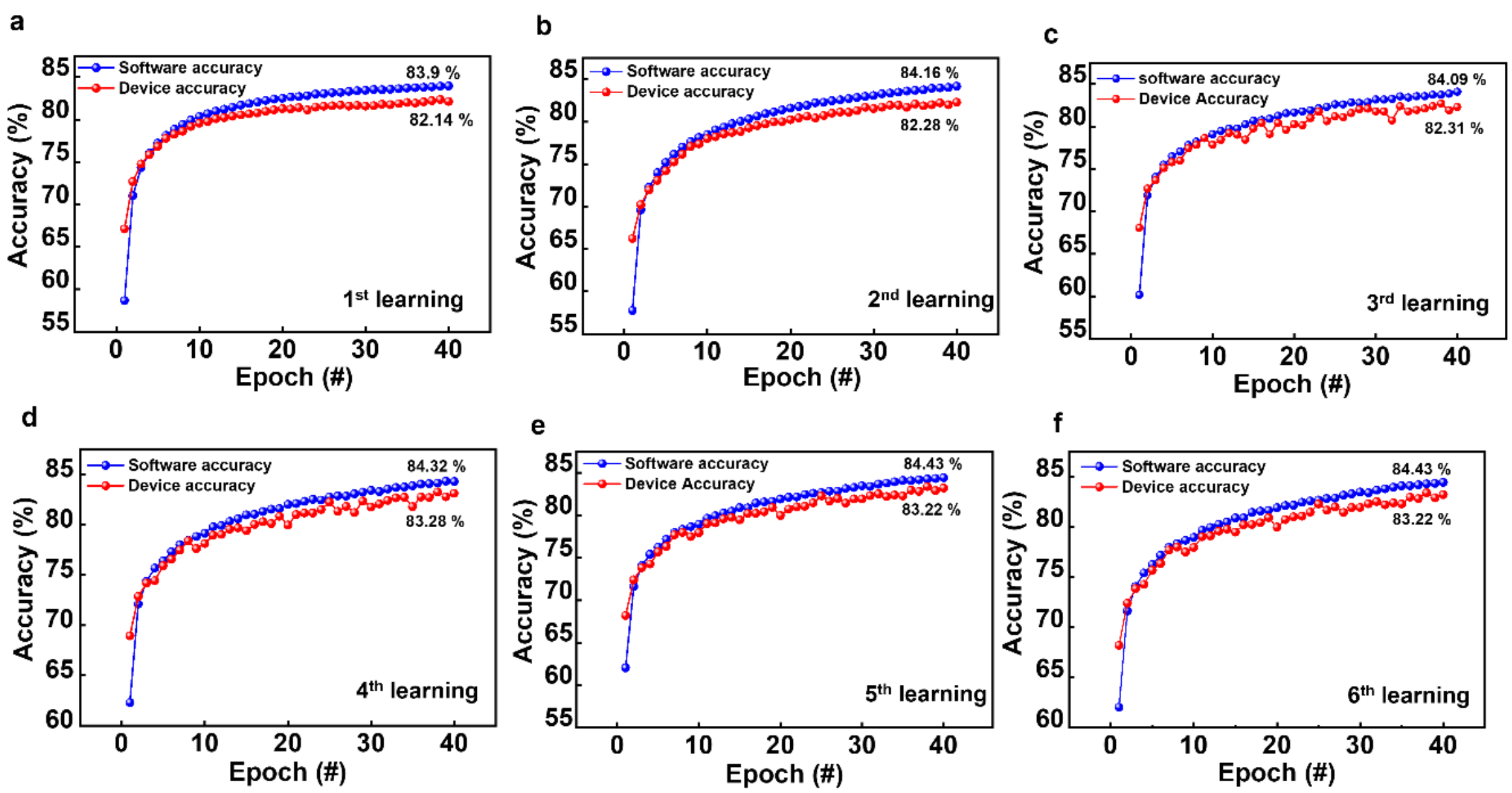

Figure S27. (a-f) The classification accuracy of the Fashion MNIST dataset from the first to the sixth learning processes, respectively.

Table S7. Performance comparison of the proposed Ag/GQD-VO_2/Ni device with recently reported papers

**Device structure**	**Switching voltage**	**Memory window**	**Energy consumption**	**Conductance states**	**Endurance and retention**	**responsivity**	**Non linearity**	**Classification accuracy**	**Ref**
Ag/Sb_2Se_3/HZO/Si	+2V,-0.9V	10	6 nJ		10^4 cycles/10^4 s	-	-	90.33 % MNIST, 83.69 % FMNIST	[35]
In/ZnO/GO/ITO	-	-	23 fJ/spike	-	-	-	-	ANN 92.84 %	[36]
Ag/MoS_2/TiO_2/FTO	1V, 5V	10	-	-	10^3 cycles/10^3s	-	0.02 (LTP), 0.018 (LTD)	ANN 98.46 %	[37]
Au/PSCO/NiO/CBTi/FTO	-2.1 V	10^2	-	5	10^3 cycles		-	-	[38]
W/WO_3/IGZO/ITO	+1 V, -1V	-	-	7	700 cycles	-	1.16	-	[39]
Ag/CdS-GeSe/FTO	+2 V, -2V	10^4	-	10	500 cycles/ 500 s	-	0.67	Fashion MNIST:75%, Digit MNIST:95%	[40]
ITO/PEDOT: PSS/CuSCN/ZnO/Al/ITO	+1.5 V, - 1.5 V	7			-	-	0.51 (LTP), 3.84 (LTD)	92.52 % (CNN)	[41]
Pd/WO_3/MoO_3/ITO	+1 V, -1	7	0.25	6	10^2 s	-	-	92.4 %	[42]

	V		mW/cm^2						
ITO/ZnO QDs/CdSe QDs/ZnO QDs/Al	+2V, -2V	10^4	-	-	-	-	-	92.59%	[43]
Ag/ITZO/Ga_2O_3/ITO	+1.5 V, -1.5V			5	10^3 s	0.88, 0.1 AW^{-1}		97.13 % (CNN)	[44]
ITO/IGZO/Al2O3	+3 V , -3V	-			-	-	2.39 (LTP), -4.88 (LTD)	89.4 %	[45]
Ag/CTS-MoO_3/Mo	+1 V, -1V	4000			200, 10^4 s	-	-	97.2 %	[46]
ITO/NSTO	+1 V, -2V	-	-	4	10^2	-	-	97 %	[47]
**Ag/GQD-VO2/Ni**	**+0.5 V – 1 V (dark) +1.5 V, - 1.5 V (light)**	**10 to 10^4**	**31.5 nJ/spike**	**6**	**500, 500 s**	**0.29 AW^{-1}**	**0.03 (LTP) and 0.02 (LTD)**	**95.58 % (Digit) 84.13 % (Fashion)**	**This Work**

References.

1. G. Silversmit, D. Depla, H. Poelman, et al., "Determination of the V2p XPS binding energies for different vanadium oxidation states (V5+ to V0+)," *Journal of Electron Spectroscopy and Related Phenomena* **135**(2004)**:** 167–175, https://doi.org/10.1016/j.elspec.2004.03.004.

2. A. Mandal,T. Som, "Oxygen-Mediated Tunability in the Physicochemical Properties of Radio-Frequency-Sputtered Vanadium Pentoxide Thin Films toward Energy Harvesting," *ACS Applied Energy Materials* **7**(2024)**:** 6845–6855, https://doi.org/10.1021/acsaem.4c01104.

3. W. Xu, Q. Ru, Z. Pan, et al., "Finely crafted lanthanum vanadium oxide cathode as durable and flexible quasi-solid state zinc ion battery," *Journal of Materials Science: Materials in Electronics* **33**(2022)**:** 5635–5647, https://doi.org/10.1007/s10854-022-07750-8.

4. D. Ielmini,G. Pedretti, "Resistive switching random-access memory (RRAM): Applications and requirements for memory and computing," *Chemical Reviews* **125**(2025)**:** 5584–5625, https://doi.org/10.1021/acs.chemrev.4c00845.

5. S. S. Kundale, P. S. Pawar, D. D. Kumbhar, et al., "Multilevel Conductance States of Vapor-Transport-Deposited Sb2S3 Memristors Achieved via Electrical and Optical Modulation," *Advanced Science* **11**(2024)**:** 2405251, https://doi.org/10.1002/advs.202405251.

6. J. Xie, X. Shan, N. Zou, et al., "All-optically controlled memristive device based on Cu2O/TiO2 heterostructure toward neuromorphic visual system," *Research* **8**(2025)**:** 0580, https://doi.org/10.34133/research.0580.

7. S. Shim, S. Kim, D. Lee, et al., "Infrared-Triggered Retinomorphic Artificial Synapse Electronic Device Containing Multi-Dimensional van der Waals Heterojunctions," *Small* **21**(2025)**:** 2410892, https://doi.org/10.1002/smll.202410892.

8. Z.-H. Tan, R. Yang, K. Terabe, et al., "Synaptic metaplasticity realized in oxide memristive devices," *Advanced Materials (Deerfield Beach, Fla.)* **28**(2015)**:** 377–384, https://doi.org/10.1002/adma.201503575.

9. J. Yin, F. Zeng, Q. Wan, et al., "Adaptive crystallite kinetics in homogenous bilayer oxide memristor for emulating diverse synaptic plasticity," *Advanced Functional Materials* **28**(2018)**:** 1706927, https://doi.org/10.1002/adfm.201706927.

10. Q. Wu, H. Wang, Q. Luo, et al., "Full imitation of synaptic metaplasticity based on memristor devices," *Nanoscale* **10**(2018)**:** 5875–5881, https://doi.org/10.1039/c8nr00222c.

11. B. Liu, Z. Liu, I.-S. Chiu, et al., "Programmable synaptic metaplasticity and below femtojoule spiking energy realized in graphene-based neuromorphic memristor," *ACS applied materials & interfaces* **10**(2018)**:** 20237–20243, https://doi.org/10.1021/acsami.8b04685.

12. X. Fan, A. Chen, Z. Li, et al., "Metaplasticity-Enabled Graphene Quantum Dot Devices for Mitigating Catastrophic Forgetting in Artificial Neural Networks," *Advanced Materials* **37**(2025)**:** 2411237, https://doi.org/10.1002/adma.202411237.

13. Z. Gao, X. Ju, H. Zhang, et al., "InP quantum dots tailored oxide thin film phototransistor for bioinspired visual adaptation," *Advanced Functional Materials* **33**(2023)**:** 2305959, https://doi.org/10.1002/adfm.202305959.

14. S. M. Kwon, S. W. Cho, M. Kim, et al., "Environment-adaptable artificial visual perception behaviors using a light-adjustable optoelectronic neuromorphic device array," *Advanced Materials* **31**(2019)**:** 1906433, https://doi.org/10.1002/adma.201906433.

15. Y. Cheng, J. Zhang, Y. Lin, et al., "Bioinspired adaptive neuron enabled by self-powered optoelectronic memristor and threshold switching memory for neuromorphic visual system," *Advanced Science* (2025)**:** 2417461, https://doi.org/10.1002/advs.202417461.

16. L. Su, Z. Hu, T. Yan, et al., "Light-adapted optoelectronic-memristive device for the artificial visual system," *ACS Applied Materials & Interfaces* **16**(2024)**:** 43742–43751, https://doi.org/10.1021/acsami.4c07976.

17. G. Gong, Y. Zhou, Z. Xiong, et al., "An antagonistic photovoltaic memristor for bioinspired active contrast adaptation," *Advanced Materials* **36**(2024)**:** 2409844, https://doi.org/10.1002/adma.202409844.

18. F. Farooq, P. Kaith,A. Bera, "Photo-Induced Oxygen Vacancy Modulation in Solution-Processed TiO2/ZnFe2O4 Heterointerface for Dual-Mode Synaptic Memory," *Advanced Materials Technologies* (2026)**:** e70995, https://doi.org/10.1002/admt.70995.

19. L. Fan, J. Li, X. Su, et al., "Optoelectronic Synergy Reservoir Computing System Based on CuI-TiO x Heterojunction Memristor for High Precision Image Processing," *ACS Applied Electronic Materials* (2026), https://doi.org/10.1021/acsaelm.6c00484.

20. Y. Xiong, Z. Jiang, P. Jiao, et al., "Interface-engineered LuOx/Cu_3 (HHTP)$_2$ heterojunction with MoO_3 interlayer for high-performance artificial optoelectronic synapses and in-sensor computing," *Chemical Engineering Journal* **531**(2026)**:** 174194, https://doi.org/10.1016/j.cej.2026.174194.

21. Y. Dai, S. Hao, G. Feng, et al., "A self-powered organic vision sensor array for photopic adaptation," *Nano Letters* **25**(2025)**:** 2878–2886, https://doi.org/10.1021/acs.nanolett.4c06090.

22. C. Mahata, M. Ismail, H. Kim, et al., "Optically Tunable Synaptic Plasticity and Memory Emulation in Au-Nanoparticle Enhanced HfTiOx/Al2O3-Based Photonic Memristors," *Advanced Functional Materials* **36**(2026)**:** e10663, https://doi.org/10.1002/adfm.202510663.

23. X. Lin, Z. Lin, W. Zhao, et al., "A back-to-back structured bionic visual sensor for adaptive perception," *Applied Physics Letters* **126**(2025), https://doi.org/10.1063/5.0268992.

24. M. Jain, B. F. CK, M. Khemnani, et al., "Engineering of A-site cations in APbI3 (A= Cs, Rb, K) perovskites for resistive switching control and self-rectifying memristors for next-generation computing applications," *Nano Energy* **138**(2025)**:** 110871, https://doi.org/10.1016/j.nanoen.2025.110871.

25. D. Hu, R. Yang, H. Lin, et al., "In-sensor polychromatic visual information processing enabled by all-optical controlled neuromorphic devices," *Nano Energy* (2026)**:** 111715, https://doi.org/10.1016/j.nanoen.2026.111715.

26. X. Yang, D. Wang, Z. Zheng, et al., "Large-area InSe nanofilm for artificial optoelectronic synapses with ultra-low energy consumption," *Nano Energy* (2025)**:** 111600, https://doi.org/10.1016/j.nanoen.2025.111600.

27. S. Lee, H. Jang, G. An, et al., "Synergistic multi-wavelength optical stimulation enhances synaptic dynamics and reservoir computing performance in ferroelectric thin-film transistors," *Nano Energy* (2025)**:** 111395, https://doi.org/10.1016/j.nanoen.2025.111395.

28. H. Aziza, M. Fieback, S. Hamdioui, et al., "Conductance variability in RRAM and its implications at the neural network level," *Microelectronics Reliability* **166**(2025)**:** 115594, https://doi.org/10.1016/j.microrel.2025.115594.

29. S. Kunwar, Z. Jernigan, Z. Hughes, et al., "An Interface-Type Memristive Device for Artificial Synapse and Neuromorphic Computing," *Advanced intelligent systems* **5**(2023)**:** 2300035, https://doi.org/10.1002/aisy.202300035.

30. B. Jeong, P. H. Chung, J. Han, et al., "Enhanced linear and symmetric synaptic weight update characteristics in a Pt/p-LiCoO x/p-NiO/Pt memristor through interface energy barrier modulation by Li ion redistribution," *Nanoscale* **16**(2024)**:** 5737–5749, https://doi.org/10.1039/d3nr06091h.

31. J. Xu, Y. Zheng, F. Li, et al., "Modeling cycle-to-cycle variation in memristors for in-situ unsupervised trace-STDP learning," *IEEE Transactions on Circuits and Systems II: Express Briefs* **71**(2023)**:** 627–631, https://doi.org/10.1109/TCSII.2023.3309329.

32. S. Kim, H.-D. Kim,S.-J. Choi, "Impact of synaptic device variations on classification accuracy in a binarized neural network," *Scientific reports* **9**(2019)**:** 15237, https://doi.org/10.1038/s41598-019-51814-5.

33. C. Li, R. Chen, C. Moutafis, et al. In *Robustness to noisy synaptic weights in spiking neural networks*, 2020 International Joint Conference on Neural Networks (IJCNN), IEEE: **2020**; pp 1–8.

34. A. J. Ford,R. Jha In *Memristive device variability performance impact on neuromorphic machine learning hardware*, 2020 11th International Green and Sustainable Computing Workshops (IGSC), IEEE: **2020**; pp 1–7.

35. S. Jiang, Y. Yang, J. Gu, et al., "Robust Sb2Se3/hafnium zirconium oxide synaptic memristors with dynamic learning rate strategy for generalizable neuromorphic networks," *Advanced Functional Materials* **36**(2026)**:** e27371, https://doi.org/10.1002/adfm.202527371.

36. Z. Zheng, B. Qiao, Z. Han, et al., "Multi-defect-engineering in ZnO/GO heterostructures for optoelectronic synaptic devices with ultra-high dynamic range and low energy consumption," *InfoMat* **8**(2026)**:** e70089, https://doi.org/10.1002/inf2.70089.

37. N. Malishetty, V. Chandmare, N. Vadthiya, et al., "Interfacial ion dynamics and synaptic behavior in Ag/MoS2/TiO2 heterostructure memristor for neuromorphic applications," *Materials Science in Semiconductor Processing* **212**(2026)**:** 110761, https://doi.org/10.1016/j.mssp.2026.110761.

38. W. Liu, C. Zhang, Z. Jiang, et al., "PSCO/NiO/CBTi heterostructure memristor with multi-level resistance states for neuromorphic and biomimetic synapses," *Applied Materials Today* **51**(2026)**:** 103354, https://doi.org/10.1016/j.apmt.2026.103354.

39. S. Jia, H. Lu, Y. Zhu, et al., "Robust and High-Performance Self-Rectifying Memristors Featuring a-WO3/a-IGZO Heterostructures for Reliable Artificial Synaptic Applications," *The Journal of Physical Chemistry Letters* **17**(2026)**:** 5258–5265, https://doi.org/10.1021/acs.jpclett.6c00943.

40. G. U. Kamble, S. S. Kundale, J. S. Jang, et al., "CdS-GeSe Heterostructure-Based Bimodal Memristor with Tunable Synaptic Plasticity and Mixed-Signal Switching for Neuromorphic Hardware," *Small* **22**(2026)**:** e07053, https://doi.org/10.1002/smll.202507053.

41. Q. Lin, Y. Zhu, X. Hu, et al., "Multifunctional PN optoelectronic synapse and its smart integration towards augmented artificial visual system," *npj Flexible Electronics* **9**(2025)**:** 81, https://doi.org/10.1038/s41528-025-00459-8.

42. J. Jiang, Y. Liu, J. Li, et al., "Multifunctional ultralow-power-consumption artificial optoelectronic synapses based on the heterojunctions of MoO3/WO3 for neuromorphic

computing and bionic visual systems," *ACS Applied Materials & Interfaces* **17**(2025)**:** 64743–64752, https://doi.org/10.1021/acsami.5c16987.

43. Q. Lin, Y. Zhu, J. Sun, et al., "A Full-Quantum-Dot Optoelectronic Memristor for In-Sensor Reservoir Computing System with Integrated Functions," *Advanced Functional Materials* **35**(2025)**:** 2423548, https://doi.org/10.1002/adfm.202423548.

44. Y. Lin, L. Zhong, L. Wang, et al., "ITZO/Ga2O3 heterostructure memristor with integrated optoelectronic synaptic function for neuromorphic vision applications," *Chemical Engineering Journal* (2025)**:** 169684, https://doi.org/10.1016/j.cej.2025.169684.

45. S. Song, M. Kim, G. Yoo, et al., "Solution-processed oxide semiconductor-based artificial optoelectronic synapse array for spatiotemporal synaptic integration," *Journal of Alloys and Compounds* **857**(2021)**:** 158027, https://doi.org/10.1016/j.jallcom.2020.158027.

46. W. Wei, H. Sun, X. Dong, et al., "A neotype self-rectifying Cu3SnS4-MoO3 synaptic memristor for neuromorphic applications," *Chemical Engineering Journal* **482**(2024)**:** 148848, https://doi.org/10.1016/j.cej.2024.148848.

47. H. Fang, S. Ma, J. Wang, et al., "Multimodal in-sensor computing implemented by easily-fabricated oxide-heterojunction optoelectronic synapses," *Advanced Functional Materials* **34**(2024)**:** 2409045, https://doi.org/10.1002/adfm.202409045.
